# Supplementary material for: Neutering of cats and dogs in Ireland; pet owner self-reported perceptions of enabling and disabling factors in the decision to neuter
Source: PeerJ. 2015 Aug 20;3:e1196. doi: 10.7717/peerj.1196 (PMC4548501; doi:10.7717/peerj.1196)
Supplement: Supplemental Information 1 [file peerj-03-1196-s001.pdf]

Contents

Location 1 1

Location 2 48

Location 3 focus group 1 127

Location 3 Focus group 2 211

Location 4 222

Location 5 255

Location 6 303

This file contains the raw transcription text from each of the Focus groups, the data has been anonymised.

Location 1

Modulator

Basically I am going to go anti clockwise.

And so Participant1 can you tell me one or a few of the things that came up in your discussion about why you own a pet

Participant1

We got the first one from a shelter, I think we just ...Why did we get him?

Participant2

You wanted a cat and I said Yes . I think the conversation we had, I didn't know why we had pets but we do

Participant1

I think they are nice, they are company. Yeah because the cat is not there to catch mice or anything. Or protection either

Participant2

They are not working animals

Participant1

I would say for company Yeah

Participant3

I am the same as Participant2 I didn't know why I wanted a pet either I just decided to get a cat from the GSPCA. Participant4 used to mind the cat originally she's a feral cat

I decided to get one cat and now we have four

Participant2

I had a cat as a kid

I did too

I think that is it, if you have a pet as a kid you have memories of that

Yeah I think that, we were just saying that as well. I had cats, I think the animal you have as a child is often the one you get as a grown up, it's the animal you like maybe

Modulator

Participant4 why do you have so many pets

Participant4

The reason it started back In 1974 and I was living in England and I wanted to get something form a very large dept store there was a bomb scare. Rather than stay outside i wandered along to the pet shop. They asked if they could help, and I said no no I am just looking. Looking for kittens thinking they'd be in cages somewhere, they weren't . They brought three male kittens out , they said male or female> I said male and it took three quarters of an hour to get one off me so I went home with him. Never did remember what i wanted from the shop in the first place

Had him for 17 years on his own when he died . So we decided that was it, and so no more, we were heart broken. A few days later we decided we were being selfish and that we could give a cat a good home so we got a brother and a sister. And then after that there was a sign over the door which said if you were ill and homeless and needed someone to look after you to come here

Modulator

Fair enough

Participant4

We brought the 9 cats we had in UK with us , we had always decided to get a dog when we came over here but that's actually grown too. Now we have 13 cats and 3 dogs only got two of .... we brought 12 of these over with us as well

Modulator

Why did u get a dog as well

Participant4

We couldn't have a dog in UK what with both of us working in the city, even this dog we didn't go to choose here we got her at about 6 months old and we were here third home a relative said would you like her We were going to get a dog anyway ,and one dogs the same as another so we got her and she is probably the most intelligent creature I have ever met and the other dog came along because he had been chucked in a barn to die when he was three months old. Someone got him out. We ended up with him, and the last dog came because he was actually walking around Location 1with a bad eye, in a bad state and all the rest of it. Someone had phoned the GSPCA. The field officer was off sick that day, she'd gone off and I thought I'd have a look, I took her home for the night, about 2 years ago

Modulator

Mother Teresa it is them

Participant4

I had a beautiful whippet that was left behind by gypsies, I had her for a couple of months. She lives very happily now over near Spiddal border. Mind you it broke my heart giving her up

Modulator

Participant5 what..

My story in't as interesting it's like the advert. I like my dog and my dog likes me, I took early retirement about 8 years ago and I decided I would get a poodle and that Iis what i did . Lucky is the dogs name a female, a great little character again very intelligent

Modulator

Was there a particular reason, ws it just company or?

Participant5

Company I suppose to get me out , I am originally from Location 1and I worked away for 30 years then I came back and I thought what if arna had changed and I didn't really know anyone here now. I'd say i'm the only Location 1person here I know no one else but when i come back I will bring the dog for a walk and every one says hello to lucky and nobody says hello to me That was one of the reasons, to get me out

Ah She is great company and before I came out tonight my young nephew who is staying with me asked are you going to tell the truth and tell them where the dog sleeps . Whatever they ask me I will tell the truth. SO the dog sleeps in the bed, when the news stars at nine o'clock the gets up to the kitchen door, it's silly. You open the door. She hops up the stairs and waits til I come up. I suppose that is bad training is it?

Modulator

Whatever helps

Participant5

A very non stressful life

Modulator

Good Good

Participant6 you are keeping quiet down there

Participant6

I only have 2 dogs and a cat not a lot

I have always loved animals from as far back as I could remember A cow, wild geese chickens a pig It was always lovely to feel, lovely to touch them Have u ever stroked a pig? It's amazing yeah so my mother was devastated when I became a vegetarian. I couldn't bear the thought of eating them, you know to love animals it is weird to eat them .But anyway, today two dogs and .... cats

.... my cat he is a huge he is the boss in the house ,I don't think he hunts anything all he sees is the fridge. I think that i what i love about cats, they are so selfish > They love themselves and in contrast to the dogs. They are so selfless and caring, loveable, do anything for you. Dogs are just the total opposite to cats

They are both seven. They love each other's company Purdy is quite a big dog, i got her from the Gspca. I said I'd take a little dog, there was three of us sharing a house and we all took measurements of the coffee table to see how big she would get, she is adorable She is a rescue dog and she is wild She is lovely and they love each other lovely to live with, peaceful, happy

They can be demanding walks , n to walk the dog no matter how tired, you have

I just couldn't imagine living without an animal or several animals The way life is

Modulator

What about you Participant6

PARTICIPANT6

I have always had pets ever since I was a kid between rats and hamsters, we always had at least two dogs when i was at home. I moved into to en and I minded dogs I didn't have time

Some dogs you'd mind for a month and then it was like giving it away and I said no more minding dogs, so i got a little terrier and rescued a rotty about 3 years ago and so

The terrier had pups a few weeks ago so I am hand feeding the pup now, probably keep her too

ENTER PARTICIPANT7

Modulator

So would you like to continue on Participant6

Participant6

That is about I it I am now stuck with 3 dogs

Modulator

And why do you think you got the dog in the first place? Is it because you have always had a pet

Participant6

Yeah Well I was minding a pug, I got really attached to and giving him back was hard so i said no more minding dogs and I got the terrier. The I over heard about 2 years ago/ later that someone was going to put down a rotty only 4 months, he was up in Dublin and I knew someone in Conemara and I said if I couldn't mind him with the terrier, she's a brat. I said oh no she'll start and he'll eat her, but the rotty is so nice, so gentle a really nice dog, a beautiful animal.

Modulator

Participant3 do you have anything to add, you kind of got her for companionship

No it just the 4 cats is where i'll stop I keep the inside they don't go out

Participant3

Now it's just the four cats is where i'll stop, I keep them inside all the time I don't let them out at all. I got Tammy and I was told she wouldn't be a lap cat, and I wanted a lap cat so I got another cat and they all turned out to be lap cats now

Modulator

You kept going til you got a lap cat, very good

You are ok with your answers Participant9 and Participant2 are you?

Participant9

I had cats, I've only one now. I have cats cos i had cats growing up they are incredibly beautiful animals and I get a lot of pleasure just looking at them and what they get up to, entertaining. Also the physical contact with the animal, I really enjoy that, you know what I mean. These would be my reasons for having a cat

Modulator

Basically Participant7 I am doing a study I have done a certain amount of questionnaires/surveys. After the survey we felt we might have missed stuff out, the questionnaire is just ask a question and give an answer. The reason for having this focus group is to find out what people's opinions are. How they feel about pets? Owning pets and stuff like that. We've a list of questions to go through. Hopefully we get through it. WSe are moseying on through. O why do you have a pet in the first place?

Participant7

Really a companion for the child, she really wanted a pet, she really wanted a pet. Not saying the rest of us didn't want a pet but she was the driving force behind it. We had a misfortune with it, so went off at Xmas and got 2 maltese dogs, they're great fun they take a lot of time, we don't get the contribution we were promises at the start. They became part of the family really

Modulator

To a certain extent we have answered the second part. Role u see your pet play in your life

Participant5

Unconditional love, the main thing, the welcome you get when you've been out, the welcome you get when you come back in. And the poodle doesn't shed that's great she is in a lot

Modulator

Do you suffer from allergies or anything like that or is it just not hovering the couch each morning

Participant5

I retires, I told you that. Hoovering or ironing wouldn't be one of the things I retired for. That was the reason

Participant9

Becaus ethey can live alone. I don't have to walk them. You can leave a cat at home when you are going to work. A dog would be difficult especially with shift work or I suppose planning ahead, I got her while I was still studying and I had a cat when I was a child I knew what a cat needed

Participant2

And who looked after the cat when u were out of the house, when you were at uni everyday and I was East Bonn

Participant9 at a time

It was only a few hours

Modulator

So you have a different reason for getting the cat Participant2

Participant2

We were living in Germany in a flat a small flat compared to where we have lived since. I can't imagine us having a different animal maybe fish

Participant3

Cats though are more independent, easier to manage. Cats sleep a lot and easy to toilet train

Participant4

A lot better than a dog

Participant3

You just couldn't leave a do g and go out all day, in the house

Modulator

So Participant6, you seem to be the odd man out Participant6. Why do you have a dog

Participant6

I always had dogs growing up, we had a ct as well, But the dog I was minding them and then out and about with other peoples dogs, I said to myself I got a dog so I can go for walks in the evening and get out of the house

Modulator

Participant8, why did you choose both types of animal?

Participant8

I guess i grew up with dogs in the house You can just live so easily with them. What was the question? They're just adorable, theyre so domesticated.

Modulator

And Do they live easily together

Participant8

Oh Yeah they are great, but the cat is the boss, if it decides to sleep in one of the dogs beds, the dog sleeps on the floor. Yeah they get on great, the three of them

Modulator

Great Go to Participant9 now

Participant9

Why did I particularly chosose a cat, well, like I said I grew up with cats, I just think they are gorgeous. I know that people say you are a cat or a dog person or both. I would class myself as a cat person. But I was saying to Participant2 I'm getting to like dogs more and maybe getting a do in the future. But cats are so easy less responsibility. Obvoiusly still a burden but less responsibility because they are so independent, than having a dog. I like that about a cat, when it needs you it will come looking for you otherwise leave me alone. I just think they are gorgeous animals and easy to look after

Modulator

What about you Participant4? Your full range of.....

Participant4

I have got no Idea why i pretended I wanted to look for a kitten in that shop, don't ask me why. The reason I was stuck with cats when I was in England because of working Like everyone said. Cats are so much easier, looking after themselves when you are at work. That wsa one of the reasons we said we would like to get a dog, my husband wanted a dog more for going out for walks, and don't have to expect when he is not there, that is why we got the first dog and with it the cats. The other dog came along. I must admit our first dog is..................... our dog is graet cos she will collect the cat that needs to go to the vet, with the exception of one. Noone will go near that cat Yeah Like everyone said they give unconditional love, they don't care what you look like in the morning and don't ask for designer trainers or anything like that

Modulator

Yeah That is true so no designer GAP clothes

Participant9

The animal you grow up with you know about. You know with a dog I suppose. I wouldn't know what to do with a dog

Modulator

What about your household Participant7?

Participant7

I grew up on a farm My father always had dogs , sheep dogs and gun dogs and we always had them for sheep and cattle and that was part of growing up, we fished and did shooting Ursula wanted a dog. I had a lot of experience with animals over the years, you know because we grew up on a farm. We had a german pointer when we were building the house, we had the misfortune the builder came in and ran over him. I remember we had a red stter he was my pet growing up and used to hop on the tractor beside me, next thing he jumped off and under the wheel and you had to pick him up and bring him home and dig a hole and bury him and stuff.We bought a dalmation dog for our son, he grew p and he pulled us around the place. He wasn't getting the exercise he needed so we made sure he got a proper home, then we had a small littlwe white dog cross, we didn't have much luck with him either but I was very attached, didn't have success saving him

We had a black cat at the same time they used to play in the garden chasing one another around, Bizarre to watch the two of them. After the accident the cat died shortly afterward, my wife often thought it was lonely. We bought here in and tried to keep her warm but it died. A lovely black cat

We have two Maltese terriers I get into big trouble over bringing them for walks, they come back black, knots in their hair, I'm always in trouble e with them Other than that they are delightfull little things very inteeligent and I enjoy them immensely, she calls them M and M, two white rappers

Modulator

Brilliant, OK. I suppose we will move on a little bit. I hope this stars a bit of an arguement now, get ye all fired up

I was just looking for your views on neutering. I don't know wheter you all have your pets neutered but you wanna talk Participant4

Participant4

Yeah me. They should be neutered unless there is a good reason for breeding from themand I really think, I personally think that if you are going to breed from animals there should be some sort of law, a licensing regulation , so that people can't just let them beed and interbreed and then you end up with lots of problems

I know If that was in existence Tammy   would never be in existence he was as complete feral. I mean I have got feral at home that was stated with him. Ok , generations back if everyone had neutered their cats these ferals wouldn't be about but on the other hand even with people neutering them You're not goig to get all of them but u will cut down on a lot of suffering , what ferals and strays go through in all breeds, wheter it is cats o dogs whatever

Modulator

Does everyone agree or have any other ……?

Participant3

Yeah I agree with Participant4, cats seem to mark their territory Keeping them indoors I'd have a pretty smelly home if I didn't neuter them, you know

Modulator

Why did you neuter your cats ?

Participant3

One tabby was neutered when iIgot it and I decided to neuter the others because they would mark their territory and probably fight more. SO they are all neutered

Participant2

I prefer to keep cats indoors too, we keep our three indoors so if they were not neutered they'd want to go out a lot more, especially if there is a cat in heat in the area, they'd be quite keen to go out .

Participant9

They are all boys so I know they would fight a lot, threy fight as it is

Modulator

What about you Participant5 as a dog owner?

Participant5

When the dog went to the vet, *The vet* wasn't here at that time the vet advised be that unless I was going to breed the dog I would be better off getting the dog neutered, as well as that I would be the type of person that If my dog had puppies I'd still keep all the puppies. I couldn't visit one of the homes or shelters and I couldn't give the pups away, the telly is enough for me, you know the ad with the dog looking out through the cage

Modulator

So it was because of what your vet said to you that you did it ?

Pasty

Yes I would be afraid of a big dog coming near this little one and she so frightened as a result

PARTICIPANT7

I suppose I did I grew up on a farm I probably have a very different spin on animals and stuff like that

I remember going to the market, rear them hand feed them with a bucket then sell them you grow up with a different thing I remember we had a cow she had to and she had to be put down, she was due to calve. We brought her to the factory. They shot the animal I was very young at the time and my job was to take the calf and bring it back and put it in straw and feed it with a bucket

I have a very different slant on animals I certainly wouldn't be cruel but certainly I'd understand they have a working purpose as well as a pet purpose

Pasty

We are living in a different world I grew up a bit like that too. I remember Location 1being a village, I remember the cows going along the road

Participant7

I'm not saying I'm hard to it

Modulator

Are you pets neutered? Would you rather leave them?

Participant7

No they are just coming into season, they ae six months old. *The vet* has asked us to do one of them

Modulator

Would you like to leave them?

Participant7

The dogs are there as pets when it come s down to making the decision I have made enough decisions, that's up to Ursula. The last dog I had i found him at Croke Patrick when he was small, he was getting tired I put him in my rucksack. I lost him. That's the end of dogs in this house but I had to get another. In terms of neutering I have n desire for them to have pups to make money, Ursula would like maybe

Participant5

Do you find it difficult to give them away

Child

Em not really. I might ask to keep one but I know what the answer would be straight away

Participant5

I'd say it'd be hard to part with them

Modulator

What about down this side of the table, are ye all neuterers or do you have different views

Participant6

The terrier I didn't neuter her I always planned to breed her, the rottweiller I had him neutered,I plan on neutering her now. She had puppies three weeks ago, four pups three of them died a she had 4 pups 3 of them died and everyone is saying will i keep it or give it away. I finding it very hard to say ah no i'm gonna sell it, so I'll probably end up keeping it

Modulator

Will you leave her attached Participant6 Yeah

Participant8

All neutered, a male cat I had him neutered cos I didn't want thim to catch feline aids. The dogs I had them neutered because I wouldn't breed from them and they are pets it can increase their lifespan a couple of years longer maybe selfish bu I'll hang onto them as long as possible

PARTICIPANT9

Yeah my cat is neutered, it was in my head that that is just what you do I don't want the responsibility of finding home s for kittens andand also one of my cats that I still have did go into het once before she was neutered and I had never seen that before

How wierd for us

The thing of too many kittens ans, though if her parents had been neutered then we wouldn't have her, don't know how to balance that, that their lives canbe extended and just to keep her out of trouble and it's not that much fun for female cats really. So mostly it is the responsible thing to do. It's what I had in my head

Modulator

Do you fell theres any other influence, did your vet practice, TV ad or anything else influence yourdecision

Participant9

I think it was in my head, I would do any animal I had I think I would do it as a matter of form

Participant2

I agree with that, I think for me it would have been years of ball park or propaganda in the states. Publicly states to have your pets spayed or neutered. At the end of that days the press is right

Participant9

There is a problem there is too many strays and that. If there was too few cats or animals then you would say different.

Participant2

In the states they destroy so many animals every year because of over population

Modulator

DOES anyone else feel that way ?

Is it because of what you have heard yourselves?

Is it past experience , does the media have any impact atr vet surgeon have any impact on what your decisions are

Participant7

The vet would make the decision for us

Modulator

Right fair enough

Child

She did say the female was quite small to have pups

Participant2

The male cats, it keep their heads smaller

Participant9

Oh Yeah don’t they look so different

Participant2

Having them neuteres keeps their heads smaller

(LAUGHING)

Modulator

I never heard that one as a reason but there you go

Participant2

I think it is te only reason to do it

Modulator

It's a headline makes- TO get his head through the ct flap

Participant4

Also there is another reason I just thought f with cats and dogs they are prone to straying. Their territories are smaller than if they are unneutered

Modulator

Is everybody happy that we have covered that

Participant7

We do have a ginger tom cat , coming and going for years. Every time he come back Theresa always feeds him. He comes back battered and bruised, then he gets so big and so lage, he stays around for a period of time then he disappears, he is still alive. The next thing is he'll appear out of nowhere all cut and scraped. You can't catch him and you can't can't put a hand on him, he is jus really wild he's getting old now and he is still coming there . *The vet* has said if she could catch him , give him a tablet put him to sleep then you could grab him, put him in a box and bring him down. I don't have the heart to do taht incase he goes out under a car after getting the pill and we'd find him in a field. He's roaming around

He has been around 10 years there was a little black kitten, he was minded and you could sit down and stroke him, he didn't last as long , we had the black cat neutered, that stopped him goingbut if you look at the two of them into their lifestyles. The ginger cat doesn't have as good a life in the sense he is not in the house and all that, he is very healthy,He's out there and he is strong. He has been around a long long time. He comes back and seems to fatten himself up then you wouldn't see him for two months, then he's back again all battered and bruised. I don't know how he keeps going (LAUGHS)

Modulator

There's a lot of bruised Ginger cats around

Child.

He did come back over the hill and he had five or six kittens coming behind him

Participant7

He was bringing a few of them up. He reckoned they all follow him up the drive. Remarkable cat really. I don't know how long he is going to be around but i'd like to put him down now because he is getting old, he doesn't move as well as he used to. I haven't the heart to be giving him pills to knock him out and bring him down here I've had enough you know, I'm not up for that stuff to bring him down to the vets I'll try and deal with it myself

Modulator

It actually brings us nicely to feeding cats up and stuff like that. We were just kinda wondering about the diets of your pets

What do u feed your pets ? Do you feed bags of dog food and cat food? Do you make it up from scraps? Do they get both?

Participant5

Royal Canisne Vet Diet Skin support , is that right? I spoke earlier about my little nephew, when I said i was coming to a group, he said, when I ca going to say the dog lies on the bed, I said Ah yeah. He said I'd forget what food the dog eats . he wrote it down in case i forget . Thats it. Royal Canine Vet Skin Support, she gets an odd bit of chicken and other than that nothing else

Modulator

Is that all he gets Participant5 or does he get?

Participant5

Milk and water , mostly water. Nearly all water and sometimes ....

Last year when I bought the dog first the ad was that she as apricot not a white poodle. when i picked her up she looked creamy as a little pup she looked creamy, she was never apricot but last year for some reason her paws started to go apricot.I thought you know she was changing colours as she got older or whatever. I discovered anyway that going down to the sea you know down to the sand. After being down on the sand she licked her paws a lot and saliva turned her skin brown apricot colour. So her four paws were that colour. But Since i stopped then I got the skin support diet and since I stopped bringing her onto the sand her paws have gone back to her creamy colour again. I though she'd be lovely apricot. Is that true that saliva changes the colour of the skin?

Participant8

Just with poodles

Modulator

With white animals you see it more than anything else

Participant5

They were like golden shoes, you know they were gorgeous but it wasn’t fair

Modulator

You’d often see black cats as well they go a kind of coppery colour they get a skin problem from licking excessively

Participant7

And what is it? Is it from the salt

Participant5

I think it is the sand the little grains of sand on her paws or something

Participant7

A reminder not to bring the dog on the sand

Participant5

The vet told me if I just put her four paws in salt water, salt and water after I came back but I didn’t think of that every time. We don’t bring her down on the actual sand

Modulator

What were u feeding her before that?

Participant5

Em..The same dog food but not the skin support

Modulator

And does every body feed their dogs .... Has anybody got?

Participant6

I feeed them nuts with tins of dog food and mix it in I find it’s cheaper to get mince meat and cook it up. In Dunnes stores you always get the cheap reduced sections of chicken or something like that. It’s

probabbly better than the dog food. The tins of dog food. But it was always nuts only recently I started giving them the extra

Modulator

And why did you do that

Participant6

Was when the bitch was pregnant, I really just noticed how cheap they were in Dunnes and those kind of sections you know. I just thought it would be better . Sometimes I go to Aldi and get the tins of dog food for them. It just works out cheaper feeding them the mince than the tins

Participant7

My puppies love bran flakes in the morning, I throw them on the floor and they eat them up

Participant5

The poodle eats very little. You know the normal bag of food, that'll last two months. Very little

Child

We normally feed the dogs royal canineas well but one of them, the boy is relaly fat and he starts stealing all the food and she is left all skin and bone, so dad started buying tins of dog food and mum started buying sausages to put for her

Participant7

We started feeding them seperatley cos he looks like he is bullying her you know, I mean once he gets near the tin it is gone . I need to fatten her up because she's got very very thin. And it's not a problem in terms of rigidity and stuff, shes a flyer but i'd say it is because he was getting the lions share all of the time. I don't get involved that much but it was something I noticed, so we are separating them now, different rooms to feed until she gets stronger

Modulator

You were saying yougive them some bran flakes is that just as a treat or...?

Participant7

They like a few in the morning so i just throw a few down to them. They love them ....

Participant5

They love being hand fed as well rather than going to the dish, eat it out of your hand

Participant7

We give them nuts nw in the bowland they wouldn't bother with them but put it into your hand and they will eat it

Participant8

With my two dogs one of them loves to be hand fed, she is a very fussy eater, she's very finicky, sometimes you just have to. If she has not eaten for two days I just have to hand feed her where the other dog will literally eat the hand off you, and you have to watch them constantly co the bigger dog will just eat everything

PARTICIPANT7

Thats right yeah

Participant8

And the other one...... anything thats left behind the other dog gets it so she a bit overweight and the other ones a bt underweight so i have to feed them two different foods. A low fat food and an ordinary dy food. And then the cat will eat everything, good as a dog

Participant7

The same with us I see the small one that's thinner of the two and she'll actually catch food and toss it in the air to make the other fella come for her. There'd be war

Participant8

That's the game

Participant7

The game is on, they start chasing each other around for this little bit of crust of bread. They just run around and around one teasing the other you know what I mean. They are great entertainment if you have time to sit down and just watch them

Participant5

Some wild birds, the robin now. We've a obin just after hatching, but they love the dog food as well, she comes to the door and takes it and goes off with it

Modulator

Does anyone feed ....

Participant3

I have mine on wet food cos she has cystitisand I did consider the possibility. We discussed it with *The vet* about putting her to sleepcos shes going to the tray, and she'd run out and destroy the carpet and timber and all that sot of stuff. So she was on AMATRIPOLINE so that was swelling her out, then she started to cough and she had oedema, but i got this wet food for her, I got it on the internet, it's natures way and I put cider vinegar through it with a lot of water and that has cured her touch wood so far so good

Modulator

And are the other...............?

Participant3

Other three are on dry food, Royal Ccacnine Yeah, but she is on the wet food, so I have increased I just keep increasing the cider vinegar all the time

Participant5

What does it do? What does the cider.....?

Participant3

It kills the bacteria in her bladder. Supposed to be

Participant5

And how does it taste then?

Participant3

Well in my innocence, the first day I cought her and opened her mouth and put it straight in. She took off around the house upstairs. But then I just got one teaspoon and put it throught her food, You'd know by her that it was sour and I just kept giving it to he, she started eating it, then two spoons the following week and now she is on four spoons in the morning and four in the evening and now she s back to herself again, hopefully it will last

Participant5

Thats great

Modulator

And do you feed them seperatley or ....?

Participant3

Yes i feed her in the kitchen and I feed the others up in their own bedroom, they have a bedroom for themselves

Modulator

Very good

Participant3

Ah but she is cute, she knows wee not to go upstairs, she knows where she is getting fed and knows the rattle of the bowl and the whole lot

Modulator

Great

Participant3

Yeah so

Modulator

And what about you Participant2

PARTICIPANT2

Ah all three of ours are on the royal Cnine light formula now, they have been on it about six months now

Participant9

And have they lost wieight

Participant2

And before they were getting Royal Canine Indoor cat fomula

Matin

Ok

Participant9

All Three were too fat

Participant2

Well they are getting smaller

Modulator

And were they getting bits on the side as well?

Participant2

Occasionally , yeah I mean there's cat grass things out there for them. Julius like the paste. Malt paste

Participant9

They like chicken

Participant2

They get chicken

Participant1

And hamburger and whatever they cn steal from you

Participant2 and if theres left over curry Julius will eat that Yeah

Modulator

Curry is good for cats

Participant2

I think he is an Indian cat but Julius will eat just about anything the other two not so much but

Participant1

Yeah thats it the other two are fussy, they don't eat everything

Participant2

Galford will eat cheese

Participant4

If it is too spicey they just won't eat it

Participant6 I have to stand beside my rottweiller otherwise he just won't eat, and the terrier takes his food

Participant9

The small one?

Participant6

The small one yyeah. She growls and he's gone out the door, and I'm there and it's like stop yuo know. Yeah and he's pretty big you know he's up to my hip and she's only....

Modulator

She’s the boss

Participant6

Yeah she’s the boss , typical terrier yeah

Participant1

Yeah a terrier does that

Participant6

I said I’d never get another terrier but she had the pups so I do n’t know

Modulator

Well Participant9 are you on big bags of food or........

Participant9

Yeah I have always fed my cats dried food, I was under the impression wet food is not good for them because of the sugar content. It’s what iI understood so I always gave them dry food and they used to be on the IAMS food but I made the mistake of free feeding them and one of them and the one I don’t have any more, she was fine, she just ate what she needed and go off but the other one would eat all around her. So she had to go on a diet. So she is now on the Royal Canine. She’s on the young male. She’s big boned and a big cat. She will always be a big cat . Like she is 5.2 or something. *The vet* was thats ok, fo her that weight is ok which is big enough for a female cat. She’s on the male food, she would have had to go on the obesity food for a while, the problem was because we had her on a cetain amunt she was allowed ti=o have every bit but we were not really sticking to it. Then I brought her into *The vet* t get her weighed and to get *The vet* to give me atalking to, which she did and I went home with a new regieme and with the obesity food. She is back on the male food now, but I live with two othere people so we had to do a thing on the calander, like mark that she has had her breakfast, she has had her dinner so that she is not getting fed by everyone. And I had to give someone I live with a real talking to because he’s the worst, giving her a little bit more and a little bit more. And now that she is back down to her own weight he’s gone back to giving her a little treat, and I said you can’t or you will have to pay the vets bills. She is fine now. We have got it into our heads that we have to becaus e of her health, just feed her the right amount, and I feel that is very important that you have to be responsibleIt’s in the animals best interest that you don’t give them too many treats. The other thing in theory about home made....................

I have read online people talking about cats should have a raw food diet, they should buy a chicken carcass in the butchers and mince them up which is interesting, biut I am not going to go that far

Matin

Right

Participant9

You know if *The vet* tells me the Royal Canine food is good for her then I ma happy with that, you know and she loves it I do worry about her getting bored with getting the same all the time but i think that is just me, humanising her

Modulator

Fair enough

Participant8

I don’t think cats mind, if they love something

Participant9

I don’t think they do No

Participant8

My cats eats an awful lot of tuna and just loves it. No matter what I give him when the tuna comes out he would nearly take the door off the fridge just to get at tuna. He loves it every time

Participant9

My cat is always interested in anything I am eating

Modulator

I think if you could bottle the essence of tuna you would make a fortune. What about you Participant4?

Participant4

Em well I’ve go so many on special diets

Modulator

Right OK

Participant4

But everyone knows where they have to goand they all go to the right places to get their food and the food is left down for the length of time it takes the oldest one to decide that she has had enough because she is 16 and she like her food to be moved from one spot on the floor to another and maybe up on the setee, She is the one that no one want to go near anyway, she doesn’t like anyone. So they

get their food down for up to half an hour and there is actually six of them in the one room that all have toe same food so they could actually go to each others dishes But everybody's fine except one who is, weight wise, it doesn't make any difference with her, she's always been active, she would eat all day, non stop if the food were there but I mean it is only put down twice a day , so the food is not there but it makes no difference to her body weight , she is just a little round lump. Theres another one there who eats the same and has not an ounce of fat on her, so obviously poor little Jenny wsa born to be rather large

And I have got two that have FIV ,and well they have to be kept separate because one of them thinks he is a dog and hates cats other wise i would love to have been able to pu the pair of them together but they obviousely can't with other cats but they both get their food put down left and they can ake it during the day if they want but they are both on CD diets anyway because of bladder stones and the dogs get fed twice a day, food put down , they will go their separate spot then they eat it check everybody elses dish to make sure nobody has left anything and we just take clean dishes up and that is over and done within a couple of minutes

Modulator

OK

Fair enough. Most of you use proprietory dog foods maybe add a little extra in. Do you mind telling me why? Do you think rather than going for the home made option as I say, about grinding down

Participant8

It's a little more convenient than buying chicken carcases and grinding them down

Modulator

Yeah

Participant8

Yeah ia little more convenient and also i buy it in the vets as well ans i trust her opinion on it. I think this has everything she needs You know Yeah

Modulator

What about scraps, would anybody be thinking about scraps?

Participant6

I put on extra rice and pasta and stuff like that. Well not every day but evey couple of days

Modulator

And would you feel that em.. would you even think off going to just scraps or why do ye all use complete dog foods

Participant8

Because it has what she needs, I wouldn't know what to give her. I woudn't go out and catch birds for her. The main reason is it has what she needs as far as I'm concerned and it is convenient

Participant2

It is convenient. The reason that I started on the Royal CAinine is that you could get it in 10K packetswhich only with two cats to start off withis a lot of cat food, itd last you a while, don't have to buy cat food every day. And also you can have it delivered. Zoo plus deliver all over Europe On line.. GOS turns up a week later with all your cat food. They might think you are odd buying 40 or 50 kg of cat food at a time but the bags don't go off for a year or so, throw them under the stairs so

Participant5

WHat was wrong years ago when there was no proprietary foods and there was potatoes mashed up and a drop of mil put on it and left down to the dog and they survivied. I think my dad wouldn't survive now. What was wrong with that? I don't know

Participant8

They must have been malnourished if they didn't get any proteins though

Participant5

Those words weren't used

Modulator

The oldest cat if I've got it right ,recorded died back in 1952 so that meant it was 34 I think so that meant it was born back in the 1930's and I don't think,.....

Participant6

Royal canine wouldn't have been around then

Participant4

An the cats name was MA, so I assume she wsan't neutered

Modulator

That's true

Participant3

I think it is very important to look at the meat content that they actually get. The one that I have that has cystitis she gets ovet 80% meat in it. Instead of Whiskas if you look at the back of the packet it's 4% meat or something

Participant7

It's interesting reading to see the the sugar contents thats in all the food today, when you were feeding ...., taking it from my own thing on the farm............... well there was bacon and there was cabbage and all sorts of things and potatoes and they were all just mashed up and given to the dog

We had german pointer for may years . That ws it. We had an old German Pointer in the house and he lived for years aand year snad years and years and he got so grey in the end and used to sit outside on a Wednesday and pick an apple tart up for himself. He was a pretty intelligent fella, he used to go and pick the apple out of back when the delivery man use d to do the drops. He used to sit outside , only on wednesday. As soon as the shutter would come uphe'd go up to shop. When he walked out he'd het his apple tart. He lived to a great old age and he got nothing only what came out of the house, big pots of spuds put on for them

Participant6

Yeah I had another hound and he only got scraps and he lived to be 16 years or maybe 17. We had him 16 year and when we got him he was fully grown, so he was probably about 17 years

Participant7

All scraps then

Participant6

Yeah

Participant7

I just wondered you know, the food that you are giving them today you know there'd be a lot more sugar content in the food today you know we all get more chocolate you know. There was no chocolate in our house growing up or sweet food like that

Participant5

I didn't realise chocolate was so deadly for dogs until recently

Modulator

Yeah absolutely

Participant5

And you have to have then pumped out if they got it

Modulator

Yeah

Participant6

It’s the same with raisins. Raisins can kill a dog. A handful of raisins killed a great dane. Thats the first time I heard of it

Participant5

And would they eat them if they were offered them

Participant6

I used to give my little terriers grapes and I read this about a great dane dying after eating a handful of them, so I stopped

Modulator

Wise move

Participant2

I don’t know if we cook enough at home to feed only scraps , i mean some of the scraps would look pretty nasty

Participant5

Yeas ago hee i think a saucepan of potatos were put down regardless of the number that were there to eat them. So there was always spuds left over to feed the animals. They were included in the dinner

Participant2

I think the other thing that influenced our choice when choosing the cat food earlier was the advertisement on the bag, with the indoor formula assuring that low stool odour, which when you live ina small flat in Germany it is one of the things............................

Modulator

So it actually wors then is what you are saying? And do you think it’s cheaper then to go that way especially if you are not making your own dinners and stuff like that

Participant2

I don't know if Royal Canine is the cheapest way to go, if there wasn't cat food at all I think cats would probably eat just as well, but I will say that the bought food does work

Participant1

It's also anti fur ball

Participant2

It's anti hair ball as well

Participant1

I am sure that that works becuse they still ...

Modulator

Coughing up bits

Participant8

It doesn't work with my cats , it makes him worse, Yeha . he started throwing up all ove the bedroom carpet, a new carpet, so I had to give him the anti hairball food. But about the chocolate for dog I always buy the chocolate drops especially for dogs because when you are watching TV on a Saturday and you/ve got your chocolate out ....

Participant7

Get a new carpet in the sitting room

Modulator

I'm going to bring in the coffee and let ye have a bit of coffee for a couple of minutes and if you just want to relax and it just might loosen up the ....#

Participant7

The interesting thing is, what I found is the experience I had when I was trying to buy the dog. Buy the dog do you know trying to find the maltese terriers I mean the conning thats going on out there

Modulator

Oh Yeah

Participant7 What people are doing out there In terms of order to sell dgs is absolutely criinla

Participant8

It shoule be seriousely regulated

Participant7

Absolutely

Child

We went too see a dog in Athlone Do you remember that Dad ? and they said were not allowed to go near their house. We had to meet them in a car park. So when we arrived in Athlone they nly told us on the way. And when we arrived in the carpark and they had a two week old that was meant to be 12 eweeeks old . They HAD pictures on the internet and it looked nothing like the dog, and they had like a wine box, and he was nearly flooding in his own pee

Participant7

And you werr Please Dad buy it for me don't go home I have to have that dog

Child

I wanted that dog

Participant7

You get your money out of the cash point and give it to the man and can have the dog. It's criminal stuff

TEA CUPS RATTLING

Participant7

The man said to me. I was under so much pressure to get this dog before Xmas

Participant8

Oh Before the Xmas

Participant7

He said he was going to Limerick and it was only 9 o'clock in the morning , the lights of limerick and he text me, Participant7 i found a lovely white fluffy replacement for ya, Yeah. But he said you'll have to swing by outer Mongolia on the way home. I'd been to numerous places looking at various pets and various dogs and what people were advertising and what when you got there, what you were getting was two different things, and the scamming thats going on . I mean this guy that turned up he had a

chip reader, for the little puppies and he gets paid. He has a chip reader i his car and give me the money and I mean these things are not cheap, they're about 700 euros for the little puppies

Participant8

&00 for a Maltese

Participant7

Yeah

Participant9

You should really breed them yourself

Participant7

No NO I wouldn't I have absolutely no interest in that atall

Participant5

Do they shed , The Maltese

Participant7

No NO

Participant5

They groom easily

Child

We brush tem every day

TEA GOING ON
Modulator talks about his training and lots of talking over ech other and cups and spoons rattling. Impossible to identify the conversation

Modulator

We'll all gather together again. We'll wrap up. We've only got two more questions to cover. So it's kind of I suppose following on on from the last bit of discussion. Just kind of looking at what influences your pets weight. I know that some of you had the battle with obesity adn I thin k we are nearly there. I think everyone asa pet owner has a battlewith diet and stuff like that. And I was wondering what you know, why some of ye had obese cats and dogs and stuff and why you thought they got obese. Or do you think it's just up to themselves. Or what way things go with them

Participant2

They ate too much and didn't exercise enough

Modulator

Fair enough

Participant1

And they don't go outside enough

Participant3

I think you have to be cruel to be lkind, jut give them their feed when they are supposed to get it and that's it

Participant1

Yeah

Participant3

Cos cats keep meowing and running after you when you have food

Participant4

Yeah they are always begging

It's a bit of genetics involved, we had he was 17 when he died the cat , he would eat non stop, he also expected to have his cornflakes with milk every morning which he was about 13 when we first got him, he had to carry on doing that, but he would eat everything and there wasn't an ounce on him> I mean he should have been massive. And there was no weight on him at all. Like I said there's another one that doesn't eat that much at all and she's fat. No polite way of putting it. She is fat

Participant5 else going to grab it when she is gone. SO I'd ssay that is probably an influence as well

I think if you have one animal as well they don't have to watch for somebody else, that dog might come and eat it unless I eat it. You know. So at least with my dog I think she'll eat so much and leave the rest, she knows. I'd say there is nobody else going to grab it when she is gone. SO I'd ssay that is probably an influence as well Tha, that they are the only one having the food

Participant3

Theres harnesses you can get for that cat. I often thought of walking them , but I couldn't when I don't want to let them out you know.I keep thinking that when they are into the habit they will keep wanting to go out, but i just gave that up as a bad idea

Participant2

Participant4 tried that for a bit, put the cat in a hanes and tried walikign it

Participant1

It didn’t work out

I did have two cats that every time they came back form the vets . Cos i did start that with them, when they were . young, and when they started to get into their teens and needing to go to the vets regularly Don’t ask me how I knew what they wanted but they virtually demanded that they were taken out and walked all the way around the house in their harness. Out where I ive I am sure there are a few people that think,that woman walking a cat

Participant8

I’d love to wlk my cats but I guess I’m afraid what people would think

Mureen

I think I’d have to do it when ...

Participant8

In the dark

Participant4

Sort of younger

Participant3

I’d be afraid that a dog would chase you, run after you

Participant7

Tell them a hoody comes with a lead

Modulator

Do you think walking cats is a bad idea then ?

Participant3

Absolutely, especially when you don’t let them out you know, so they’re indoors all the time

Participant5

Did any of you see the girl who wlks the ferret in Salt Hill Did you ever see that?

ALL
YEAH YEAH YEAH

Modulator

That's almost acceptable though

Participant5

The first time I saw now I took two jumps I was full sure it was a rat . The I heard it wa a ferret, I don't know why. The fact that I heard it was a ferret I was more acceptable, They are supposed to be loveable pets aren't they?

Participant1

Yeah I heard that too

Modulator

So what abouot even toys for your pet ? Would you think that helps or does it make any difference at all?

Participant9

The can opener . Teasing. We kind of, no really an exercise regieme, but we sometime do exercises with anything long , just oulling around the palce and getting her to rum and jump after it. One time actually my house mate and me were palying with her so much that she got out of breath and started panting. I panicked because I had never seen her like that before. Looked it up online. *The vet* was saying yeah well that can happen it is ok, but I was a bit shocked when I saw it. But that was ony one time so far that has happened.. Kind of yeah get her moving a lot. You know she goes outside, my cat goes out and goes off and does god knows what. She could very wel be going out and sitting somewhere I don't know if she's getting any exercise when she goes outside. So the only exercise I know she gets is when we play with hher like that

And throwing things, actually my ct is a bit like a dog , I don't know if anyone has cats like that. She gets things she likes, it can be a bottle top or string or things like that and she wil bring them in and put them down in front of you and meow at you. Bottle tops particularly, she likes to have those thrown, but she hasn't really mastered the thing of bringing it back. So she likes to hve it thrown and then she'll run after it and then meow at you again so yuou have to go and get it. So it is exercise for me as well, Yeah so that is her exercise

Modulator

So anything else influencing your pets wieights? Glad to see none of you have said being neutered

Participant9

I suppose it is isn't it

Participant3

I don't find that a problem really

Participant9

Irresponsibel house mates

Modulator

Fair enough

Participant6

Neutering keeps down the weight

Modulator

Well I mean they say it stops or reduces their metabolism, it is interesting to see that nobody has actually said that because their pet was neutered that is why they got fat. Not saying that neutering causes obesity just that some people do say to protect themselves in a way

Participant2

Surely it is attributed to lack of exercise and over feeding

Participant9

Over feeding has got to be the reseon really hasn't it

Modulator

Yeah Yeah

Participant4

I knw I have to watch my husband as far as the dgos are concernedcos it's k with the cats cos like I said there is too many dies he daren't feed cats. But i did end up when I had just one little dog I got reducing diet from the vets and put it into an ordinary bag so that when he was overfeeding her he wasn't putting any more weight on her. I know it's awful

Modulator

Participant8, you were going to say something there

Participant8

Oh I can't remember, about the neutering was it? Or being overweight the cats and dogs. I blame the other dog for not eating, so the dog is overweight from stealing the food, it's not exercise either it's in her nature, she is big boned and loose skinned and the other dog is the opposite. I think some dogs it is in theirnature and some cats> Like you were saying it is very difficult to control weight, you can feeed them the exact same and one will put on weight and one will loose weight

Participant9

Still though when you've two, when I first put my cat on a diet we still had two cats so i had to stand over them while they were eating their own food and not eachothers. I only have one now.

Participant8

And you forget, you go off and you do somethingand they are so smart you know, you turn around at it is gone, and you know it wasn't the finicky little eater who eats only one little bit at a time and it's gone in five seconds

Participant7e

When you neuter. Tom cats especially, you know male cats. That stops them rambling doesn't it? So I mean if they weren't neutered they'd be picking a lot more. I just noticed when we nutered the little black cat, both of the cats we neutered were both strays, but I mean he got huge from being around the house all the time, where as the big ginger cat would ususlaly come we would feed him, he would build himself up and come back slim. It would be interesting to see if the black cat had not been neutered ifhe would have taken on that size that he took because he would have rambled more

Participant1

He would have missed meals

Participant3

I find as well, the one that has the cystitis, I normally wouldn't allow them up on the counters, They're very intelligent. If someone came into your house and youhad four cats walking around on the counter they wouldn't be inclined to come back again. But I find that since she 'sa allowed to jump on the counter whenever she wants to and with her changed diet she is a completely change cat . She's become more lovelable and the others know they shouldn't be up there . If I came out of the room and they're up on the counter they are gone down straight away, Ya know strange

Modulator

Very good, has anyone else anything to add to that? What they think about over feeding and lack of exercise

Participant2

Does water intake count?

Modulator

Which? Sorry?

Participant2

Water.. One of the things when people try to loose weight is you need to drink more water, we got one of those drink wells for the lobby. I think the cats do drink a lot more now that we have it

Participant8

Really! Beacuse it is flowing or.........

Participant2

Whetther they like the noise or who knows why but we refill it al least once a day now, where as we changed the water once a day but they usually wouldn't drink or empty the bowl But now ..

Modulator

They've lost weight you think?

Participant2

But we have also had them on light food, so it is hard to say whether it is the extra water intake or it's the light food

Participant1

They did though in over half a year

Participant2

But I got the drink well at the same time, that came in the same ZOO plus order, thats the first dried bag of lite food so it's ah.

Participant9

No, definitely regulating her food or diet was what worked for us cos we weren't really keeping to the diet properly and then we gave ourselves a good talking to and sticking to it ridgidlyand whe she went in in six weeks time she was weighed and was down to her target weight. I was even worried she had lost too much, so it was definitely when we copped on and started doing the right thing. Then she was fine, so it was our responsibility really

Modulator

I suppose the last one. It's about exercise routine, and I suppose it is more aimed at dogs but it does apply to cats as well. It's kind of like what you were talking about earlier Participant5, about taking your

dog out for a walk and meeting other people and stuff. What are your ideas on that Has anybody got anything to add to it?

Participant5

Yeah, you know even when they are puppies they'll bark at every other dog in the beginning and now she can walk and ignore them, you know even walking along the promnow it's much easier that she has got older to walk along the prom and meet other dogs

Participant7

I'm afraid I'm not one for leads you know. I don't like dogs on leads . So I just, there is a place behind Fugo and walk I walk down along the shore, I head down there , head up around the bogs about 10 o'clock at night you knowand bing them back black. Get myself into trouble. It's safer at back of Furgo and walk down along the shore

Participant5

Are you not nervous now if you see big dogs down there as well

Child

No

Participant5

If I go down to the pier n Location 1and see the big dogs.

Participant7

I would be quite sure about where I am going you know.what I mean. I mean I'd be very mindfull of the fact that, you know a big dog, a rottweiller over there , basically might see mine as two little rabbits and my biggest problem is not the Rottweiler. It's will i get home again, I'll have to get them home you know, if something happens to the dogs I might as well....

Participant4

One of our three dogs, when they are out for a walk. Well they are drawn towars the beach and then they get out of the car and they're running on their own, sort of thing, normally it's with my husband following them. So although he might overfeed them, he does take them out for walks, but you know they are the same as yours because there is nothing there that they'd be worried about and obviously other dogs in the area are dogs they'd see normally

Participant7

Yeah Yeah like that now I only bring them to a safe area, it's a.... I know exactly where I'm going and I know they are not going to meet any dogs or such. It's just we head down along the shore and away they go and everybodys safe and I know I can pick them up and bring them home safe again.

You know But as far as walking them on a lead it is a no no. I just couldn't do it. I feel it's.. again you know it's part of growing up on a farm, you know walking with dogs and leads and sstuff. I feel they should have a lot of freedom, i mean I wouldn't mind picking up dog and putting it in a rucksack, something like that if it got tired, but the point of carrying it around on a lead No I just think it is cruel

Maureeen

If I have to take my dogs anywhere, you know into town or anything like that, the obviousely I have to put them on a lead

Participant7

Oh Yeah

Participant3

I find with the cats they get their own exercise cos they chase one another. You know they are tearing up and down the stairs, in the bathroom and wine glasses being broken and all sorts of stuff but they get their own exercise themselves

Participant9

Yeah they have that kind of crazy half hour when they just go mad for no reason

Kevi n

Yeah they do

Participant9

Like I was saying before about playing with my cat, and I do , do it kind of for exercise, you know maybe to help with the weight but also just to entertain her, you know and i suppose it's bonding for us as well. To entertain her actually it's part of the reason why I do it

Participant5

They're beginning now to allow dogs into hospitals are they? For Therapy?

Modulator

Ah yeah theres lots yeah

Participant5

Even the fact that you're rubbing a dog you're not conscious of it, You know I thinkgit is good for the person to be rubbing a dog

Participant9

Yeah I agree definatley

Participant5

So I think they are stating now as well in the hospice

Modulator

So you find that walking your dog as well is helpful for yourself

Participant5

Oh Yeah Yeah I should walk him more

Participant8

When I have them out, I live in town. Every evening I have to, the dogs give me a choice but they love it. Once a week I'll bring them someplace, put them in the car, because I'd like to let them off, but they'd lwasy be on a lead. I'd be terrified of them being hit by a car. And they're safer

Participant7

Oh I agree with you totally. I mean if you are going to bing them to the pom they'd have to get a lead, I'm not one of those people, sort of set up for that as a person. I just prefer to bring them to a safe place and let them go, you know

Participant8

Well it's much easier what you're doing because walking on the prom anything can happen. They decide to bark at a dog or another dog decides to bark at them

Participant7

I find that stressful! You know what I mean, another dog coming or whatever, I just think Oh this is murder.You know I wouldn't enjoy myself as well. It seems much easier to head back west to just off Boffin hike back on the shore

Participant5

I have been out ther a few times

Child

Our two dogs have two different leads and they are always getting tangled up and you're falling over them when you are walking them, so recently my uncle gave me a dual lead and you just hold the one lead so it attaches to both of them and I think it really, really works

Participant5

A harness, you know a harness? They seem better don't they? Isn't it cruel idea to think that you are pulling adog by the neck

Modulator And ALL

Yeah

Participant5

At least the harness is a little more comfortable

Participant8

Be worse if the dog was pulling you by the neck

Modulator

Handy for the cats as well

So what about you Participant6? Are you?

Participant6

I live in the city so I have to walk. If I go to the park now if nobody is about I let the two of them off, but I get haunted even for having the Rottweiler on the lead, everything even people who own dogs they come up out of the way giving out to me, that i shouldn't have him, he's too big. But he is the most gentle giant, it is usually their dogs coming over barkingat me or something you know. Mine are very good, they're very quiet. You wouldn't know there was a dog in the house or anything. But if I go out to the countryside, I'm from carraroe, so they are off wandering all he time. Go to the beach let them ago all they neeed. But if you see kids or anything you kind of have to, the parents will give out to you, the dogs won't go near them, they won't even go over to say hello, you know they stay around but I suppose I don't want to get given out to

Modulator

So do you find walking the dogs stressful or.....

Participant6

No NO

Wehn you go out with my two dogs both stay, but when you go out first it's kind of stressful because the little terrier, ever since she was a pup, bite my legs you know she just gets really excited and she only does it to me . When she gts outside the door she goes in to play stance and then bites and even the rottweiller he's kind of looking at her and hitting her, tying to protect me from being bitten I suppose. The first five minutes , Once i get out of my estate she just sits back you'd hardly know she was ther , dragging me, well she is so small she doesn't drag but she will try to pull me down the raod but then after that she just stand there, the two of them just stand there they are pretty good

Modulator

So do you think you walk more? Would you do the same amount of exercise if you didn't have the dogs?

Participant6

No definitely not, thats one of the reasons why I got the dogs, one was I was minding dogs and had to get my own then in the end, but to get out of the house in the evenings cos otherwise I'd come home fter work, cook the dinner and just sit ther then. At least I do get out for half an hour or an hour every eveing

Participant8

I agree with that, I'd stay on the couch for the night if I didn't get up to walk the dogs

Modulator

So do you think you are healtheier as well as the dogs being healthier because of it ?

Participant8

Oh definitely yeah, because one of my dogs had an operation thee weeks ago and I hav't been able to walk her since, well another couple of weeks and I will nad I definaltely say she has put on a kilo or two so have I you knowso I am really looking forward to getting her better

Modulator

And what about the c ats? Do any of the people who just have cats? Do you spend or do you have a particular exercise regeime or spend a bit of time palying with the cats or anything like that?

Participant3

One of my cats loves playing with balls, small balls, so I get it at the end of the stairs and throw it up, she chases and brings it back down

Participant9

She'll bring it back down

Participant3

Yeah she goes sround the house with the ball in her mouth and all that sort of stuff

Modulator

And would you do that kind of religipusly or just kinda whenever the....?

Kevein

Whenever I am i the hallway and I see a ball I'll just throw it up

Modulator

Thats fair enough Yeha

Pasty

Tenis balls are great for that

Participant8

Yeah Yeah

Cat nip

Participant6

Me brother has a cat, he showed her to me. He's liveing in amwerica. I've never seen it over here, cats go crazy for it

Participant3

I spray it on the scratcher

Participant5

Did any of you ever have to leave only just had to do that

I have the dog eight years, it's the first time cos there was always a family member yo a dog or a cat to be minded by someone else . Well I have to do that now. I have the dog now eight years asn it's the irst time cos there was always a family member you know that'd take the dog. My dog has a couple of homes where she is happy, but this last time we were at a family wedding and every body was going you see and I had to have the dog minded for the weekend and when she came back her voice was gone she couldn't bark. Seemingly she was with a very good woman, I have no problem with her at all, she just cried all the time. Day and Night

Participant6

My rotty loses weightif ever I go away. I was down before for three months and my family had him and then some friends had him, twas all people he knew and used to and everything but he just would not eat. I came home and I could see all his ribs here and his back bones and everything. Last Christmas I went to America for a month and I came home and the same thing, he'd lost loads of weight

Participant5

He doesn't eat No?

Participant6

He doesn't eat he just kinda pines for me

Participant5

Thats what mine did, she just didn't eat and she cried, she had no voice and she couldn't bark, she was just making the whole thing an awful thing to come back to, to see your dog upset, you know and ther wasn't a thing we could do

Participant8

Would you try a pet sitter? Someone who come into your house and feed the dog and walks it twice a day

Participant5

But the dog has never been on her own. The most my dog is on her own is about an hour, if I go into town or maybe an hour and a half. There's always somebody coming or going and this is the first time for a family wedding. We were all gone

Participant6

You should bring the dog with you

Participant5

I wanted to see if there wa as pet friendly hotel, and I live a hundred yards from here. Where is the pet friendly hotel? The twelve bar Co Galway and I asked the manager how does a hotel become a pet friendly one? And they have leather fittings and wooden floors and everything and you have the dog or whatever it is in the room with you

Participant6

Some of them have a minder

Participant5

There's only about two hotels in the country that do that you know

Participant6

Theres minders in the hotel isn't there, they sit with your cat or your dog

Participant5

But sure that is no good to me when I am only up .you know

LOTS OF LAUGHUNG AND ILLEGIBLE TALKING

Participant8

The B and B guide lists some of the dog friendly B and B around the country That would help

Participant6

Theres not very namy in Ireland though, theres loads in Enfgand, I don't know if it's the same book now, it's the AA1 or something but there is like about five in Ireland

Participant8

Yeah

Participant6

That was the book I had, now was about two years ago

Modulator

I don't we have got into the whole pet friendly thing in Ireland yet

Participant5

Oh No

Participant6

I noticed there wasn't many like I don't know

Participant5

I strolled her into a hotel once that's all....

Participant6

....

Yeah I suppose thats true yeah People are admitting to a lot more and it does tend to make my research a bit easier

Participant7

It’s interesting about your research, have you found a different culture between the people in the country and to that of in the city

Modulator

Not yet, this is the first one, this is the first focus group. As far as owning pets people are far more likely to own pets when they’re living in the country. When they’re in a rural setting and I suppose it the sheer physical nature of owning a dog or cat where you need a lot of room for the dog to roam and especially for dogs . But even for cats if you’re in the country you’re more likely to own a pet. But other than that I’m hoping that this kind of stuff will give me a little bit more insite into it but there is definitely some differences Yeah, but I do think what seems to be and actually shows up today as well. What seems to come through a lot is that if you have pets as a child you will have pets as an adult as well. It seems to be that kind of follow through. Our own life learning experiences kind of influence whether we have pets or not, You all kind of said that today too, that because you had a pet as a child that was the reason you had one now

Participant2

Could that be because you had a pet as a child you maybe most likely have an response to it as an adult

Modulator

Oh That is such a mine field , as part of my research I did some work with asthma and pet ownership and that was one of the things they looked at, allergen exposure as a child. So if you were exposed to dog allergen as a child you are more likely t o be allergic to a dog , however if you were exposed to cat allergen you were less likely to be allergic to a cat. And then they found if you were exposed to cat allergern you were les likely to be allergic to a dog! It just goes on and on and they don’t really know for sure and a lot of it seems to be locas as well so all the research will be done in say Germany, a toen in Germany or a city in America or we’ll do it in a rural area i Russia and every single one of them seem to come up with different things and there seems to be a lot of local reasons for it. And probably a lot of it as well has to do with genetics. So it is very difficult to say for certain if your’e exposed to it, you know if the dog is outside and you’re only getting small amounts of exposure you might be less likely to be, you know that kind of stuff, they don’t really know for certain unfortunately. But i think it is a lot of learned experience as well that it is in your mind, Oh itis nice to have a cat and you are used to that as well. Whereas if you have never had a dog it’s kinda like, oh will I go get a dog, you know they seem like a crazy little thing to have around, you know that dog s are a big commitment and it’s about committing, you were just talking about going away and you have a dog or a ct and you are living in a different country from home and you can’t rely on your family to get them

Participant2

You board them

Modulator

Yeah I know Yeah, well that is what you have to do and you have to make sure thay are looked after while you are away, it's not like you can just go away and leave them in the house but they definitely are a little bit.......

Participant5

But as a child as wellwe'll say we had dogs as achild but you never used the word pet, they wee part of the house

Modulator

That's right Yeah

Participant5

They were part of the family, but they weren't considered a pet as such, sure they weren't. I don't remember using the word pet with our dog as a child

Participant6

More Like Mutt

Participant5

It was just the dog or the dog had a name and that was it, they were part of the household, they fell into line, you know

Participant7

It varies with the function

Participant5

There were more sheep dogs then

Participant7

Yeah I mean yu had the terrier out in the country with the terriers chasing rabbits and all that kind of stuff, it's just the typw of culture you know. When I was younger we had terriers and all sorts of things

Participant5

They got rid of mice and everything

Participant7

A lot of farmers would have a terrier for that very purpose, maybe all the animals around the farm would have had a function and as yousay the word pet was not used very much

Participant5

The word pet wasn't used as such

Participant7e

You'd get a cat orr a dog and usually you got it off a farmer locally, he'd have a dog, a good sheep dog or a good gun dog and yer man up the road here is after having pups and you get them and bring them back in, there was no such thing as monsy exchanged. You might bring a bottle of whiskey over with you or give them something for the animla rather than paying extortional amounts of money for different breeds of dogs

Modulator

I'm going to wrap it up at this stage. Thank you all so very very much, It has been a very interesting evening. And hopefully now all the rest of them are going to be as talkative and get as much information it'd be great

What I am going to do is, I mean as part of my esearch, I'll be producing, hopefully, touch wood, all going well a scientific paper, the plan would be to send everyone a copy of the scientific paper once it has been published so you can have a look at wht you were involved in. I'll send you the whole thing but if you don't feellike eading through the whole lot the abstract of the sounds will give you a summation of everything, But I'll send you the whole lot so you can pick and choose what you want to read, you might even recognise yourselves, your names will be different, because we do tend to use quotations as wellas the analysis of the data itself

What I am going to do is, it is up to yourselves, if you don't want to receive any information thats fine

If you wouldn't mind passing these around and just put your name and maybe an email address, I mean if you want me to post it o you either but I'll need your postal address if you want that, the other thing as well if you would indicate whether you'd prefer to get a voucher or whether we'll just credit your account with the money, right

Sorry you weren't there , theres a little bit of incentive for being here tonight. Basically I've an agreement with *The vet* and what we are going to do is we are going to give you a €40 voucher or a credit note on the account, whichever you would prefer

You can start off Participant7

Participant7

It's like writing a school report

END

Location 2

MODULATOR:

So who's bubbling with ideas & who wants to talk about why they have a pets to start with?

Does anyone want to jump in there?

PARTICIPANT 1:

Well I suppose for company, because I like animals. I was brought up with them. I do tend to rescue them. I like to rescue animals rather than seek them out. I suppose it's, I take them on because if I'm presented with an animal, I just take it in. That's why I have too many & that worries me because, you know, I don't have masses of them but sometimes I do feel that I can't cope properly with the ones I do have. They obviously have a reasonable life & a better life than they would have had, but...

PARTICIPANT 2:

How many do you have?

PARTICIPANT 1:

At the moment I've got three dogs & four cats, but it's only me who looks after them really & the dogs in particular could do with more walking, but I can't manage that myself, but at least they are safe. But I would like to look after them a bit better, but that's something... What's the alternative? But I also wanted to bring up my children to have animals.

PARTICIPANT 3:

I think that's extremely important. That's a very important part of a child's learning experience, to have animals. A good experience, I think, would be generally at some stage in a child's life a pet is going to die & it's a very good way for them to learn the grieving process. I've had animals die & I've been very very lucky that both my dogs have lived very long lives, but when they died, it's like that one of the family members had died, but it's not actually the same because it's not a human being, like a parent or grand parent, but it's a great way for a child to learn about the grieving process & to learn responsibility.

PARTICIPANT 1:

Particularly if the animal has been ill & you mite have a young animal that's maybe being treated & then still dies, you know. That's part of the mourning process as well.

PARTICIPANT 3:

Absolutely, & I think that the other reason that we have pets at home is, like you say, that I've been brought up with them. I remember a period of my life when I was quite young, we were living in rented accommodation & for several years I couldn't have any animals & I found that really a dreadful thing as a child & when I became an adult & had my own home, the first thing I did was, most people would be down the second hand shop buying furniture, I was down the rescue centre trying to bring home a dog. They're a huge part of our lives. They give you unconditional love. I think that's a wonderful thing. Especially rescue animals, & I've had experience of both, but to see the way hey come around when they're given love & attention, it's just unbelievable. Dogs in particular, I have cats also, but dogs can come from something that's afraid of it's own shadow to being a family member & a friend, & to get confidence & it's just because you give them some love & attention. They would be the reasons why we have them.

PARTICIPANT 3:

I kind of agree,as I was about to say the same things as you Participant 1. In the beginning you know I was reared with animals. We'd everything from canaries, guinea pigs, hamsters, dogs, cats. Everything you know my pet mice, I've had a few of them. As an adult I kind of realised I missed animals in my life

when I didn't have them for a while & therefore I would always like to have other animals living in the house with me.

I agree with all the previous speakers have said about affection & that, but I also find it really interesting to have creatures from a different species, that think in a different way & act & behave in a different way, you know. There's a kind of challenge in that as well, to see them interacting with one another & interacting with us & to kind of know that you don't necessarily know exactly what is going on in their minds, you know. One of the girls at work had a goat that was rescued from the National Park, for a while & it used to come to work everyday with her. Yeah, a goat! Which was nick named Saco, after the rifle that missed it!

LAUGHING GOING ON HERE

PARTICIPANT 4:

A goat?

PARTICIPANT 3:

And looking at a goats eyes, you know, they're so different to human eyes & it's looking back at you & you don't know what's in the creatures mind, you know. So there's that additional challenge in a pet.

PARTICIPANT 1:

We had this lizard bearded dragon/ which I wouldn't have chosen, because I don't think you should have them, but again it kind of tuned up & he's died now, but I can still see him looking at me & it's totally different from a dog or a cat.

PARTICIPANT 3:

I think you're probably totally right about those types of animals because dogs & cats we understand an awful lot about them & they interact with humans because they've been with us for so long. So it's easy to understand them to a certain degree,what they might think, but with other animals it must be a real challenge.

PARTICIPANT 3:

I suppose one of the other thing's is, it's not quite pets. But, as part of my work, I would also have rehabilitated various wild animals you know, & my area of speciality would be bats. So over the last ten years I would have had quite a lot of injured bats that I would have minded for a while, & the very first one of them, again, she tamed really quickly. I had her for a period of six weeks, which was actually quite long term care for that animal & it's that thing that they're mammals & they're so like us in some ways & yet so different & need different kind of care. But that particular bat got to know me & would squeak in the evenings when I'd come to feed it. A kind of a certain fascination with animals.

PARTICIPANT 2:

Well I have two dogs & I think like the rest of you. They're rescue dogs & I originally had to, some years ago, more than ten years ago. They just walked into my life & in the garden or into the place I was working, so I took them home. But I had always had animals & just a dog really, all my life. I always had a dog & like yourselves you know there was a certain time of my life I didn't have any animals & then when I got the house & everything & the dog arrived , I thought well this is the time. And then I got a second dog, but then my husband & I were moved to India for a few years as part of his job, so we took the dogs with us. Now we don't have children so, like people say, oh they're your substitute children, kind of thing, but it's a bit more complicated than that. They're not just substitute children. It's not like that, but we brought the two dogs over. The two Irish dogs, & while we were over there we picked up two Indian dogs as well.

Now you probably know in India there's strays everywhere. The streets are..... so I could have picked up as many...... but I didn't, I supported some rescue charities there & then the only reason I picked up these two dogs is because people had pointed them out to me. I was in a car one day & a guy said look at the baby. I said Oh my God, there's a baby on the dual carriage way. It wasn't, he had mixed up the words. He meant puppy, so once he had said that, I'd felt the responsibility. I can't drive past this,so I got

him to turn around the car & we picked up this puppy & that was one. And again a friend said to me, look at the poor puppy. He was dying in the middle of this paving slab, so I picked him up & brought him home. So then I had four. One of them died over there. One of the Irish dogs. So we brought three back with us. I won't even tell you how expensive it was to bring them back.

PARTICIPANT 1:

I can imagine.

PARTICIPANT 2:

You can't leave them once you have them. This is something that you can't. People have said to me, you know, you can leave them with us, but I wouldn't have trusted them with anybody. But we brought them back & we ended up living in Spain for a year because of them. We didn't want to put them in to quarantine.

MODULATOR:

Oh right.

PARTICIPANT 2:

So we lived in Spain for a year. A very nice year. So we brought the three of them back & one of then, he's since died, & now I have my two Indian dogs. So they've had quite a life. So that's my story.

PARTICIPANT 3:

So how old are they now?

PARTICIPANT 2:

They're quite old. One of them is actually dying. She got cancer. She's about ten, just over ten, & for an Indian dog I think that's probably pretty old because genetically, I wouldn't say they're built for longevity. They're built street dogs.

PARTICIPANT 3:

What sort of breed are they?

PARTICIPANT 2:

One is like an over weight greyhound or a whippet. Very over weight., & the other maybe looks like a small version of maybe a Husky. Stand up ears, very white but small. Maybe not as fancy as a husky.....

PARTICIPANT 3:

That's probably quite remarkable then, because as you say even with given any kind of care, where they came from, they're life expectancy was probably very very short.

PARTICIPANT 2:

I would think so.

PARTICIPANT 3:

Obviously because......

PARTICIPANT 2:

You see they're built for survival. Especially the females. I can't see them lasting more than a couple of years on the streets.

PARTICIPANT 3:

Litters of puppies &.....

PARTICIPANT 1:

It's like foxes, a asimilar animal & they naturally only live about eighteen months, I mean in the wild. I believe that's they're average sort of expectancy.

MODULATOR:

A lot of them die as Neo Nates though,as they get older, they live longer, if you know what I mean.

PARTICIPANT 1:

So that's about the average yeah?

MODULATOR:

So what about you PARTICIPANT 4?

PARTICIPANT 4:

My story is not anything so glamorous as that lady's. As a child I always had pets. Rabbits, hens, chickens, a dog & that kind of thing & as one does then, you spread your wings & get married & some years after that we eventually got an Old English Sheepdog, a bitch, & we had a couple of kids. She was absolutely fantastic & brought an awful lot out of the kids.

You know you get interested in them & you show them a bit & that kind of thing. I had to put her to sleep. I was without a dog for about seven years & there at Christmas they presented me with this Labrador. That's seven years ago & we got totally hooked on this over weight Lab, but she's a fascinating dog & I've done a lot of things.

I've a boat on the Shannon. We've gone from top to bottom several times & I trained her to retrieve people from the river. I saw a lady on television many years ago with a Saint Bernard, of all things, in a kayak! & she had the Saint Bernard trained to go in & rescue people. The beauty of the training was that the Saint Bernard could jump into the water without turning the kayak over. Fascinating.

I got working with Honey & I'd throw in the life ring & eventually started working with young fellas in wet suits, & then went up to adults. Heavy men & she was quite able to bring them in you know. And I actually had the pleasure to see her rescuing a German man in the Shannon, in Terryglass, one morning. He fell into the river. I threw in the life ring, instinctively just threw it in & before I knew where I was Honey was gone down & in.

It was nice yeah. He took us up for lunch & after I got a lovely letter & photographs from him, you know. Which was nice.

But she intrigues me with the intelligence she has. Frightens me in fact & I think one of the best kicks I get out of her is, you know the way kids can be fearful of dogs & on three occasion now where kids would shy away from the dog & get behind the mums apron or whatever. I find the dog I have is very very good for calming the child down & losing the fear of dogs in a child. I've done it three times now & it really works very very well you know. I'd love to do it again, well if the opportunity presents itself.

We took her off on holidays, herself & myself, camping the year before last. Right down through Europe. We did ten countries, 8000 km & home & we're heading off on the 16th of August again.

PARTICIPANT 1:

Where you going this time?

PARTICIPANT 4:

Well we're heading off to France anyway & we'll make our mind up then.

She's older now, she's eleven & I don't think, if I get another year out of her I'd say it'd be as much as I would do.

A chap called to the workshop the other day & he was telling me he has a sixteen year old Lab & I didn't think they'd live....

PARTICIPANT 1:

I had a Collie that lived to seventeen.

PARTICIPANT 3:

I did too. A Collie to nearly sixteen & a half.

PARTICIPANT 4:

Seventeen years old. I didn't think they'd live...

Yeah, but that's about the size of it.......

She frightens me sometimes with her intelligence. Really does.

MODULATOR:

So really what your saying is, you didn't actually get the dog at all. It was the kids got it for you?

PARTICIPANT 4:

Well yeah, honest to God yeah.

PARTICIPANT 3:

It's funny, like you, I'm a real advocate for don't buy dogs for Christmas. Don't get dogs from breeders. Take them from rescue homes & centres.

I got a present for Christmas also & I was always ashamed to say it. I was always the one saying, you don't by a dog, you go down to Paul's or go down to the Ambrose foundation & get your dog from there & give them your money. I ended up with a dog, she's a Boxer, I think she cost 450 Euros.

PARTICIPANT 2:

She doesn't know though.

PARTICIPANT 3:

Not at all..... & if she were to go to a Boxer club or Krufts, they probably wouldn't even let her in the door. She's the worst example of a Boxer. Probably the runt of the litter.

PARTICIPANT 2:

Does she have a tail?

PARTICIPANT 3:

Unfortunately, no, it's docked & badly docked. It took me about a year before I could find a vet that could sort it out for her & lucky I did. Thankfully she's fine now. She's just really a wonderful dog. Like your story, wonderful with children. I've two Boxers & they're both wonderful with children, but they certainly wouldn't ever win an award. You'd never show them or anything, but like the other one was a rescue & I love the two of them. I didn't want Boxers in particular, but that's what I required, so that's what I have.

PARTICIPANT 4:

I always thought Boxers were placid. I was out walking the dog one night &.......

PARTICIPANT 3:

They're very hyper.....

PARTICIPANT 4:

And eh & the two of them attacked my dog, the Lab...

PARTICIPANT 1:

Your dog?.....Is this recently?

PARTICIPANT 4:

It's about three & a half years ago.

PARTICIPANT 1:

Because my daughter's been walking by the lake & there's been Boxers.

PARTICIPANT 4:

Well they were intent on doing her in you know...... its the only time I ever really kicked a dog in my life. I hated myself for doing it, but I had to do it. They had her by the neck & they were down &.....

PARTICIPANT 3:

That's horrible to hear because they're exuberant dogs.

PARTICIPANT 4:

I thought it was unusual for Boxers.

PARTICIPANT 3:

They run up to dogs, they run up to people & it's will you play with me. Especially little dogs you know if they even look at them they're gone. They're terrified, but they are a boisterous dog. They will even with training, it's difficult stop them from jumping up on people & that type of thing, but I've never heard of

them being..... mine have never been in a fight of any description. They've gone up to dogs & they either run away in fear or the other dog has been taken away.

PARTICIPANT 4:

I had a Boxer myself for years.

MODULATOR:

They are bouncy.

I suppose I'd say, you kind of mostly all have rescue animals, but the idea of the question I suppose is.... why dogs? Why cats? Or if you don't have cats, why not cats? What made you choose dogs instead of cats? Or what made you choose cats instead of dogs? You know that's the idea of that question. Why would you choose that particular animal?

PARTICIPANT 3:

I suppose people choose dogs, & as a pet a dog or cat is going to be in the house with you. You know if you have a horse or a goat it's not going to be in the kitchen. So, that's why people I suppose choose dogs & cats. I have a cat also. I had two other cats which unfortunately as cats do went missing on me. I suppose that would be my reason. I've had rabbits & guinea pigs & all that type of thing when the children were younger but the main idea is that they're part of the family. The cat or dog is in the house with you. They go on holidays with you if you can bring them. They go for walks with you & they go in the car with you. Where you're not going to put a rabbit in the car with you.

PARTICIPANT 2:

Well you can.......

PARTICIPANT 3:

Yeah, but generally you don't put a rabbit on a lead & tie it up outside the shop to wait for you. So, I suppose that's why people choose dogs & cats.

PARTICIPANT 1:

We had gerbils & rabbits & guinea pigs & a rat & all those different things when the children, you know, for the children when they were small, but it was always me who ended up looking after them.

PARTICIPANT 3:

That unfortunately is the problem.

PARTICIPANT 1:

I mean, one of our cats is my daughter's which she brought from England as a kitten & I do the looking after mainly. My son had a cat, well it was his ex girlfriends. The girlfriend went & the cat stayed, along with the lizard & one of the dogs was found on my daughter's boyfriend's farm. She wanted to take it & look after it. But it's not, it's me. They always did have them when they were younger to look after so....

PARTICIPANT 3:

I think if anybody ever asks me, the opinion of them getting an animal for a child, & my opinion is that, Number 1, they should wait until the child is a little bit older that they'll understand. Them I have a grandson who's two & they've two Boxers & they had two Boxers when he was born & they all get on really, really well & there's no problems. But, ultimately a parent does have to be prepared to do the looking after. I mean the child can be taught to do it & given some of the responsibility, but ultimately if you're not an animal lover I wouldn't say, go on get one.

PARTICIPANT 1:

Because you've got to think of the animal in the long run.

PARTICIPANT 3:

Yes, & another thing I feel very strongly about is again the Christmas thing. A dog is for life but sometimes people don't give enough thought. They get a pet & they don't think about the things like, what if we go to work? What if you go out a lot in the evenings socialising? You go on holiday, who looks after the animal? How long will they left on their own?

I'm not saying that you shouldn't leave an animal on it's own if you go out to work, it can work very well. I go out to work. My family are in & out during the day, but the dogs are great pals & they're fine in the garden but I have to think about the things & the holidays. Do you put them in kennels? Do you have someone to look after them? I'm very lucky because my children are grown up & my son still lives at home so there's someone always there so life went on as normal for the animals but they are things you have to consider.

PARTICIPANT 1:

Well people need to consider them as living, feeling creatures. They're not toys, they're not ornaments, they're not status symbols. They are feeling & have needs.

MODULATOR:

Anybody have anything else to add to it?

PARTICIPANT 3

Well on that question of why you choose that kind of pet. We had an additional thing when choosing the dog. The first dog. When I managed to get a dog as an adult. I wanted one alright, but we'd actually had a burglary.

MODULATOR:

Oh right.

PARTICIPANT 3:

And the Gardai sent out some crime prevention guy & he said, oh you should have a dog because that will just kind of frighten people off. They won't necessarily do anything. That actually gave me the excuse to get the first dog. We already had two cats at that stage & like those cats were so upset the morning after the burglary. I'd say if they could have talked they could have told us who did it, you know. They were really thrown out of their normal routine & everything. They were very kind of distraught or either that it was our emotions they were picking up on. I'm not quite sure.

But I also spent the fist half of my career as a primary school teacher. I always had a pet in the classroom. So we had pet mice & we had guinea pigs over the years & I chose those particular pets because they could live in the classroom & they were kind of easy care pets to some extent, but they were also pets that the children could handle without freaking out the animal & they learned how to care for pets also by that. But, like saying that, I was distraught when they died or something happened to them. They were still my pets. But, it was that kind of thing, giving the kids a certain amount of responsibility & they were ones I could bring home in the holidays & take care of them myself.

PARTICIPANT 1:

That's the problem isn't it. You know sometimes they take it in turns to take them home, but I wouldn't be in favour of that.

PARTICIPANT 3:

No, no. I'd be too much of a control freak to let that happen.

MODULATOR:

But the other thing as well I suppose, is if they came in sick. You'd be worried they did something wrong.

PARTICIPANT 3:

Yeah, yeah. Like I suffered terrible guilt at one stage, I had a terrible flu & I missed ten days of school & when I came back I picked up the guinea pig & I felt a lump on her leg & it was actually a cancerous growth & for ages I just felt, you know, if I hadn't been out I'd have noticed that earlier. Being daft I suppose, but you know that kind of thing.

PARTICIPANT 2:

I was going to say about the security factor of dogs as well. My two dogs again, maybe it's the Irish against Indian, but they are vicious thugs. They are awful dogs. We can't let them out..... We have a dog run for them which we don't use, but if we're expecting somebody to the house the dogs have to put away. Either locked in a room or put away. They interacted..... There's only two other people that interact with them. That's my Mother & my husbands Father & other than that we have to be really careful. Beware of dog signs on the gate & we wouldn't let them near children. They're unpredictable. They guard the kitchen. The food thing, the whole..... you know. I think they have this survival instinct in them that makes them very unpredictable, so it can be a problem for us. We can't put them in to kennels. I've tried twice & both times I've been rang up & asked to take them away. Because one of them hates dogs, hates other dogs, & the other one hates people. So I haven't had a holiday since we came back five years ago......

A LOT OF LAUGHING

My husband does a lot of travelling for his work & again the security of having these two dogs helps. I just know that if anybody came in, they mean it, they would bite them & because of that I can never let them off the lead either. On walks I have them on the lead continuously. If I meet any body, I have to go in to the trees or whatever. Keep them away.

PARTICIPANT 1:

I do that with my dogs. I mean they used to....... I brought two of them over from the I.S.P.C.A in England. One's an Irish one & they used to be fine & I'd go to the park & they'd play with the other dogs but, something happened & I don't know what. I can't do it now. One of them I can just about let her off the lead if there's other dogs around but the other one I can never. If I let him off I'm always looking out,looking out to see if there's any one about & grabbing him. That's why I can't take them all out together. I can only do a combination of two.

But, talking about the burglary thing. I mean my dogs will bark at anything. We were burgled & the dogs didn't make a sound. Honestly.

PARTICIPANT 3:

Well mine do bark but they bark at everything. They bark at my husband they bark.......

PARTICIPANT 1:

Mine bark when you go out of the room.

PARTICIPANT 3:

Ah no, they only bark at he door. If the bell rings or if they hear anyone coming. I often say the cat would probably be a better deterrent , but on the other hand it's nice to know they're there. That they won't let anyone in. I remember seven years ago, my Father's house was burgled & he never had animals himself since I moved out & that's along time back. The Guards at the time told him that they had picked up a guy that they'd caught burgling another house in the area & when they searched him he had a list

in his pocket. He had a list of addresses in the area & beside each number of the house he had a tick as to whether there was a dog or not there. In other words they weren't bothering with the house if there was a dog there & just going to the house next door.

PARTICIPANT 1:

They were going for the not, as a preference.

PARTICIPANT 3:

I'm lucky in that Boxers, they're not terrible barkers. They only kind of bark if somebody comes to the house. Even the cats, they kind of cry if they see a cat rather than bark. But any time they have barked, I've often said to my neighbour I hope they don't bother you & she said, no, I'm delighted to hear it, because if somebody comes to my door & I'm not there, at least they're a deterrent. So they are definitely security.

PARTICIPANT 4:

I find Michael & I.... I have to say I'm hard of hearing over the years, I live on my own for the last six years. Various things. If the curtains are drawn & there's a car pulled into the driveway I wouldn't hear it. She would. She'd be on a mission & I'd say is there somebody there Honey? & she'd go to the curtain & look at the curtain & after a while as soon as a car would come in she would go to the curtain. After a while, the side gate, I wouldn't hear it. Yet she can hear it opening & she's gone to the back door. So it's a great help to me in that way.

But barks yeah. For some reason she has a terrible hatred of anyone with a hat of any description. I've had her since a pup. I don't know any instance that frightened her.....that somebody with a hat would have frightened her or kicked her, nothing like that ever happened, but it doesn't matter what kind of a hat the person has she'll still bark.

I was going home one night & I stopped at a pedestrian lights & there was an African lady with a very large head of hair. Honey got up & had a look & thought it was a hat & nearly went through the windscreen. It was a pretty awful moment really.....

LAUGHING

You know when you're pulled for your tax & insurance. Here was Honey laying down asleep, as she does most of the day...... ahhh just for devilment really, I've let the Guard come up to the windscreen & say “hats Honey...”

LAUGHING

I've got some mixed reactions from the Gardai on that one! But it's a good bit of fun & she does not like hats. No way... a big hatred for them.

PARTICIPANT 3:

But they're the kind of things aren't they that you'd understand watching them. Knowing what it was, as you say, if they had a bad experience, well you could say that's what it was. Why? There's something obviously in her mind.

PARTICIPANT 4:

I wonder why?

PARTICIPANT 1:

I think when you have rescue dogs too you don't know what's gone before. What they're experiences have been.

MODULATOR:

So, we're going to go on to the serious conversation.

What are your views on neutering dogs & cats?

I suppose most of you do neutering, but I mean you may still have views on why to neuter & what other peoples ideas may be.

PARTICIPANT 1:

Well I think they should all be neutered. There's just so many, too many, I mean & I think there should be a restriction on breeding somehow.

PARTICIPANT 3:

I don't know how they would enforce it.

PARTICIPANT 1:

And I've thought long & hard about this & I can't actually come up with an answer to that. I think I really believe that people should have to get a special license to be able to breed animals. And I understand that there are people who want to breed particular breeds of dogs, that they've a great interest in it., that people want to buy particular breeds of dogs. I'm not saying stop it but they should absolutely have to prove that they are genuine about it & be able to take of the animals. That they make sure they go to good homes. There's too many people, like I say I have two Boxers & the first thing I did was get them neutered & the amount of people that have said, they're neutered? You could have made a fortune! Because they're two bitches. How many litters could they have had. If they had a litter every two years you could have made.....blah, blah, blah..... That had never even occurred to me. I agree with you. There's too many animals out there that need homes.

PARTICIPANT 1:

People have funny ideas about it. That it's wrong to do it, that they'll miss having puppies or kittens. I mean, there are so many people that genuinely believe.....

PARTICIPANT 3:

Yeah they believe all those phalluses & it's quite the reverse really. It's been proven that it stops health problems.

PARTICIPANT 4:

It does yeah.

PARTICIPANT 1:

But I mean there are so many unwanted kittens & puppies & older dogs. I mean, it's just.....

PARTICIPANT 3:

Yeah, yeah I think that the reason, I think it should be automatic if you have an animal unless you've got a good case not to because it's a show dog or that you can particularly prove that you're not doing it for silly reasons, like making money. You're doing it because you're an absolute fan of that particular type of animal & you have the proper facilities. I mean puppy farms is another horrible, horrible thing.

Other than that I think animals should be neutered. I think the reason you mentioned briefly earlier about people that don't neuter & I think there's probably two good reasons. One I think is ignorance & they don't think about it. A lot of people don't understand why I neutered boy cats. Why would I neuter boy cats & boy dogs? I don't have boy dogs but I agree they should be neutered. Apart from the obvious things with cats, the spraying & the obvious reasons with dogs is that it calms them down. I wouldn't like to be responsible. I have cats & I let them outside, I think that's fair & good to do that. They should have a certain amount of freedom to go out & about if you live somewhere that's safe for them to. But I would hate to have it on my conscience that they were the cause of some other cat having a litter. So people kind of think it's a boy so why would I bother.

The other point I was going to make there was about the neutering. Emm.... It's gone out of my head now. I'll come back. Somebody else talk.

Oh it's another point of why I think it's so important as to why people don't do it...

Oh yeah.... I think the cost in Ireland is extremely.... I had my two dogs done at the same time eight years ago & I think it was about £350 to get them neutered by the vet.

MODULATOR:

Each, or for both of them?

PARTICIPANT 1:

It was really...

PARTICIPANT 3:

Now I have..... For the two, but I mean that was a one off. That was my bill when I went to pick them up. Now I planned for that & I planned to get them done at the same time because it made sense to me & I had time off work & I was at home with them for that week & it was all planned, but, & I had planned obviously financially. But, that's an awful lot of money & I mean there are people who genuinely cant afford it & there doesn't seem to be much help in this country.

PARTICIPANT 1:

Isn't there a voucher scheme or something?

PARTICIPANT 3:

I saw in the vets last night that there's some crowd offering financial help & you know in the U.K, they do this, neuter your animal by the week or...

PARTICIPANT 1:

If you get your animal from the R.S.P.C.A in England they will do it & it's part of the cost of the animal or they'll give you a voucher.

PARTICIPANT 3:

Do they do that here I believe also? But a lot of the rescue centre's like that, you could end up with one yourself. I know a lot of people that started off by taking in a couple of dogs & before they knew it they had a rescue centre. They.... but they don't have that kind of money to do that.

MODULATOR:

No.

PARTICIPANT 3:

So they have to trust. Now my daughter got a dog from a rescue centre in Leitrim & that lady had to trust that we'd get the dog neutered. I mean, she wanted to do it but she couldn't. So I think mean again, I think Ireland is particularly bad when it comes to animal welfare of all descriptions.

PARTICIPANT 1:

I know you get something from the R.S.P.C.A .You have to sign something to say that you will not breed from that animal. I mean, how they can control it, that I don't know.

PARTICIPANT 3:

But at least they've made you aware. I think that's one of the reasons that people don't do it, but like as you say people have all these misconceptions about how it's bad for them & they have to have one litter before you can do it & how they go loopy & all that kind of stuff.

PARTICIPANT 3:

All my animals are neutered. The cats the dog. One of them managed to get herself pregnant before. Just three weeks before she was due to go to be neutered of course & we kept the kittens. We sorted it out & had her neutered then. But there is in the back of my mind at times, a little kind of thing that says, I wouldn't have my dogs if everybody's pet was neutered.

I'm kind of a Mutt person you know, so my dogs are cross breeds. They're Golden Retriever/Collie cross & they're beautiful. It's a lovely mix. Both of them would have had pedigree mother's that just met a Collie, kind of thing. So in the back of my mind there is always that thing of, I suppose they wouldn't be around if everybody neutered.

PARTICIPANT 3:

No... but everybody wouldn't....

ALL TALKING TOGETHER

PARTICIPANT 3:

I think even if the highest percentage neutered, there would still be unwanted......animals

PARTICIPANT 3:

Yeah I suppose there would.

You know the experience of Sally having the kittens, even though it was something that we hadn't planned. It was my first time to own a cat because my Father was allergic to cats & although my Mother's a real cat person, I grew up without cats & as an adult I got this kitten.

I knew she was behaving strangely but of course, she was in heat, but I didn't have a clue about or quite recognise at the time until a friend says, oh it's lovely to see all the cats playing together in the garden. Then I grabbed her & stuck her in a shed. When we went to the vets she was three weeks pregnant.

You know he actually rang me & he says she's pregnant do you still want her to neutered? I suppose I was kind of struck tat I didn't want to do it but also that I didn't want that to happen at that stage. So, I said no I'll come & get her.

The experience of kind of watching her preparing for motherhood & whatever. I felt that was actually lovely too & she was going around for ages choosing the right spot & took a great love to the seat of the tractor. We thought that was where she was going to give birth, & you know seeing her rear the kittens, even though she had some difficulties as well.

You know we got the off spring neutered as well. They didn't ever have kittens, but there was something about it too.

PARTICIPANT 3:

No well I had a Collie about twenty five years ago & I took her from a rescue centre & like yourself, she was pregnant & we didn't know & my husband says, it's great to see her putting on a bit of weight & after a while we caught on that she was pregnant. We decided to let her have the puppies & I have to say though that I found it a really rewarding experience. Her having the puppies. I found homes, she was a Border Collie & she was a beautiful dog, but the puppies were Mutts. But up to the day they died I saw a couple of them. They all went to good homes. A couple of people I knew. It was actually a wonderful experience.

But your right, the thought of preparing for them. I had read all the books & I had made her a bed, but she decided that the best place to have them was on my couch. So she actually dug a hole in my couch.

The other thing she was a very timid dog. She was so timid when I got her. I actually sat on the kitchen floor with her the night I got her & petted her maybe for three hours & talked to her. She was so frightened. She was so afraid to get up from the bed I'd made for her & afraid to go to the bathroom. At one stage I even thought it would be kinder to have her put to sleep because she was so frightened. But, between giving her the attention & my children were all young & for some reason then. When she had

the puppies, it was like her instinct took over & she came out of herself so much. In a way it was a wonderful thing that she had the puppies because it brought out that instinct to protect them & that in turn..... it was like as though you looked at her & you went.... she went, oh I can bark & nobody will hit me. It's hard to explain but it was like she overcame her fears, but she ended up a better dog because of it. Like I'd never suggest that you should do it, but like I said it was a wonderful experience & I often thought that would be a nice thing to do, that the facilities & the time & the right way of doing it.

PARTICIPANT 3:

Well let's say reservations that you have by neutering. I would still always neuter.

PARTICIPANT 3:

I agree.

PARTICIPANT 3:

And when you mentioned earlier the cost of it. My Mother kind of takes care of cats in her area as well coz she really is a cat lover, & when the family cats died she would mind the feral cats & we kind of realised that she was becoming known as the mad ould one down the road with the fifteen cats in the garden kid of thing & we went about seeing if there was something we could do about it, what we were hoping to do was have them neutered & leave them back in the garden. So at least they'd be there. They'd look after mice & all the rest, but they would actually be neutered. But the cost of doing that for so many cats really really was prohibitive & we did try the cat protection society & the various agencies, to see if there was even part funding available for that, but without really any great success. You know, so like eventually......

I like know cat AIDS exist, you know a lot of the cats did die & they're kind of down to 3 or 4 cats now & a few different people on the road have neutered one each as it were, but have had to pay the full whack for doing that. And again that was really all because it was mostly pet cats. Particularly the Toms that weren't neutered. That was causing this kind of explosion of cats there.

PARTICIPANT 3:

There's this Tom cat cat where I live & I've a neighbour with four cats. They're not substitute children, you know they have children but they love their cats. I have another neighbour with two cats & I have another neighbour with one, but they're all neutered. But, this one isn't neutered & of course he does all the things that Toms do, but he beats up all the other cats, because obviously the neutered ones are more docile. Boy cats. So my neighbour suggested that we kidnap him & bring him & have him neutered. So my husband asked our vet what would she do if he brought this cat in & asked her to neuter it & she said, well as far as I'd be concerned you brought me in your cat & I'd have neutered it. We haven't been able to manage to catch it, but that's kind of the plan, because it's just delicate. We can't go up to the owner &..... but it's a dreadful problem because of this & again I don't know the lady in question. Is it ignorance? Has she not thought of it? Has she a particular reason? I mean it's not like he's a special cat that she might be going to breed him or something like that. And he's out roaming the streets without a collar so......

PARTICIPANT 4:

I em.... The Old English Sheepdog I had for thirteen years, I didn't have her neutered & I don't know why I didn't initially get her neutered, but I didn't & I ran with it. There's various things you can do to offset a bitch conceiving. You know , sprays & all that kind of thing. I found them quite successful. OK, it's a bit of bother, it's a bit of a hassle & you have to watch. We got the through the thirteen years & then I got Honey. I had her neutered & she.... I was taking her from the veterinary hospital & her wound opened & very nearly died due to haemorrhaging. So I think if I got another bitch or a dog for that matter I suppose, I don't know, I'd be reluctant to have her done.

A dog yes I would for the same reason as you, the point you made, you don't want to be responsible for your pet creating a litter of pups or kittens.

It's not a necessary thing I think. It can be got over, you can enjoy a dog for it's lifetime without having it neutered but you have to be vigilant. You have to be careful.

PARTICIPANT 1:

You have to be very responsible.

PARTICIPANT 3:

But didn't you mention that you showed your sheepdog & some of the clubs don't accept them for showing if they're neutered. They make it a point against them, don't they?

PARTICIPANT 4:

The lesser important dog shows are great fun & I'd quite settle for them. You know.

PARTICIPANT 1:

But at least you took the responsibility.

PARTICIPANT 3:

You were responsible. You made sure that she didn't get in to a position.

PARTICIPANT 4:

Yeah yeah.

PARTICIPANT 3:

What we're talking about, I suppose is, you can't trust what people will do so it's better to have a cat blank neutered.

PARTICIPANT 4:

I was lucky.

PARTICIPANT 3:

But you also thought about it.

PARTICIPANT 4:

Yeah...

PARTICIPANT 3:

But there's people who actually don't think about it & they come up with these things. Ahhh she's only six months old sure she can't have a litter yet. You know people come out with crazy things.

PARTICIPANT 2:

I think em.... I've had all my dogs neutered & the short lived cat that I had for a while over the last eighteen months & I had him neutered too, & you know & people have..... people like get a bit, kind of, men especially, they're very kind of squeamish about it & say how can you do that. You're taking away his life's blood. Especially with male dogs. And actually I don't know...... I....... one of our dogs now he was little bit late getting neutered. It must have been a year & a half maybe two because he was a stray, but anyway, I didn't notice any change in him afterwards. He was still a lively very happy dog, no problems at all you know & I don't know & I didn't. You know people said he'd be sluggish, he'd be lethargic,he'll put on weight & all the rest of it. I never saw any change. He was a young happy dog.

There are these myths going around that will change your dog & change their character. I've never seen that.

PARTICIPANT 3:

I've never seen that in any animal. I know there is that some bitches can become incontinent because of being neutered & I was at the emergency vet, as I often am with one of my Boxers, well used to be. He happened to mention, I didn't know this guy, I hadn't met him before but just that in the course of conversation, that Boxers were particularly prone to incontinence because of their nether regions the way they are designed & built. It still didn't put me off. Now she's older, she does have a slight problem. I can't say that that's because she's been neutered because that didn't happen when she was neutered. It could be just an age related thing the way I look at it. But that didn't put me off because that can be treated & she could develop that anyway.

PARTICIPANT 1:

You can't do a controlled experiment.

PARTICIPANT 3:

And it's not a health problem as such. It's just an inconvenience. But the health benefits of neutering are more important.

PARTICIPANT 2:

I do really think there will always be plenty of dogs & cats around the place, you know, no matter how many are neutered. There just will. You'll never get to 100%.

PARTICIPANT 4:

I don't know if you've heard the argument, if you don't have a dog neutered, it can, particularly in a bitch, if you don't have a bitch neutered it can extend her life. Sorry, if you have...... Sorry.

If you have the dog neutered, the bitch neutered, it can extend her life because they don't have to go through the ordeal of giving birth, pups. That can actually add another year or two to the bitch's life span,so that's why I got my present dog neutered & quite intentionally so, because I didn't bother.... I wasn't in a position to breed from her. I didn't want to breed from her & I think that argument has sort of proved itself to me because an eleven year old dog, she's pretty OK for eleven. You know most Labs at eleven are starting to slow up & become arthritic & all the rest. In all fairness now, she's a bit lazy alright, that's about all you know. She seems to be very healthy for her age. I'm glad I did it now for that one reason....... It's worked well for me.

PARTICIPANT 3:

It's obvious isn't it like that they have all their organs removed & they can't develop cancerous things of those organs, so that's going to make their life....

PARTICIPANT 4:

Mine's had two tumours removed.

PARTICIPANT 3:

Yeah, but I mean she hasn't got a womb, so she can't develop like womb cancer or....

PARTICIPANT 4:

No but she had a.....

PARTICIPANT 3:

Other ones yeah.

PARTICIPANT 4:

She had a tumour on the back of her neck & she had another one in her groin & so....

PARTICIPANT 2:

My one is quite bad now. She's got mammary tumours. She's four of them now.

PARTICIPANT 4:

So that would put paid to the argument, having a dog neutered cuts down the risk of cancer.

PARTICIPANT 3:

Yeah but in the areas where you're neutering it does so....

PARTICIPANT 4:

Oh yeah.

PARTICIPANT 3:

PARTICIPANT 2 you mentioned as well about not seeing a difference in the dog before & after. One of my dogs, Zach, was actually seven when I had him neutered & it was I suppose, his early life was a series of unfortunate events. Every time we went to get him neutered he did something dreadful. Got poisoned or you know or had an accident. You know something happened him & we'd put it off for another six months until he was over that & then he'd do something dreadful again. So he was actually seven, so he was a full mature adult of long standing & I didn't notice any changes in him at all, you know, so it certainly didn't affect personality, it didn't affect his weight, it didn't affect anything like that, you know.

PARTICIPANT 3:

I'd imagine that well proves, I suppose with a puppy you could say he hasn't developed enough so you wouldn't know how they'd changed, but I think the only change you would probably notice, in a male dog is that they're not going to want to be roaming off, gong around. But that's a good change.

PARTICIPANT 3:

I didn't even notice that as a change to be perfectly honest you know, because he still has quite a strong prey instinct & I'm surrounded by deer, you know. If he has a chance to get out. There's a pheasant that sits on our fence post & also calls from outside & at eleven years of age he can clear a six foot deer fence if that bird is on the other side of it & he wants to head off. If I wasn't vigilant with him making sure the fence is kept properly maintained, he would go four or five miles.

PARTICIPANT 1:

But not looking for girlfriends.

PARTICIPANT 3:

Well no......

MODULATOR:

Very good.

We might take a break at that. All right.

TEA BREAK

PARTICIPANT 3:

…..she was settled & mature , she was. She just didn't carry on like the puppy, like the puppy was kind of…. learned a lot from her. I got the other Boxer then when the older dog died, it was like the two of them, you saw, as mad as hatters. I can't bring them out walking together because as I said, that's being where you let them of the lead. They run up to everyone & they want to talk to everyone. People get frightened of them, but if they see one it's not too bad, if they see two of them they nearly die so, plus although they've been to school, I have had a girl come to the house for training. I've had them go to all kinds of puppy classes. They've been expelled. Bedlam

So it's just so hard. I'd say to this lady that used to come to the house, to train them, the Boxer that I had as a puppy. I said I have rescue dogs I said & I know nothing about them, I've gone down to the centres, I've brought them home, I've left them off the lead, they've come back when I've called them, they've done everything. I've never had to put a huge effort in to training them & now I have a puppy which should be easier to train & I'm having all this trouble & she said a very good thing to me. She said the difference is she said, when you take in a rescue dog their hearts are broken & they respond to the attention you give them & they're so delighted to give you love & to show you that they want to please you. Where when you take a puppy & all she has known, like I reckon my dog probably came from a puppy farm, that's why I think she's a very bad example of the dog & I think she left her mother too young & that type of stuff but, I wouldn't say she was ill treated on the puppy farm. So she came from having her mother & her brothers & sisters to my house & she never knew any hardship.

PARTICIPANT 2:

And no humans much. You know,wasn't socialised.

PARTICIPANT 3:

I got her at five weeks like so, you know. It's like she just didn't understand that....

PARTICIPANT 1:

Five weeks was very young though wasn't it?

PARTICIPANT 3:

That's what I mean. She didn't really have long enough to develop, I mean.....

MODULATOR:

Dog traits! Basically.

PARTICIPANT 3:

And she comes along & the Collie.....

MODULATOR:

She thought she was human.

PARTICIPANT 3:

The Collie gave her a few slaps along the way. Like this is the way we do things in this house & that's fine, she was good with that. I had to all these things like take her toys from her. Only let her have a ball at a certain time for an hour & all this kind of thing. I mean she's eight & a half now & she's massively improved to when she was a puppy. It was really hard work, but great fun.

MODULATOR:

That's it they're good fun all right.

PARTICIPANT 1:

Our dogs just wind each other up. They really do. They're three clever dogs, they are all three of them just too clever.

PARTICIPANT 3:

But they get on together?

PARTICIPANT 1:

NOT QUITE SURE WHAT IS BEING SAID HERE

The female gets on with the boys. The boys hate each other.... the two of them live in the house but one of them lived outside.....you know from a farm. He never lived inside.

PARTICIPANT 3:

He's happier outside.

PARTICIPANT 1:

So he has a kennel in the shed with hay, so he's OK but....

PARTICIPANT 3 :

He has the comforts of indoors.

PARTICIPANT 1:

Oh yeah... I couldn't have him in with the other boy dog. They'd kill each other., but they just don't.... He'll let the girl dog in to his shed & she can eat his food & she can take his bone, she can do anything, but if the boy dog just as much as looks at the shed. He's off.

PARTICIPANT 3:

It's funny that.... but that's the thing that's amazing about trying to figure out their personalities, because I had two Collies. I'd a Border Collie & she was the one that had the litter of puppies & she went on to get arthritis when she was quite young & we reckoned that it probably was from something that happened to her as a puppy because she was very young when she developed it & when she got a little bit older, that's when I got the Bearded Collie, to bring her out of herself because I felt she was getting a

bit introverted & maybe down to the arthritis & that actually worked. She became the top dog again when the Bearded came.

But, like that they tolerated each other & they accepted each other, but they were never friends. Where as my Boxers sleep together, eat out of the same bowl, drink out of the same dish. They're in fact.... actually I worry that they're too close to each other. That if something happens to one of them......

PARTICIPANT 3:

Are they close together in age?

PARTICIPANT 3:

Two months apart. They're not related in any way. It's just one of those amazing co-incidences, but they're really really close & like I bring them out separately & I try to do stuff with them separate from each other for that reason. & also to spend time with each of them individually. But they are particularly close. In a way I think it's a bad thing.

PARTICIPANT 2:

My two only kind of tolerate each other. They live together. They always have lived together, but she is top dog & he is... he has to behave himself because she is..... She is so obsessed with food that, you know he can not go any where near her & she will just...... she would take the head off him.

You know giving her her tablets at the moment, I put them in to squares of cheese, & I throw them at the two dogs. One each kind of thing. One with the tablet in.....once I got mixed up & the wrong dog got the tablet but..... I throw them at her & if she misses & it rolls over to him, he'll just step back, because he knows if he goes near it she'll attack him. She can be quite vicious with him. You know & this is after eight, ten years together. So there will always, it seems there will be a dominant one. A dominant dog.

PARTICIPANT 3:

Actually one of mine is more dominant but the other one is so laid back that she's OK with it. But then there's no friction & the one that's dominant is the mad one that I had at the classes & all that & she's the one that plays with all the toys & plays with balls & the other one doesn't bother. But occasionally she'll come over & she'll take her teddy bear & she'll take it over to the bed & she'll put it in the bed & she'll lay there looking at her & she won't move. You can't have your teddy bear. She won't fight with her over it. She'll just sit there & cry & cry & cry. Then she'll come over to me. Will you get my bear?

And like every time I buy them presents, a ball or anything like that, I'll buy two. Even though Bobby, Bobby's a girl, even though she doesn't play with them, I still buy her a squeaky toy more so, but the other one then cuggles them all.

PARTICIPANT 2:

That is funny actually the way their characters are so different from each other in every dog I mean. When you think of all the dogs you've had all your life, you remember all of their characters. They're all completely different. Even though they're in the same environment. It is actually quite amazing to watch.

PARTICIPANT 3:

I find it amazing coz I have two Boxers so they're the same on certain traits. They're Boxer traits & then there's certain traits that are personality traits & it's nothing to do with what breed they are, it's just the way they are.

PARTICIPANT 2:

Yeah it's just the way they are. I don't know where that comes from. Like it is strange. They have quirks & likes & dislikes & things that they do that is absolutely unique to them & their personalities. It's intriguing to watch them sometimes.

MODULATOR:

I'm going to bring us back on again if that's all right? Coz we'd talk about our dogs all night. I could do the same myself.

I suppose the next one is kind of still on the neutering theme & it's just about what influenced you decision to have your pet neutered or not, so whether you know, I suppose what I'm kind of looking for here is did you have any outside influences?, or you know where did you find your information about neutering before you got your pet neutered?

I hope you don't mind me going back a few years now at this stage but.....

PARTICIPANT 1:

I just always assumed you neutered your pets. It's just always been something you know that you do.

PARTICIPANT 3:

I must admit back when I had the Collies, twenty five years ago, it wasn't a big thing that people did. It just wasn't. I mean it was there, it was available, but it wasn't something that even when you'd go to the vet that they'd advertise or anything . But after I had the experience of her having the litter of puppies that made me determined to do it & then I realised the benefits of it so the I adopted the attitude of it has to be done. But that is what influenced me initially I suppose, I realised that this could have gone on, year after year she could have been having litters of puppies & then I'd started to read up more about it. There wasn't a lot of rescue centre's around then & that type of thing & not in Ireland anyway. And I started to get more involved in to these things & I realised the problems that were occurring, because up that I suppose I knew people who had dogs, but like was said there, you had them & you didn't neuter them. You didn't have puppies because you looked after them, so it didn't seem to be an issue, in the circle of people I knew, but then when I started to get more involved with rescue dogs I realised the absolute terrible problems & puppies & dogs being thrown out by their owners because they became pregnant & they didn't want to look after them. Left on the side.... & those type of stories you know. So that definitely influenced me.

PARTICIPANT 1:

When I had cats first, I had those neutered because our cats had always been neutered & then when the first dog I got was from the R.S.P.C.A anyway where they advocate it & well now they more or less insist

on it, but at that time, I'm going back a good few years, they would discuss it with you & they gave you the voucher or they did it for you.

PARTICIPANT 3:

I think if you get.... my friend got a dog & a cat from the D.S.P.C.A & they paid a fee for them & in that fee they were neutered. But the problem is that the people who go & buy the dog from.... your dog has a litter of pups & you're selling them. How do you influence those people.

PARTICIPANT 2:

I remember when our first dog. He was the only dog that was an only dog. He was our first & he started wandering & we didn't neuter him immediately & he lived two miles from here & there's like farm land all around us & he was wandering. The people next door to us have a gun dog & a couple of bitches as well & like when ever the bitches were on heat he was living outside in their yard & you know it was getting pretty hectic & we both at the time, we were both working & they rang us one day & anyway more or less said like you know, your dog is in the fields. He could be chasing sheep, he could be doing anything & we kind of realised we're going to have to do something & we actually thought at the time we'd have to get him put down because we couldn't be at home more & we realised that we were kind of you know, we felt we were probably neglecting him. So it was a bit of a shock to us to get this phone call about our dogs behaviour. We were horrified.

I went to the vet & he dissuaded us from getting him put down & said look there are other ways around this & we got him neutered then & I don't think it made any difference. Well it did, it obviously made a difference when the bitches come on heat. But we also cemented the bottom of our fences. Did a lot of things to keep him in & I mean he's the one we brought over to India & he died over there so, you know that kind of intervention by our neighbours was very negative at the time, but actually had a positive outcome & it was partly because of that we got him neutered.

I think we were probably putting it off & then we kind of came to our senses you know so....

PARTICIPANT 3:

Well I had a rabbit & you know there's no reason to get him neutered because he was the only rabbit out in the garden. He wasn't going to see any other rabbits, but when he got a little bit older he got very

vicious & you couldn't go put the clothes on the line but he'd start nipping at your ankles. So I read up about it & I read an article somewhere that suggested that neutering them would calm them down & then he used to attack the dog as well like. The dog would go out in the garden & he'd hop out. But that was my reason & it did calm him down on aggressiveness. I didn't see any difference in him & that was why I did it, although there was no reason that he was that he was going to... somebody having babies.

PARTICIPANT 3:

I think it was a kind of rabbit thing in the back of my mind as well. Although you know I started with cats, they were going to be neutered because I didn't want millions of kittens around the place. But when I left home, there were still younger children at home & my father got rabbits. He got two rabbits & we'd always had a rabbit. Don't think we had two. And dad sexed them incorrectly!

So there was twenty two rabbits at one stage at home & there were rabbits that lived in various rooms in the house because they were different sexes or different ages or different everything. But my mother got them all re-homed & she wanted to make sure the home they went to was a good home & everything else & I think somewhere in the back of my mind, that had an impact on me you know.

That you just do not want to have all that animals & the basic misunderstanding at home was rabbit biology. You know she could have already been impregnated while she had one set or one litter you know, & because the male was whipped out the moment she had the litter. But she ended up with two more litters because they were basically too late you know. So it was that kind of.......

MODULATOR:

It sounds like a scene from Father Ted......

PARTICIPANT 3:

There was one rabbit that lived in the bathroom because he couldn't get on with any of the others.

PARTICIPANT 2:

You'd think you were hallucinating when you went in.

PARTICIPANT 3:

And then divorce proceedings when they ate my mother's.... (heirlooms?)

MODULATOR:

And what about you PARTICIPANT 4?

PARTICIPANT 4:

I think I had my present dog spayed because the guy that persuaded not to. He really did persuade me that the dog would have a better quality of life..... Sorry if I had her spayed that she would have a better quality of life, & now looking back on it I think I...... agree with him & I think if I get another bitch I won't have her spayed. I'll go back to my Old English Sheepdog days & taking the bother of watching & take the sprays & pills & all that. Because it worked.

Certainly after Holly nearly..... well she really did nearly die when I took her out.....the evening I took her out I collected her from the veterinary hospital, it just.... she was an Australian girl & she took me out. We weren't out to the side of the van & I had a bed in the van & all & with that you'd think you were just after turning on a tap.... & a marvellous girl, I never saw a girl to bandage a dog as quick in my entire life. I'd say she'd make a great woman for putting on nappies for kids. She put this......she had the dog bandaged in a minute. Fantastic. But it was traumatic for the dog. It was bad enough like having her neutered & come out then for that to happen. She was bandaged then for a week or two. No I would go back to the old fashioned way that I had & I know it's harder, it's a lot more......

PARTICIPANT 3:

It's hard work & all.

PARTICIPANT 4:

But I think I'd be.....

PARTICIPANT 2:

You're prepared to do it.

PARTICIPANT 4:

Yeah, that's if I ever get another one. The one I have at the moment would be hard to replace, but I more than likely will, but I think I'd..... I'd take enough care & enough bother to watch the bitch. If I ever got a bitch

PARTICIPANT 1:

If you got a dog?

PARTICIPANT 4:

If you got a dog I think I would be more inclined to have it neutered for the same reason, I'd hate to hate the dog to have to get frisky some night & go off & cause somebody else a whole lot of heartache you know. Certainly with a bitch it's a bit different I think. That's just my opinion on it. I know I'd take the chance & not have her neutered. Just put the effort into it you know.

MODULATOR:

That's fair enough.

PARTICIPANT 4:

So there is.... I would make the point that there are another alternative to neutering.

PARTICIPANT 3:

It's a god point because those that are of that opinion will do it.

PARTICIPANT 4:

You can do it if you want, but it's bloody hard work.

PARTICIPANT 1:

But a lot of.....or most people aren't prepared.......

PARTICIPANT 4:

A lot of people can't do it. They wouldn't have the time I....... my lifestyle allows me to do that.

PARTICIPANT 1:

Exactly, but you're still taking the responsibility. I mean, that's what I'm saying.

PARTICIPANT 4:

Oh yeah. You have to do that.

MODULATOR:

Right we've flogged that one to death have we? Although it is kind of a topic that we could talk about for hours and hours.

So we're going to move on to a different topic altogether. Kind of look along,more nutrition & diets & obesity & stuff like that so kind of, so to get your views on home made or commercial diets. What do you feed yourself & is the commercial diet a whole load of propaganda or you know do you all use it? That kind of thing.

PARTICIPANT 4:

I met one lady in Calais, waiting for the ferry the year before last & she was living in Switzerland, she was from England. She was going back across to England with her husband & she was a dog nutritionist & came over & we got talking about the dog & what do you feed her on & Holly being a Lab, she's a walking dustbin. She'll just eat & eat & eat & I have her on this RD Reduction Diet pellets, which are pretty expensive. Whether they're working or not I don't know, but this lady from Calais went to the bother of taking out a note pad from her car & wrote out a diet that I should prepare for Holly.

It was nice now if one could afford it. Like it was free range chicken for a start, boiled.

PARTICIPANT 3:

Nice for yourself!

PARTICIPANT 4:

And garlic. Oh gorgeous yeah....& I said Oh God Hol you may stay as you are. She is..... but I have to watch her. Now in the winter time now & she's not swimming perhaps....she's a little touch of arthritis now which gets to her at, you know in the cold weather, so I don't walk her quite as far &he puts on the weight. But then when spring comes, late spring & the summer she's up & swimming & she'd swim for Ireland & the weight just falls off her & by mid summer August she'll look terrific you know. She certainly won't look like an eleven year old dog. As I can see... now swimming over the last five or six weeks the weight is falling off her. She's tightening, she's not getting skinny but her flesh is tightening up you know.

PARTICIPANT 1:

She's toned.

PARTICIPANT 4:

Like her muscles are toning up.

PARTICIPANT 3:

One of my Boxers has..... one of them you couldn't put weight on her no matter what you fed her, it's just her nature.

PARTICIPANT 4:

There are dogs like that.

PARTICIPANT 3:

I had them on Royal Canine food for.... dogs with sensitivities to food & the reason that I have them on that is that they developed, I'm not even going to go in to this, but they developed an alopecia problem two years ago and they had extensive tests done two or three years ago to try to find out what the problem was & one of the things we did see would it help was put them on this diet to see was it food related allergy & they both got the same problem at the same time. It absolutely was ruled out that it wasn't anything, like initially you would think maybe it was ring worm or something contagious. There was nothing. They went to U.C.D. & they had extensive tests done & they stayed over night & they're tests went to England & they came back & everything was negative. So we still don't know & one of them still gets it. So we just put it down to, they lose their winter coat & during the time of their summer coat for some reason they just lose patches of hair more in spots. But it's kind of their flanks here & it's a sign of.... it can be a sign of a thyroid problem & I suppose that's what people were looking for but anyway that's another story. But that's why they're on the food, but one of my Boxers I say she....no matter what you give her to eat, she'd eat rings around you, but she's just so fit. Runs around so much she never ever ever sits down. I'm convinced, she's eight & a half & I'm convinced that one day she'll just drop dead, because she actually doesn't think she's getting old, she thinks she's a puppy. And even when I have her out of the house with a ball, people say, how old is that dog? & I go eight & a half. That dog couldn't be.... the other dog is much more sedate & she has arthritis & she also has a crusciate problem with her knee & at one stage maybe two years ago she was in extremely bad with that & she was supposed to have surgery for it & we thought long & hard about the surgery because it was so involved & what it was going to put the dog through. But we luckily have found some medication that works really really well & you wouldn't know to see her that she has the arthritis. But when she had it & because of that she doesn't exercise as much as the other dog. So she did have a weight problem & does have a weight problem if I don't watch her. But I just control it by feeding her less, because there's really not a whole lot I can do because as she's improved with the arthritis so well & I can walk her more now, she has slimmed down now. But at the time when she couldn't take the exercise the only other thing was to give her less food. The problem is she's a dog that just loves her food.

PARTICIPANT 4:

It's very hard to get a balance....

PARTICIPANT 3:

That's all I could do.

PARTICIPANT 4:

You could walk it off them, starve it off them if they can't walk

PARTICIPANT 3:

And it's so hard because she'd be there looking at you like you know. But you have to do it like you know. That's how we managed her, & of course that in turn helped with the arthritis when she slimmed down so. But like she's doing great I mean October two years ago I brought her to my vet & I pleaded with her to put her to sleep she was in so much pain & she's running around like a puppy now.

PARTICIPANT 4:

Somebody was telling me that, maybe Modulator you could verify this one, that a great substitute for food per say is chopped carrots. That it doesn't actually......

MODULATOR:

I might afterwards. I try not to interfere when you're talking

PARTICIPANT 4:

It's just something that somebody said to me at one stage. A dog trainer actually that a great way to con a dog is to chop up, dice some raw carrots.

PARTICIPANT 1:

They think they're food?

PARTICIPANT 4:

Like you know they think that its food yet it doesn't put on any weight.

PARTICIPANT 3:

Well now I don't know now that you'd convince them.

LAUGHING

PARTICIPANT 3:

That's why I use commercial food is for convenience. I've always used dry food again for convenience.

PARTICIPANT 1:

And its hygienic...

PARTICIPANT 3:

Yeah.... Obviously if they don't finish it. I believe in the theory of I leave the food out for them & because they're Boxers they get fed twice a day because they can have a tendency to bloat, so I give them a small meal twice a day rather than one big meal. I always did kind of feed dogs twice, I always felt one meal once a day was maybe not enough,but again it's my opinion. I don't give them extra food by doing that. I just split it between two meals, but I wouldn't leave.... if they don't eat it after a short time I take it up & I don't give it to them, but the other thing is cleaning up after them. If they're on a dry food diet it's much easier.

PARTICIPANT 1:

Especially with cats. Cats will only eat, they;ll eat when it's fresh. Even they're hungry they won't eat it. You know with the dried food it's quite expensive, it's more economical in the long run.

PARTICIPANT 3:

Absolutely yeah.

PARTICIPANT 1:

And I get that TD stuff for their teeth. It cleans their teeth as well.

PARTICIPANT 3:

And I did read up about, there's a diet called a BARF diet. Which I think is bones & raw food. And it's basically that you feed them raw vegetables & raw ones from the butchers & apparently you can actually give them chicken wings because once they're not cooked they're OK. And I did read quite a lot about it, it's supposed to be a wonderful diet. But again you'd need the money & you'd need the time.

PARTICIPANT 1:

At least you have to get the balance right. At least you know with the bought stuff....

PARTICIPANT 3:

Yeah that amount for their weight & that amount for because they're on a diet. Or that amount because you know they're getting all the nutrients.

PARTICIPANT 2:

Yeah because the first five years I think, it would have been five years while we were in India, with the dogs, there was no commercial food out there. So the dogs were fed a mix of this, I'm vegetarian so the thought of these things are.... these chunks of meat.... & I couldn't even go into the shop that....we had a driver so the driver used to get the huge things of meat & fill the freezer. It was absolutely awful. Luckily I didn't have to cook it either.

PARTICIPANT 3:

I was just going to say, did you have to prepare....

PARTICIPANT 2:

No no, the woman we had that used to work in the house, she used to feed all four dogs every day. I'm sure it wasn't her favourite part of the day but you know her job was fairly easy besides that so she put up with it. But so for the first five years they probably ate goat meat & bones & rice. And she'd make a huge pot of this kind of stew of this. Put in the meat & rice & water & a bit of salt & it became a grey

gluey kind of soupy gunk, but the dogs loved it & they ate that every day & I'm sure like when you compare what's in the commercial stuff, it didn't seem like there was much nutrition in it but they thrived on it. Most people in India wouldn't get that ever.

PARTICIPANT 3:

That's what I was going to say. People wouldn't get that.

PARTICIPANT 2:

Coz they don't eat that much in India.

PARTICIPANT 3:

I remember when I was a child & there wasn't really commercial dog food then & you know I can barely remember that you could buy Bonio biscuits, mixed ovals & tins of Pal, but there was no such thing as a mix of dry food with Pedigree Chum or something like that.

A friend of mine, her Father used to keep Alsations, & one of her jobs was to in the evening after school to go to the butchers & get a sheep's head & some light's they were called. I think they were the innards, & he'd like boil them up with brown bread & that's what the dogs got fed.

PARTICIPANT 2:

That's very good like coz now they eat the dry Pedigree. I get a big sack of that.

PARTICIPANT 1:

When you changed them to that did it make any difference?

PARTICIPANT 2:

No I don't think so.

PARTICIPANT 4:

I don't know what your woman was on about her free range chicken. That was way out. She was actually very annoyed that I was actually feeding the dog what I was feeding her. You said I shouldn't be ding this & she shouldn't be having that. Crikey the recipe she gave me was a yard long you know & like you, you boil it all & you make two weeks supply of it at a time you know.

PARTICIPANT 2:

You'd have to be very dedicated.

PARTICIPANT 4:

It's a bit of a yarn. Ah no, you know sure maybe if you were bringing her to Krufts or something like that, but even then at that I think it's....

PARTICIPANT 2:

Well I think those things are.... well they must be because... well our dogs are.... I don't know but dogs do seem to live longer now. With better care, you know I'm sure their nutrition is better because of the mixed stuff than what they wee getting because basically they were scraps of meat & whatever....

PARTICIPANT 3:

Scraps of the table. You know they probably weren't getting a balance diet. Well maybe I mean if you're mixing vegetables with meat with a good quality brown bread or rice or something like that you're getting a balance, but years ago dogs were lucky to get fed. People were lucky to get fed so you know it was table scraps & bits of bread & stuff.

PARTICIPANT 2:

Sometimes I do think the commercial stuff, the dried stuff seems to me to be very salty, because they go through buckets of water. Now I don't know is that just the Pedigree stuff? If I try them on other stuff they wont eat it.

PARTICIPANT 1:

That's the trouble of getting them to eat it because one of mine, she's terrible she just won't eat, & I mean she's had a tooth removed so that problem was dealt with but she still won't eat & I have to mix a bit of tinned dog food in with it or make gravy for her so I buy the Tesco's low salt gravy mix & stuff or you know if we've got any other bits left over. Sometimes I put little bit of olive oil or any oil just to give it something. I don't know why she won't eat. Most peculiar, but I can't leave it out because the other dogs will get it.

PARTICIPANT 3:

I lived in the West Indies for a while & again there was no commercial dog food or anything like that, so we had two dogs there & they were fed rice & chicken. Which I just like used to boil up everyday for them. Back here, Zach who's my eleven year old, when he was a puppy he was on a dry Pedigree or one of those things, a puppy food for a year or so & then when it came the time to change him on to the next kind of more adult food, I got the one for eighteen months, that kind of thing you know or whatever & he just had a really bad reaction to it. You know he'd have the runs & all kinds of stuff & I'd an awful job trying to find a food that he could actually eat. So I put him on a rice & chicken diet for a while. Again a vegetarian house hold but we cooked the rice & chicken for the dogs. You know someone had said to me about putting children's vitamins in to it as well you know the vet he was interested in nutrition himself at the time. So he was on that for a while & then what I actually did was I'd get a small bag of various different kinds of dog food & try mixing in a bit you know & then the next day another

little bit & so on & eventually I found a food that he could eat. So for years I wouldn't feed him anything else because this had suited him. And the new dog when he arrived had to eat that as well because like you know..... the same kind of idea.

Then when I moved up to Sally Gap, the one day the shop would have the bag & the next day then you'd go & they wouldn't have it. I could not get a continuity of dog food. So we've actually ended up trying out all sorts of kinds of things even Lidl dog food when we're stuck. He's obviously much better now but you know I still be really careful if I had to change the food I'd try to do it over a period of a few weeks & mixing bits together you know.

PARTICIPANT 1:

They say though that you shouldn't have any problems changing from one dried food to another. It's not supposed to be a problem. You know it's changing from wet food to dry food you do have to do it gradually.

PARTICIPANT 3:

I've heard people say that they're dogs have had diarrhoea & so probably difference in diet. You're probably.... you see that's a huge problem especially if you get any of the you know ones you can't buy in the super market. You know I have to get my food through the vet & she always has a bag for me so it's grand, but other than that you know I remember when they puppies I had them on Eukanuba or something like that & the pet shop that would have would run out & then you'd have to try & find another place & then you'd have to buy it before it would completely run out & you'd be getting a bag of Pedigree Chum to tide you over. That's a bit of a problem really so I'd say that's why a lot of people stick with the Pedigree Chum or the Mara or any of those that they pick p with their weekly shop & have it that way like.

MODULATOR:

So I think we're kind of..... we're talking about weight. What about the factors that you feel have influenced the weight of your pet is it just the diet? Some of you were touching on that already.

PARTICIPANT 4:

What's that sorry?

MODULATOR:

It's what factors, do you feel, have influenced the weight of your pet? Sorry I was getting a bit scrawly as I was getting down the page. I was looking at it like this.

PARTICIPANT 2:

Well age is.....

PARTICIPANT 3:

Well age & less activity I suppose...& like they say....

PARTICIPANT 2:

Injuries. Leg injuries & that kind of thing.

PARTICIPANT 3:

Yeah like Bobby with her arthritis but she's not going to take as much exercise. But I... people say when you get them neutered they put on weight but often think that's it's just a co-incidence . That as you get them neutered, they get a bit older. Especially when you have children, I find that it's mush easier....

Bobby has a weight problem & it's much easier to watch it because she's at home with us & it's just really me & my husband that's feeding her. Where as when my children were young, it was the biscuit under the table & the bit's that fell on the floor & you know so they get a lot & even like even like ourselves you'd say ah she didn't eat that much more but, what you think isn't that much more, often is a lot.

PARTICIPANT 4:

If you put it all into a bowl at the end of the day....

PARTICIPANT 3:

Or it might have a lot of calories in it. It might not be a huge amount of food but, like a slice of brad & butter or a piece of cake would have an awful lot of calories in it. So I do think..... I don't think neutering makes them put on weight. I think that co-incidentally they can put on weight but & people blame it on the neutering. That's another reason they why people don't neuter them.

PARTICIPANT 2:

We have a problem in our house because one of our dogs is very much over weight & she's the one with cancer at the moment as well. It's a bit of a problem because I want her to lose a bit of weight so I can exercise her a bit more & it's kind of a vicious circle isn't it. Any way when I say we've a problem my husband is much more..... he's much softer than I would be with the dogs & you know they have to have their morning Margi they have to have their afternoon Margi & the dogs just sit & look at the cupboard & he always gives in, always. I'm telling him you know & the vet is telling him would you stop feeding the dogs & he finds it very difficult to say no to them & he knows its for their own good but he still feels that if he's eating you know they have to eat too you know this kind of business & because of his sense of fairness as well if one dog gets something & one dog is not over weight, he's fine but then the female dog who is has to get... OK only half a Bonio where the other one will get a full Bonio.....

NOT SURE WHAT BEING SAID HERE

PARTICIPANT 3:

…. give each a treat but give a little bit.

PARTICIPANT 2:

So when he's working, he's working abroad this week, I have full control over the thing & I can make sure that their.... I'm stricter that way you know they'll get something....

PARTICIPANT 4:

They'll be looking forward to the next feed.

LAUGHING

NOY SURE WHAT'S BEING SAID HERE

PARTICIPANT 2:

That's awful... they sit looking at you saying God is he here..... so I'm a bit of dictator.

PARTICIPANT 3:

It must be an awful problem for you then if she's sick. Yeah coz you can't exercise her you're only option is to cut down on food.

PARTICIPANT 2:

She's OK but she's not, she's quite, she's lagging behind in the walks. She can't do the full walks & now, only this week now she's having problems jumping in to the back of the car so now she's getting, I'm putting her in the front, she's in the foot well at the front. This has just happened this week so it's a kind of gradual thing but yeah. Yeah it is a problem.

PARTICIPANT 4:

I'm sometimes having dinner at home, you know usually it's out in the scullery at the back. If the door from the kitchen to the scullery is open, if I'm having dinner here she'll come & she'll sit & stare & it's very hard to ignore her, it really is. And I keep saying as she's getting older a little bit won't hurt, but it does so you have to resist doing that but what do you do when she gets up, comes over & noses the door open& out & arrives back in like Oliver with a bowl in her mouth, & she will sit there indefinitely with the bowl in her mouth.

So I had a think about this & it's amusing & it's nice in it's own way so to try & counter act anything I would give her, I give her maybe a half an inch less in the cup the next time.

PARTICIPANT 3:

That's a good idea.

PARTICIPANT 4:

Whether it balances out or not I don't know but it's pretty hard to say no. You know yourself.

PARTICIPANT 3:

They'd get awards for it wouldn't they? Begging & the big eyes & you feel so mean & you can't even eat your own food then because you feel so guilty & you know they've just had their dinner, It doesn't matter.

PARTICIPANT 2:

We're doing things all wrong really because you know what I've read about you should always eat where they have to wait. You don't do it, the dogs always get their dinner first

PARTICIPANT 1:

I always say it keeps them quiet I think it doesn't but I think it does.

PARTICIPANT 3:

Another thing, I don't know. Do you feed your dogs at the same time every day?

PARTICIPANT 2:

Yes every evening.

PARTICIPANT 1:

Yeah.

PARTICIPANT 3:

Well I actually have a thing about I don't & the reason that I don't is that I rad somewhere that if you do feed them we say at 7pm in the evening & there's an evening that you don't come home until late, they'll get upset & they'll get upset & they'll get to know this routine. I never really knew if that was true or not but I just didn't do it. There wouldn't be a huge difference it might be 7 tonight it might be 7.30 tomorrow night, 8 the next night. The mornings is kind of the same time because it's before I go out to work. But an uncle of my husband's we went to visit him & he had three Bearded Collies & these people had been living abroad & they came home & I hadn't seen them for years & years & we went for Sunday lunch & we sat & we chatted after the lunch & whatever & at 4.30 the three dogs got up & they started to bark, & we thought is there somebody coming in & the uncle says no it 4.30 it's diner time. They actually knew it was 4.30 & they got up & they barked. Not bark , bark, bark, bark. Just a little....

PARTICIPANT 2:

A reminder.

PARTICIPANT 3:

It's dinner time. Out & get the dinner. S I don't know now about that one.

PARTICIPANT 3:

I noticed mine used to.... because it was so handy with the dried food, when I would come in from work, park the car or whatever & feed the dogs & then go in & put my own dinner you know & it dawned on me then that when I would come in in the car from anywhere they associated that with getting fed so I actually changed my routine a bit like that too.

Now I do tend to feed them at the same time because I feed them when I come in from work but I might actually come in, put on my own dinner, or just hang out the washing or just do something different first. But I do.... I am actually very strict about what I fed them & I only feed them the right amount according to their weight & you know they get a few treats for certain reasons you know. At night if I call them from the garden because I've two acres. They prefer to stay out there barking at deer rather than come in. any dog that comes in the first times he's called gets a treat, so they actually do come in now on demand & I often think if I could be as strict about my own diet!

I don't know how I can do it for them, but I just give in to myself then all the time you know what I mean. I can actually do it for them & it does actually pay off. One, the eleven year old has a touch of arthritis you know but he's the right weight, he's healthy & fit.

PARTICIPANT 3:

Well one of treats that I give our dogs are those denti sticks & I've been giving them to them since they were puppies & really didn't know if they helped or not, you know they're big dogs & they say the best thing for teeth is raw bones but then the raw bones there's other problems with them, but it's just a habit & it's last thing at night when they get they're treat before they go to bed & I must say they're eight & a half & neither of them have tooth decay so whether its that or the food or what but that's their treat. But like that they wouldn't go to bed without it. When they come in & sometimes you let them out maybe at the weekend when we stay up a little bit later, & they're always in the house really unless we're going away for a long period of time then it's just they stay out in the garden but I mean if we're gone out for several hours but, you might maybe let them out at 11pm on Friday night but we wouldn't be going to bed. They come in & they go like where's the treats. You can't say treats. One of my dogs you can't say W.A.L.K because they'll get up & get their lead. And we started off with that like we'd say will we go for a, you couldn't say walk so we stared saying W.A.L.K, but then she understood that so now we call it a W. but it's amazing the way they do get to know things.

PARTICIPANT 2:

Ours are the same but if they don't get their going to bed Margi or whatever it is, they won't settle & they'll bang down the door. Especially the female, she will just whack the door continuously until she's sorted. Yeah they can be very demanding.

PARTICIPANT 3:

It's funny the things like that. I worked with a guy & his mother used to walk the dog every night after EastEnders. She always used to walk the dog at that time. So the night that EastEnders would be on, he said there'd be no problem with music at the start, & the show would start but then the music at the end when he heard it for the second time he'd get up & right we're going for the walk & it's just amazing. Like I mean I bring mine for a walk in the evening & as I said I bring them separately & then particularly because I have this fear of them getting bloated particularly the one that runs around with

the ball. And they shouldn't exercise.... you shouldn't feed them within an hour exercising. So obviously when we come home then I say well you can't have your dinner for another hour & I'd be kind of watching the time like. But it's amazing some nights I mite go out later just because of my routine & I say the walk might be later so the dinner will be later. But they still come in at the kind of sort of the time that they get the dinner & they look at you.

PARTICIPANT 2:

I am convinced that dogs have some kind of an internal clock. It's partly to with signals like ether music or something you might say or whatever, but I think they have some kind of internal clock as well because one of dogs goes out for a pee at eleven o'clock every night & we could be watching something & she will start getting restless at exactly eleven o'clock. Now we're just sitting just watching something on TV, it's not, there's no signals, nothing, but exactly eleven o'clock.

PARTICIPANT 1:

And what happens when the clocks change?

PARTICIPANT 2:

I've never..... ha ha ha ha ha

If she's still a live for the next one now I'll.... never thought of that.

PARTICIPANT 3:

Sometimes I've noticed that with cats actually when the clock would change, the cats will still come at the original hour for their food & they'd just sit on the window sill & you'd go Oh Gosh they're an hour early or whatever. But it was because he could......

PARTICIPANT 2:

Jet lag or something....

PARTICIPANT 3:

Yeah that it took them that few weeks to actually....

PARTICIPANT 2:

Adjust.....

PARTICIPANT 3:

And I do think the dog must be the same.

PARTICIPANT 4:

Where I live it's about three quarters of a mile away from the village. I bring her up & back. It's slightly further. I live in Sallins. On the Clane side of Sallins, so I come up through the village, up around the railway station & back. It's a good mile up & a good mile back so that's every night of the week hail, rain or snow. But on Sunday evening, five o'clock or six. In or a round five or six, I go for a pint. There's a nice beer garden & I bring Holly & I've been doing that for the last five or six years, & come five o'clock. Hail, rain or snow. Dark or bright she's up & she's on. She knows, knows, knows.

A couple of weeks ago there was a walking day in Punchestown & I brought her out & I took her up there & she was very clapped out now by the time I got her back to the van. That's grand now I thought I'll go for a pint on my own tonight & have the luxury of sitting up at the counter rather than in the beer

garden. I went in, & the fresh air had zonked me, & I had forty winks. The next thing I got the nose, she was tired but she was still going.

MODULATOR:

Now I've heard of excuses to go to the pub on a Sunday night, but I think that's a new one.

LAUGHING & TALKING OVER EACH OTHER HERE

CAN'T MAKE OUT WHAT'S BEING SAID.

PARTICIPANT 4:

I don't think I really need excuses......

It helps you convince yourself at five o'clock on Sunday evening.

MODULATOR:

You have to go for a pint, that's about it.

PARTICIPANT 4:

Medicinal purposes. But it is amazing the way she cottons on to the times. Particularly the night after Punchestown, now that's it I said she's not going to make it tonight, she won't be interested. I dozed off &.....

MODULATOR:

Rudely awakened....

PARTICIPANT 4:

Oh yeah.

MODULATOR:

The next two questions are kind of one question I suppose. Kind of like how important is your pet.... Like how important to your pet is the exercise routine you have? If you have an exercise routine? And how important is it to you to spend that time exercising your dog, or playing or your pet cat or whatever?

PARTICIPANT 4:

I think it's very, very important to have, if it's only an hour every evening. Any kind of a good walk is going to take you an hour anyway. I think, in my case anyway, a dog means something to you it's worth an hour a day of personal time with the dog. If I don't walk the dog for some reason, I feel guilty. I feel as though I'm not being fair to the dog & by gor she can let me know that at times too but with know are you coming. But no I think that important to have, it might sound a bit OTT but, call it quality time or whatever you knw.

PARTICIPANT 1:

It's a bonding time isn't it.

PARTICIPANT 4:

It's a bonding time.

PARTICIPANT 1:

It's maintaining the bond.

PARTICIPANT 4 :

Yeah, yeah. Maintaining the bond. Thank you for putting it so well. Yeah bonding time.

PARTICIPANT 1:

I feel very guilty when I don't, & I often can't manage. I mean different reasons. In the winter I find it very hard because where I live I can't walk on the road at night because it's dangerous, & I can't walk off the road because that would be equally dangerous & I probably fall in the lake or something anyway. Soi find that very hard in the winter that I don't get them out enough. And because I can't take them all at once, you know I'll take one, one night & two the next night you know, I try & even it out & they do get out in the garden you know but I do feel guilty when I can't manage it. It is hard to do. And it's funny because I used to live in Liverpool & you'd think it would be hard walking your dog in Liverpool. It's easier to walk your dog in Liverpool than it is in rural Ireland. No sheep there, lot's of parks but no sheep & the pavements. You know it's crazy, I'm in the country by the lake & I can't find anywhere safe to walk the dog.

PARTICIPANT 2:

Yeah we're the same. We have to put the dogs into the car. We can't actually walk out our gate....

PARTICIPANT 1:

Yeah, exactly.

PARTICIPANT 2:

So we have regular walks but, in the winter time, like you I can only take one or the other dog. So in the winter time I'll either take take one or the other dog. In the summer time then when it's bright in the evenings & my husband's home, we bring the two dogs but it's fine because we probably felt that as much as the dogs that, my husband is not..... I would always walk. Dogs or no dogs I love walking but he has often said to me that if it wasn't for the dogs he wouldn't walk at all. He knows that himself & we'd be having massive rows because you'd always want to going walking & I just don't see the point. Where he loves walking the dogs &it's a great stress relief for him too because we talk about work & everything while we're walking the dogs so everybody benefits. So you mean like it is.... the dogs are on leads all the time so they don't probably get as much exercise as they should because they're not they're not running for miles. If you can leave the dog free its great.

Even earlier on when you were talking about Holly swimming. One of our previous dogs, Saber, she used to swim miles in the lake. She'd just go out in to the lake, & she'd just be exhausted when she'd come.... she just loved loved swimming. None of our other dogs do, & we used to leave her off the lead because she was a very placid dog & there was never any problem. I do think having them on the lead coretails their exercise. It's not great but what do you do. Like we have a fairly big back garden so. One of them the male dog, he runs around & there's horses & he goes up & barks at them & that kind of thing but the female dog she's so lazy, I have to put her on the lead to bring her down the field, because she will not... you know she won't do it herself you know so she's just that kind of dog.

PARTICIPANT 1:

I used to find that early in the morning is the safest time to walk but I have to leave the house at 7.30 now & I can't... I get up at 6 as it is. I mean I can't get up any earlier.

PARTICIPANT 2:

You'd meet yourself going to bed!

PARTICIPANT 3:

I must that I'm fairly lucky in that where I live I can kind of walk very short distances on the parks & greens & I let them off & the more placid dog doesn't run around that much so even lead walking is fine for her. Where mad hatter she'll chase a ball around the field & if she hasn't done enough ball chasing & we haven't gone around the field often enough she won't come back so sometimes like tonight I had to cut it short because I had to come here & she told me that this wasn't a proper walk like. But at the week ends we might bring them off. Put them in the car & bring them off & ring them on really long walks through the woods & that type of thing. But I think it's a very important time as PARTICIPANT 4 said to bond with your dog. To spend with you dog, like even though your dog is with you in the house, you're busy doing things. You're watching TV, you're doing house work or you're talking to other people, but when you're out walking with your dog you're just walking with your dog.

I particularly find it important for me because as I mentioned my dogs are very close to each other & because I walk them separately, it's probably the only time I spend with them individually. And that to me is very important because I think it's very important that they don't depend on each other too much. So & then the exercise element of it I'm mean I do like walking & I would go out walking but I wouldn't go out every night & I wouldn't walk as far as I do if I didn't have a dog.

PARTICIPANT 1:

And you can you can go out more safely when you're with ….

PARTICIPANT 3:

Of course yes

PARTICIPANT 1:

And I mean even….. when I've been back over to Liverpool, I've gone for walk on my own without a dog & I've been on my own without a dog, I've been in the park without a dog & I feel well I shouldn't be doing this, this is weird.

PARTICIPANT 3:

I don't understand this. I don't know what's that's about like. I remember my husband went through a period, just bad time business wise & he couldn't sleep at night & he used to get up & he used to bring the dog for a walk & he'd come back home & I mean like maybe at 2 or 3 in the morning. And we live in a town like, you know like & it's not like you wouldn't meet people. But often said to me that he would not have been able to that without the dog because he would have felt that people would have thought he was up to no good.

PARTICIPANT 1:

Yeah. I say particularly for a man it's......

PARTICIPANT 3:

You know I could have walked around here at 2am, with the dog I probably still look a bit peculiar, but at least I was out walking my dog.

PARTICIPANT 1:

You had a reason for it.

PARTICIPANT 3:

Yeah.

But it does make you if you, want to cut the walk short kind of if you're being lazy, the dog will make you & you'll say no. and because the guilt thing if you don't bring them. Even tonight, I felt so guilty because I cut Zara's walk short.

MODULATOR:

Tell him I'm sorry.

LAUGHING

PARTICIPANT 3:

But it is funny that.

PARTICIPANT 3:

I kind of I suppose resonate what the rest of you have said & while I love walking myself, they make me go out in weather & at times that I wouldn't do it myself & because also where I live I actually walk mine on the road which seems really, really sad. Surrounded by 40,000 hectares of national park but I can't let them off the lead you know because I can't trust them with the deer & stuff around. So we do actually walk one the road on a lead & I have tried all sorts of different leads. I have a couple lead, I've tried taking them separately. I have Zach walking perfectly to heel but, he knows how to do it beautifully, but when Denzel came in to our lives Zach wouldn't walk to heel any more he had to be ahead & then Denzel had to be ahead of him & then I was skiing behind them. And you know I put a lot of effort in to training them to walk properly on leads & stuff like that so that we're all safe but, it's dark up there in the winter. There's obviously no street lights & in the winter months there's a lot less traffic which is great so I would actually go out at any hour of the night in the dark. We just put on our...... I've to reflective jackets for them as well & walk up there & people often wonder why I would do such a dangerous thing but I don't feel it dangerous at all I feel really safe up there actually out on roads.

PARTICIPANT 1:

I feel safer walking the dogs in the dark even in the morning than I do at night, because it's getting lighter & the mad rapists aren't likely to be up that early

PARTICIPANT 3:

You meet people who are going to work & are more sensible hopefully.

PARTICIPANT 1:

And another problem I have is physically holding them. You see I have very strong dogs & they're not... like I haven't been able to train them. They're Collies. I can't train them to walk properly to the lead. Although one of them will walk to heel without the lead.

PARTICIPANT 3:

And have you tried head collars on them?

PARTICIPANT 1:

Oh I've tried every thing. I tried the thing around their mouths....

PARTICIPANT 3:

That's how I got my Boxers......Yeah that's what I mean yeah.

PARTICIPANT 1:

Yeah, yeah, no. in fact one of them that I had the halty on & an ordinary lead just in case..

PARTICIPANT 3:

I've been there too.

PARTICIPANT 3:

One of mine can get out of it. I don't know how, I don't know how. My husband reckons it's the neck. She's got a short neck & she can get the head head out. The other one doesn't but they have worked for them. Other than I wouldn't be able to manage them. And they're actually.......... you know its not bad training, it's excitement. When they're on the way out they're pulling, when they're on the way back they're grand. It's excitement, I want to get out, and I suppose most dogs are lke that but if they're not strong it's not a problem. But & then with the Boxers with the bad knee & the arthritis I don't want her doing that because it's not helping her either like.

PARTICIPANT 3:

I think there's another thing to exercise as well. It's not just the physical thing of exercise, but the sort of mental stimulation for yourself & your animals as well, bringing them out.

I was really interested PARTICIPANT 4, in you holiday, & the fact that the dog travelled with you like that. This year for the first time, I actually managed to bring both of ours. We went to Achill for a week & we had a house that would accept pets & it was just so lovely to see their excitement going on the beach over there you know & going somewhere new where they hadn't been before & you know the older lad, we used to live by the sea here in Wicklow, he used to swim in the sea, but the younger fella had never been swimming in the sea before. Just to see him you know taking a few steps in & a wave coming at him & he dashing back & you know all that kind of.... I don't know..... that you're actually challenging them mentally & stimulating them you know. Same this for me obviously, I was getting that & see them, their reaction.

PARTICIPANT 3:

I must say when my children were younger we used to take them on holidays in Ireland & we used to bring the Collies. Like that I must say it wasn't too difficult to find a house that would accept them. There's not a huge amount, we'll say like hotels in..... like in the UK there's loads of hotels where you can bring dogs to. In Ireland there's very, very few. Which is an awful shame really, but I think it's a lovely idea to be able to take them on holiday

PARTICIPANT 3:

Well what struck me was I had to pay another 50euro to bring the dogs, & I thought, children I know would do a lot more damage....

LAUGHING

PARTICIPANT 3:

I've actually out off going away sometimes. Like my son still lives at home & if I need to go away, he's there & care for them but because of his lifestyle he's not always there & I've never had to put them in kennels & I wouldn't now put them in kennels for one night to go away for a week end type of thing like coz they've never been I kennels. So I've often gone to a function we'll say & driven home, rather than stay over because of the dogs.

PARTICIPANT 3:

Which I do all of the time.

PARTICIPANT 3:

And people would say ah you can't have a glass of wine, & I say no I'd rather take the car & drive home, & be home tonight.

PARTICIPANT 1:

My husband & I, we never go away together because we can't. One of us has to has to be..... I wouldn't trust anybody. Because other people have said you know we'll come in &...... my dogs are so peculiar that wouldn't expect anyone to come in & deal with their behaviour. So we just don't.

PARTICIPANT 3:

That's why I made reference earlier on in the conversation, some people just don't think about those aspects of owning pets. I mean some pets are fine you can just put them in to kennels. Kennels ae expensive though & you have to have the kennel cough vaccine yeah, & then you have to find kennels that you're happy with. So, but they are things you have to think about before you take an animal as you say....

PARTICIPANT 1:

Well I just couldn't afford to kennel home them...

PARTICIPANT 3:

A friend of mine in work she has two cats & when she goes away she puts them in kennels but even two cats is quite expensive.

PARTICIPANT 1:

Yeah it is.

PARTICIPANT 4:

I wouldn't be able to put Holly in a kennel because I'd get such a hard time for about two weeks afterwards. But if I have to go away for perhaps three or fur days, I leave her with some of the children you know. When I was away particularly in Germany, you'd get a freshly laundered pet bed to take to the room. Done up in a polythene bag. 4Euros. In some of the hotels yeah. If you go in to a resturaunt & you can, the first they do is take out a bowl of water for the dog & then you get the menu.

PARTICIPANT 1:

They do in France as well don't they?

PARTICIPANT 4:

France they do yeah...

PARTICIPANT 1:

You can take dogs in to......

PARTICIPANT 4:

...I've stayed in. I ended up in a Chinese resturaunt in Milan one Saturday night. I was thinking not the safest place for a n over weight Labrador. Holly with green pepper & a side order....

The same thing applied. The bowl of water out for the dog & I got my glass of wine & I got my meal & two seconds later the waiter came out with a little bowl of fried rice & would your dog like a bowl of fried rice? Would she what..... So yeah it's a totally different culture..... particularly in France.

PARTICIPANT 3:

Ireland is very, very bad.

PARTICIPANT 4

Sure you can't even take them in to a pub

PARTICIPANT 3:

I mean some places are hard pushed to allow guide dogs in. I was in pub near where I work, one day having lunch with my friend from work who is absolutely terrified of dogs & this lady came in with a guide dog & she wanted to complain that they had allowed the guide dog into the pub. I said it's a guide dog!. I do think it's really bad that a well behaved animal can't be allowed....

PARTICIPANT 1:

They allow children & they're all over the place.......ha ha ha ha ha...... but they are!

PARTICIPANT 3:

We used to bring our Collies away more than we do with the Boxers but we used to ask, you know if we were away on holiday, if we went in to a pub for something to eat, during the day. I'm not talking about at night when there's music on & there's people out for the night. Most places were pretty OK. They took one look & yeah you seem OK, but you had to ask, it wasn't accepted & then we were in holiday kind of places in the country side. So I wonder what other places would be like.

PARTICIPANT 4:

I pulled in to a hotel one night in France. Down in the south of France on the way back. It was hotel on the out skirts of a town. It was a lovely little spot so pulled in & out come these two guys in kilts. My God I said, it's nice to see a few Scots men. We're not Scots men, we're Welsh. I didn't know up until that stage that thee was Welsh tartans & that sort of thing. But they had great time for me & great chat & they were at their sisters wedding she was marrying a French guy & the reception was on in the hotel. Come in later on, have your meal & come in & join us. Which I did. I changed & had a meal & left Holly in the van & they were all very upset because I hadn't brought the dog in. I had to out & actually bring the dog in & then at one stage the bride & groom got their photographs taken with the dog. I mean it's just..... I mean it's a completely different culture out there. Completely different.

MODULATOR:

I think we've covered all of our things. You're welcome to keep talking about your dogs if like. Get more coffee & more tea. There's no problem at all but thank you very, very much.

END

Location 3 focus group 1

MODULATOR:

To start off with is, we want to know why do you have a pet? What's the reason did you get a pet for in the first place? &what you feel feel took you to doing that? You want to start off *Participant 1*?

PARTICIPANT 1:

Well I have always had a liking towards animals and my family are from a farming background so I suppose you'd kind of a, what's the word, a tendency or an orientation towards animals. So you're kind of brought up around animals. So I've always had a liking of dogs. I suppose it's the, you know they say dog's a man's best friend and they're great companionship & I'm kind of an animal person I suppose.

PARTICIPANT 2:

You could say that about all of us in the room. We're all animal lovers.

MODULATOR:

And I suppose you've had a few over the years. Can you remember your first one *Participant 2*?

PARTICIPANT 2:

Ah indeed I do. When you were free to go on anyones land.

PARTICIPANT 3:

That's true yeah.

PARTICIPANT 2:

I've always had huntng dogs & I always had greyhounds. Dogs were part of rural life weren't they?

PARTICIPANT 3:

That's it I suppose. You're either a dog person or you're not. You either love them or you hate them. You know some people if they see a dog they'll run a mile in the opposite direction & some people just don't.

*PARTICIPANT 4*NTERS LATE

MODULATOR:

So what about you *Participant 5*? You just have pets from when you were born or do you have a special tendency towards different pets?

PARTICIPANT 5:

This is the first we have.

MODULATOR:

First dog?

PARTICIPANT 5:

Yeah.

MODULATOR:

And is there a reason for getting it or.....?

PARTICIPANT 5:

No, not really. I like dogs.

MODULATOR:

What do you think *Participant 3*?

PARTICIPANT 3:

I grew up with dogs, coz my brothers did a lot of shooting & then when I got married, I married a man with a lot of sheep, so we got a sheep dog. And over the years the children grew up with the sheep dog & I saw a connection that the children even had with the sheep dog, even though she was a working dog. So I got a smaller pet for them & from then got another one & I ended up minding them all. Cat's, dog's, so that's how I came to have them.

MODULATOR:

So do you want to tell us a little bit about your pets?

PARTICIPANT 3:

I have Cavalier King Charles, and anyone who knows the Cavalier, they are friendly. The sheep dog at this stage is sixteen so....

PARTICIPANT 4:

That's a great age.

PARTICIPANT 3:

You know at this stage she's nearly at the end.

PARTICIPANT 6 HEALY ENTERS

MODULATOR:

So do you want to tell us what you have *Participant 1*?

PARTICIPANT 1:

Oh, it's Cavalier King Charles aswell. Same. They make lovely pets, & the size of them, they're very friendly, & they make great house dogs. The fact that they're small is good. They are very friendly, very much people dogs, they love the attention. They're placid aswell. A kind of placid personality. They're not very exciteable.

MODULATOR:

And what do you have *Participant 2* these days?

PARTICIPANT 2:

A Shitsu. He's part of the family. Everywhere I go he'll go.

MODULATOR:

You said he's two is it?

PARTICIPANT 2:

Maybe going on three.

MODULATOR:

What about you *Participant 4*, what to do you have?

PARTICIPANT 4:

I’ve two cats.

MODULATOR:

Two cats.

PARTICIPANT 4:

I had three but one of them died six months ago. They’re all tabby’s coz I love tabby’s. I love striped cats.

PARTICIPANT 6:

You wouldn’t like a few ore would you? I have a few more.

PARTICIPANT 4:

I just love striped ones.

MODULATOR:

And as I was saying earlier on before you came,that the reason for doing this is to try & get views & ideas about some questions that I have. I suppose, I did some research already & a lot of the research we do is just telephone surveys & written surveys. Basically they just ask a question & you give the answers. But sometimes we miss the right answers. That we wouldn't have all the answers that other people would have. So, the idea is to get your ideas & your answers that are free for you to give, rather than have to pick them up from a list. So that's kind of what this is about. To explore what your ideas are.

So the first question we're asking is, why do you have a pet?

PARTICIPANT 4:

I love all animals. I feel a terrific affinity with them. I learn an awful lot from them & I get terrific pleasure from watching them. From observing them, the way they move. They are so graceful & so alive & absolutely beautiful to watch. I get terrific, tremendous, I get an awful lot back from owning animals.

I have had a dog in the past too. He was an Alsation & the same went for him. A beautiful mover. Highly intelligent. Unbelievably clever. I absolutely adored him.

SOME CONVERSATION ABOUT FILLING THE FORMS WITH *PARTICIPANT 6*

MODULATOR:

Are you up to speed with us *Participant 6*?

PARTICIPANT 6:

Don’t talk to me about animals.

MODULATOR:

We’re talking about animals *Participant 6*.

PARTICIPANT 6:

They just seem to arrive on the door step.

MODULATOR:

If you’re here for the football, you’re in the wrong spot! (ha ha ha ha ha)

ASKING ABOUT TEA, COFFEE OR WATER

DISCUSSING THE FORMS

PARTICIPANT 6:

Officially, i have thirteen cats. There's two more after arriving in. I got them neutered. I told them to go home. One went home for a few days & came back again. Everything is neutered.

I even had a goat over night.

MODULATOR:

So you've got lots of animals anyway?

PARTICIPANT 6:

Oh I have yeah. I've just lost a German Shepard there recently. I had to have her put to sleep.

MODULATOR:

I'm sorry about that.

PARTICIPANT 6:

I had her thirteen and a half years.

MODULATOR:

Wow

PARTICIPANT 4:

Mine was ten & I believe that they don't live much longer. That's the normal life span.

MODULATOR:

Yeah, they wouldn't live much longer than that.

PARTICIPANT 4:

Because I couldn't believe I was losing him at ten. I kept saing to the vet he's going to recover.

PARTICIPANT 6:

I had her put to sleep about two weeks before she was fourteen. She would have been fourteen.

PARTICIPANT 4:

That's a decent age, you know.

PARTICIPANT 6:

And she was eating so well.i had to put her into a standing position to keep her going & she had no quality of life. The quality of life was sort of non existent.

MODULATOR:

It’s always a hard decision to make alright.

PARTICIPANT 6:

It was very hard to decide on it because she was so with it. But you couldn’t leave her on her own at all. If you moved away from her she’d be whineing. The last day I came home from work, I remember, I put the key in the door & I could hear her whingeing inside & she was under the kitchen table. She just wanted to know there was someone there.

I’ve had four of them now, one after the other. They’re a brilliant breed to have.

PARTICIPANT 1:

Alsation’s?

PARTICIPANT 6:

Yeah.

PARTICIPANT 1:

German Shepards?

PARTICIPANT 4:

My mother wouldn't have any other breed.

PARTICIPANT 6:

They're hard work initially, when they're puppies.

MODULATOR:

They're bouncy!

PARTICIPANT 6:

You need plenty of energy. I've a cross at the moment, between a Shepard & a Husky. Talk about a nut case! She was re-homed to Waterford & she was about to be put down & I took her on. Well I can manage her. I've a big run, so it works ok. If you want a relaxing situation, it's not the dog to pick up. Well the Husky in her I'd say is the lively part.

PARTICIPANT 4:

Because German Shepards are usually placid.

PARTICIPANT 6:

I always get a bitch & have her spayed at about seven months & then I don't have much problems after that.

PARTICIPANT 4:

I had a male, but he was very gentle & very placid. He loved to play & Billy could run & run & run & run, but he could lie beside you for hours outside on the grass.

PARTICIPANT 3:

Do you not think an awful lot depends on how you handle them from the beginning? The Shepards are very different from the Cavalier. The atmosphere they live in. You must have a lot of control & energy.

PARTICIPANT 1:

Yeah, with Cavaliers, if they don't get a lot of attention, they miss it. They need a lot of attention. So I suppose it is a lot to do with the environment aswell.

PARTICIPANT 6:

And the other one I have is.... someone asked me one day what breed he was. I said " very rare breeds". He has so many in him. He has Collie, Terrier, at least. I don't know what else. The tail was cut off him when I took him in. He should have a Collie tail but he hasn't. Again he was supposed to be re-homed but......

PARTICIPANT 4:

To disturb that..... any dogs tail......... cutting.

PARTICIPANT 6:

I took him in the day before Christmas Eve about five or six years ago & the following morning I got up & I saw blood in the run & I thought, oh God, Parvo. I got into the car looking for a vet & I at last I got The vet. He says bring him in & I said no, come out, coz I don't want to bring it in if it is. He came out & smelled the boot & he said that's not Parvo *Participant 6*, that's dietry.

You see he'd be thrown.....he wasn't given proper puppy food. He shouldn't even have been off his mother at the time. And of course no one was going to re-home him, so he never left my place. I just got him neutered & kept him. Vaccinated him.

MODULATOR:

So I suppose the big question then *Participant 6*, is why do you have so many pets?

PARTICIPANT 6:

Well we grew up with animals literally, you know from day one. There was always anmals in our place. My father wasn't a farmer but we have a small amonunt of land & there were always three or four cows, & they all had names, & there was always a dog or two, & there was always a few cats, & we even had a few ducks & chickens at one stage. So, when you grow up with animals I think you'll automatically have them the rest of your life, I'd say.

MODULATOR:

Just as we go along, yhe idea would be that, I don't ask all the questions. To talk aroud the points & get involved with conversation. Just like you were doing. What I'd like, if you wouldn't mind, is that ye don't talk over each other, talk in groups. It means I can't hear both of ye & the tape recorder can't hear both of ye either & it maks it harder.

The other thing you notice, you might think I have Tourettes,because every now & then is I'll say someones name. The idea is so the tape recorder, when we're transcribing, that we know whos talking. So, if you hear me mumbling that's what I'm doig.

PARTICIPANT 4:

They do that on the radio.

Yeah, that's what'll be going on

It's kind of someting you've already answered in a way. Why do you choose the type of pets that you have? Have a think about that one.

PARTICIPANT 6:

The reason why I have so many is that I'm a volunteer with the I.S.P.C.A here in Location 3 & a lot of them are actually left on the doorstep of my home. Usually the ones with a problem. You get them sorted & you become attached to them & it's too difficult to hand them back then. This is what happened with the two dogs & the cats.

Last autumn I came home from a faamily funeral & there was a kitten, an adolecent kitten on the doorstep with the leg hanging.she was laying on it. I ended up taking her in & she ended up having the leg amputated. And you know you go through all that recovery with them & you're not going to hand them back out again. When you've put that much in.

MODULATOR:

Get so attached to them.

PARTICIPANT 6:

Yeah, I had the goat for the night, the litle kid, & it was a killer handing it over, but I was afraid he'd be stolen out of the field so, he went down to Ballycross Apple Farm. Down south of the county. You can become attached so quickly & so easily.

MODULATOR:

It sounds like your animals find you, rather than you finding them.

PARTICIPANT 6:

All the cats have found us more or less. They've all been rescues or a couple were feral originally & we tamed them down. That's it.

PARTICIPANT 3:

There were three cats around the area. Remember, I found them a brought them into The vet. Three were let out around the area & I brought them in to Ashley & you collected them.

PARTICIPANT 6:

You see, I do go every so often to a santuary in Rosscommon & as long as they don't come into my household....

MODULATOR:

You're safe enough.

PARTICIPANT 3:

Somebody dumped them on our road & they went in to a couple of houses. Tiny little kittens, hardly off the mother. During the wet & the cold & I already had a cat & I said I don't want another cat.

PARTICIPANT 6:

For a time there we could afford a cattery, but I'm afraid funds are gone so low, that's gone out the window. Yeah, we can't afford to board them, so what I do is, I left carrieres out today to people & I'll collect them on the morning we're travelling & take them that day, any of them we can catch.

MODULATOR:

Ah right...... So you have dogs & cats?

PARTICIPANT 3:

Well I have a cat thats real old. She must be fifteen or sixteen. I said she had many operations, nine lives & our lives gone in the middle of it. She attacked me the other night when I was tring to take a wool ball off her. And she's still there because she grew up with the kids.

And I have four Cavalier & we have a sheep dog.

MODULATOR:

Was the sheep dog chosen for work?

PARTICIPANT 3:

Wel he was chosen for work, but the kids when they were small, had made a pet of him, so he's stll hanging around. He's not much good for the sheep now, he's going blind.the back legs are gone & he's more of a pet really.

MODULATOR:

Why did you choose a cat & a dog?

PARTICIPANT 3:

The children wanted a cat & we got cats when they were small. I suppose the same with the dogs. The dogs are lovely. We grew up with dogs so, & the interaction with the children when they were small with the dogs. I find it when I meet people now,they find the same thing with children.

My daughter used to have the sheep dog & when she'd come home from school she used to take it off down the field. And she told me years later, that she'd tell the dog all her secrets. When ever someone was bullying her in school or any prolem, & even to this day she's going around on the back legs, & that girl comes home & I even said it's on heart, arthritis tablets & I said they're very expensive. I wa explaining away to her God the price of these tablets every week. 8 – 10 euros, & she said to me, mammy I'll pay for them out of my saturda job. She loves the dog that much.

Some people say through the years, children are always looking for a dog, & I have found through talking to people, maybe where parents get separated, or if there's a lot of problems in a home, that children, you know, can cling to a dog & at least. You know she said that dog, when they'd have a row in school. She'd come home & she'd go down the field & she'd talk to the dog.

Now, whether the dog understood her or not ! (ha ha ha ha ha)

She said to this day... A lot of people would say the same thing, that the dog was really a confidant.

PARTICIPANT 6:

She had a captive audience you see anyway.

MODULATOR:

Somebody was definitely going to listen to you.

PARTICIPANT 1:

The Cavaliers particlarly. Theres's one Cavalier in particular I would talk to it like it's a human most of the time. Like the same as if it was a person. You know I'd have full blown conversations with it

PARTICIPANT 3:

Some of them can be very intelligent. You know the way you'd notice in a group of dogs, one would be a lot more intelligent & some of them you'd imagine, they knew what you were saying, but I think its routine. If you've a routine it's like getting up in the morning at a certain time. Those dogs will let you know you're laying on.

PARTICIPANT 4:

I observed something astonishing once with the Alsation I had. We had been living in Ireland with him & he was playing with my mothers dog & my mothers dogs name was McCool & she came to visit us weekly. We went abroad wit Max our German Shepard & when she came over to visit us a year later, the first thing she said to Max was "where's McCool?". He ran to the window & he look ed in the garden & then he ran into another room & looked out that window & he went around the house looking for McCool. He got so excited just at the name & went straight to the window. We were….. we stood there frozen. We couldn't believe our eyes.

MODULATOR:

He understood what you were saying.

PARTICIPANT 4:

He did. He understood.

PARTICIPANT 1:

They’re very intelligent

PARTICIPANT 4:

He understood McCool. He remembered McCool & that was a year later & he was looking for McCool.

PARTICIPANT 3:

He probably associated your mother. The dog with your mother.

PARTICIPANT 4:

It wan’t until she said the name McCool. That was the trigger. He went straight away to the window.

MODULATOR:

It’s a cat you have now *Participant 4*?

PARTICIPANT 4:

Two cats. Two tabby’s.

MODULATOR:

And you used to have dogs aswell?

PARTICIPANT 4:

Yeah, well I grew up with dogs & cats & then I got my own German Shepard & then he died & I got cats after that. I would have got a dog, but, this is personal. They discovered a heart complaint & I was afraid then to get a dog. If you leave a dog behind you. I didn’t like that thought. Cats are somewhat independent & it wouldn’t be so hard on them. But thats why I don’t have a dog. That’s the only reason. I would have had one. I would have had a German Shepard.

MODULATOR:

Ok.

PARTICIPANT 4:

And now I’m sorry when I first decided to not have one, I didn’t, because now he would have been ten. That’s why I ont have a dog.

PARTICIPANT 2:

We had a Collie dog seven years ago, & I also have a special boy, a very bad epileptic. The Collie was able to know when he had an epileptic seisure coming on. No matter where she was she was able to sense it.

PARTICIPANT 3:

Collies are very intelligent.

PARTICIPANT 6:

Very intelligent dogs.

PARTICIPANT 2:

Unbelieveable. No matter where she was she was able to feel, sense the rate of the heart for the epileptic for Patrick.

PARTICIPANT 3:

And what way would she react? What would she do?

PARTICIPANT 2:

She would go crazy. That's what she'd do. But I believe Alsations can do the same. They're able to sense it.

PARTICIPANT 4:

I wouldn't be surprised.

PARTICIPANT 2:

Collies can do it. I believe Alsations can do it too, but the fella we have now, if Patrick was having an epileptic, he'd go bite you. That's the way he reacts to it.

PARTICIPANT 6:

When he senses it coming on, is it?

PARTICIPANT 2:

The one we have now don't, but the one before, she was unbelievable. She couldn't speak, it was the only thing she couldn't do. That's the only thing. She'd get Patrick & he'd clean her face & when Nellie would get the dish of water, Sheila would go to the back kitchen & get the towel for to get her face washed. Talk was the only thing she couldn't do.

That was the one that The vet had to put her down. We coudn't bring her home, we had to get the neighbour out in Clonhaston. There was a big funeral for her. But that was the only dog I've ever seen that was nearly human. Unbelievable. If Patrick was walking, he was able to walk that time, he can't do a whole lot now, but tha time, & there was a pool of water Sheila would always walk between him &the water. Or if there was a car coming up the driveway she would always look to see where Patrick was before she'd go to the car.

PARTICIPANT 3:

So protective of him.

PARTICIPANT 1:

They're amazing aren't they.

PARTICIPANT 2:

Unbelievable. But every time, a second before Patrick got the seisure, she was absolutely brilliant because we'd get no warning. Just go like that! But we always got a warning from her, that we'd be ready for him with the dog. Unbelievable.

PARTICIPANT 4:

Fantastic.

MODULATOR:

And why did you get the dog that you have now?

PARTICIPANT 2:

Well we got him down in Mallow in Cork. Fiona bought him down there. I would always have to have a dog. Deirdre is the same with Patrick. Dogs are great for any one with a complaint like that. They're part of the family. But Collies are absolutely brilliant.

PARTICIPANT 1:

Thay’re very theraputic, I think. Dogs to be around. I dont think you can ever be in a bad mood around a dog.

PARTICIPANT 3:

Well at least.....

PARTICIPANT 1:

I mean if you are in a bad humour you won’t be invited to be around for very long. Hat’s what I think.

PARTICIPANT 3:

Well a dog sense is great. He knows when he’s not going to get away with mischief.

PARTICIPANT 6:

Well the other thing is they make you exercise. When you have a dog taking them for walks.

PARTICIPANT 1:

That’s true yeah.

MODULATOR:

I’m just gonna stick with you for another second. Why did you get a dog this time or... do don’t have cats?

PARTICIPANT 1:

No. No cats. It was for a pet. Well there was a couple of the Cavaliers, well, we bred from them, but there’s one Cavalier that’s just a pet solely. I suppose they’re a good looking dog, really if you can say that. They look cute & they’re small I suppose aswell. And they’re placid & have a nice personality & good with families & friendly, so I suppose that was the reason for choosing that breed as such.

MODULATOR:

And why not a cat then?

PARTICIPANT 1:

Not cat people.

MODULATOR:

Fair enough.

PARTICIPANT 1:

My sister was a cat person but that’s all. Nobody else. Can say that I’m definitely not a cat person.

PARTICIPANT 4:

I find cats very charming I must admit & they can be extemely affectionate. I have a big male cat & I only have to appear & I can hear him purring. You know, that is what I love about animals too, they're not posers & they can't fake anything.it's sincere, you know. Unlike people. I tink their onesty is absolutely fantastic. I love them for many reasons & dogs the same. Dogs are full of affection, & that's very flattering really. Makes you feel good about yourself.

PARTICIPANT 3:

But do you not think that hey go to the person that feeds them & looks after them?

PARTICIPANT 4:

Not only. Ah no...

PARTICIPANT 3:

But cats are kind of supposed to be independant. They come to you when they're hungry & then they'll go.

PARTICIPANT 6:

Not totally independant. I don't think so.

PARTICIPANT 4:

The way that rub up against you & walk around your legs you have to be careful not to walk on them. They're overflowing with affection.

PARTICIPANT 6:

Well I have one fella & I took him home from Location 3. He came out of drain in church street. His mother must have dropped him crossing the walls & he fell down in a drain. He was handed in next door soaking wet & wrapped in a damp towel one night & I happened to be there. It was another collegue.she says I have to go to work in the morning, what am I going to do with him?. She says will you bring him home & of course I brought him home.

He was even crawling with fleas. I brought him home that night literally to die. I wrapped him up in a towel, & I wrapped tin foil around him aswell to generate a bit of heat. 2.30am he came to life & I could only get a drop of water into him. So, the following morning I tore into town & got some LACTOL or some of those suppliment feeds, & we were trying to figure out what we'd put on for the fleas. Eventually anyway Natasha talked to Larry or Joe or someone, it was just the Moyne that we happened to go in to, & they chanced a bit of FRONTLINE on him to kill off the fleas.

I brought him home & I bottle fed him & when I finished bottle feeding him, my German Shepard that died recently, she was quite young at the time, & she used to stretch out on an old couch. A chaise longue type couch. Full stretched out & I'd lift her leg & I'd put hi under the leg when I was finished feeding him.The next thing you'd hear the sucking. He used to crawl up along her chest & up to her lip & suck away.

Up to the day she died he'd come in & every night he'd throw himself at her. Now he's five, six, maybe seven years with me & he'd throw himself at her & he'd lick her two eyes & he adored her.

If I tried to pick him up & give him anything he'd bite me & go for me & he'd attack me. As far as he was concerned, shewas his mother. Do you know even as an adult he adored her.

He got into the bed beside her & he wouldn't suck her, but he'd suck the fake bed, & he used to suck that beside her. He's unbelievable.

She died anyway & he went missing for two days after.

Last Sunday week my neice was down from Waterford & she has a German Shepard. Similar to my one but not as pointy a face, & we ahd been down the fields with the three dogs & she came back up & her dog was stretched on the middle of the floor after the tear around. This guy comes in, struts over, throws himself at the dog & the next thing the two of them sit up & stare each other. It suddenly dawns on him that this is not his mother!

He thought she was back after two weeks. He ran like nobody's business.

But like he didn't look on me as his saviour, he looked on her as jis saviour. Yeah she was his mother a far as he was concerned.

He was only two or three weeks old when I took him in. But it was the dog he took to. She kept him warm & he had comfort from her.

PARTICIPANT 4:

I had one cat when I had the dog. They played up & down the stairs & around the tables chasing one another & when the dog lay down the cat would lay on top of him. They were terrific pals. That was a tabby aswell. They were both highly intelligent. I swear the tabby's are so intelligent.

MODULATOR:

Participant 5 you are very quiet over there in the corner. Do you have anything to add? Why did you get a dog? Can you remember?

PARTICIPANT 5:

We always had a dog so....

MODULATOR:

Do you like dogs?

PARTICIPANT 5:

Yeah, I really like dogs.

PARTICIPANT 6:

What type? Or what breed?

PARTICIPANT 5:

We've a Bichon Friese

PARTICIPANT 6:

Oh gorgeous! They're beauties aren't they.

PARTICIPANT 5:

Yeah.

PARTICIPANT 2:

Really intelligent little things.

PARTICIPANT 6:

Yeah they're lovely things. A male or a female?

PARTICIPANT 5:

A male.

PARTICIPANT 6:

Ah right.

PARTICIPANT 3:

Why did you pick the Bichon over the Shitsu?

PARTICIPANT 5:

We were looking on the internet & we found the Bichon doesn't shed any hairs. So we just said our friends have one & they're a nice temprement.

PARTICIPANT 6:

Yeah, they're a lovely temprement.

PARTICIPANT 3:

Would ou find them more intelligent than the Shitsu? Or would you go for one again? You know if you were back agan.

PARTICIPANT 5:

Definitely.

MODULATOR:

Is there somebody in the house allergic?

PARTICIPANT 5:

No. Just.......

MODULATOR:

You just didn’t want to clean it?...... (ha ha ha ha ha)

PARTICIPANT 6:

It saves a lot of hassle especially at this time of the year. This is a hairy time.

PARTICIPANT 3:

You have to trim them though don’t you? Every six weeks or so?

PARTICIPANT 2:

Yeah.

PARTICIPANT 3:

There's a good of of work trimming them I think, isn't there?

PARTICIPANT 4:

Oh is that so? Do they get fluffy if you don't?

PARTICIPANT 5:

It gets very matted.

PARTICIPANT 4:

Very matted.

PARTICIPANT 6:

I have to do that with my pure bred mongrel. He's unbelievable at times. You just have to get the scissors out & clip away.

PARTICIPANT 1:

The Cavaliers shed a lot of hair. I don't know if you noticed. They shed a lot of hair you know we keep them trimmed up as best we can, but they do seem to shed an awful lot of hair. Opposite of a non shed dog!

PARTICIPANT 3:

I was watching a programme on the television, you know Pete the vet, he had a Cavalier & they showed this type of comb. And I got the comb & it's really great for grooming them. Really take ou half of the hair. Less hairs around the place. It's great. There's a bend on it. A little bend on the comb. There's ways around it.

MODULATOR:

They keep you busy alright.

PARTICIPANT 3:

It's a full time job keeping them, isn't it?

MODULATOR:

Yeah, keeping them clean.

I’m going to move on just a little bit from the general pet conversation, to neutering. Again as I said in the study we’ve some interesting kind of scenarios, demographics on neutering & stuff & I think there’s some kind of a mix. Some of you have everything neutered. Some have some neutered. I’m just wondering why you got them neutered? You know, what influened you to make the decisionto neuter your pets? And if you didn’t get them neutered, why you didn’t neuter your pet?

That kind of stuff. See what your different views are on that. So, if you want to volunteer any information.

PARTICIPANT 1:

Well none of Cavaliers are neutered. Not that we’ve made a concious decision not to neuter them but I suppose I just, I don’t know really, it was never an issue. It wasn’t a concious decision that we said we’re never neutere but, none of them are. I don’t know, I can’t really give a reason as to why. They just aren’t. I suppose it was never an issue.

MODULATOR:

Are they indoor dogs then?

PARTICIPANT 1:

Yeah, they’re all.... wellthere’s one particular pet which is completely indoors. She’s a total house dog. You know, someone’s always with her if she’s outside. I suppose with her there’s no need for her to be neutered. The others are in outdoor houses. They’re kept under control.

MODULATOR:

Are you breeding some of them?

PARTICIPANT 1:

Yeah, some of the Cavaliers yeah. It was just never an issue.

PARTICIPANT 4:

I didn’ neuter the dog but he was never loose outside either. There was never any chance he was going to get himself into trouble, because he was either inside with us or outside with one of us, you know, but I did neuter both of the cats. I’ve a male & a female. I did that because I didn’t want the tom to get himself in to troble in other peoples gardens & things like that & I didn’t want the female to have unwanted kittens galore. And, I believe it prolongs the females life.

PARTICIPANT 6:

It does.

MODULATOR:

Yeah.

PARTICIPANT 4:

So those are the reasons.

MODULATOR:

Prolongs the males life aswell.

PARTICIPANT 6:

Well they’re not fighting & spreading disease.

PARTICIPANT 3:

Well there was an old, through ignorance, people used to say if you neutered or spayed a dog it would get vey fat afterwards. You know, there was various reasons & I suppose way back before people kind of started treating dogs as pets, you know the way, the dog was outside. It was more like a farm animal. I’d say that when we got the Collie she was never neutered simply because she was alwys under control & there was no one living near us. So all of us made sure she was tied, you know the way, up in her pen.

Cavaliers I think are the opposite, because there’s always a thing about Cavaliers being stolen & I have a terrible fear that your dog will be stolen. Even if you let them out for a minute & they take to the road. Cavaliers have no road sense. They’ll go with anyone. I always sort of feel, if they are stolen they can’t be bred from.

PARTICIPANT 6:

They can’t breed.

PARTICIPANT 3:

That’s the reason why I always neuter or spay the Cavalier.

PARTICIPANT 1:

Well that's a good point actually about being stolen. Particualr with the Cavaliers as a breed tend to be stolen quite a lot. You hear of it.

PARTICIPANT 6:

Specially small breeds. All small breeds.

PARTICIPANT 3:

A regular thinf of people contacting me that their dog has been stolen.

PARTICIPANT 6:

Yeah, we get that every day. We're getting all these lovely small breeds. Cavaliers & Bichons. You really need to micro chip tem all.

PARTICIPANT 3:

Well even with micro chips you're not going to find them, unless they're found. But, at least if they're neutered they can't continue breeding.

PARTICIPANT 6:

But thing is if they escape aswell, if they're picked up by the dog warden or go in to a vets they can be sc*Participant 4*d.

PARTICIPANT 3:

Oh yes, it's important, but the right person has to find them. I know of people who have lost a dog & it has been micro chipped, but if it gets in a puupy farmor in to some one who wants a dog, they don't bring it to the vet. But, if the dog has pups, you don't know the neighbours dog down the road. You know indiscrimminate maybe crossing with a big animal. That's the only reasonwhy a small dog why I think they should be.

MODULATOR:

Especially with these new designer breeds, where they don't actually have to have papers. Coz they're not acyually pure bred.

PARTICIPANT 6:

Labradoodles & what have you.

PARTICIPANT 3:

What's this you call the Bichon crossed with the Cavalier?

MODULATOR:

Cavalon.

PARTICIPANT 4:

Oh, Cavalon. Is that it? There's another I've heard of. Is it Cavadoodles?

PARTICIPANT 6:

No. Labradoodles.

PARTICIPANT 4:

Oh yeah, Labradors & Poodles.

PARTICIPANT 6:

And they're very placid.

MODULATOR:

What about you *Participant 5*, have you neutered?

PARTICIPANT 2:

No, he's not neutered. Ah sure he's around us all the time. There'd be no reason. He's under strict control around the house.

PARTICIPANT 5:

He's supposed to be.

PARTICIPANT 3:

Well just, I don’t want to take all the conversation but, you were talking about dogs being stolen & you know, you’re in to picking up dogs. I’ve heard several breeders saying they’ve locks on their kennels at night time. Kennels being broken in to & pups & everything taken.

PARTICIPANT 6:

They’re going for sale.

PARTICIPANT 3:

They’re going literally in to the wrong hands.

PARTICIPANT 1:

They’re probably going to a ferry straight away or it could be to anywhere.

PARTICIPANT 3:

Or into a puppy farm, or into someone who is going to breed them & not going to register them.

PARTICIPANT 4:

I often wonder, you hear such horrific tales about animal cruelty in Ireland. I lived abroad & there were some animals, but, I never heard stories about cruelty like the stories in Ireland. Are we a particular cruel race here to animals in Ireland?

MODULATOR:

I think we've learned, educated towards cruelty to animals as they are in other countries. I don't know, I think that a lot of european countrie's, mainland european countrie's would habe similar problems as we have. As far as the UK & America, would definitely be better.

PARTICIPANT 4:

My experience is with Scandanavia.

MODULATOR:

Well yeah. Yeah ok. Well the Scandanavian's take it to a new level altogether. Like, there I think it's in Sweeden, you're dog is connected to your social security number. They're micro-chipped & your social security number is included on it.

PARTICIPANT 6:

they actually re-home a lot fo greyhounds & lurchers from here, in the Nordic countrie's.

PARTICIPANT 4:

I'm just starting to believe that the Irish are a particlarly cruel race to amimals. Unfortunately.

MODULATOR:

I think education is a big thing & I think our culture seems to be not to have that kind of pet. They're not considered pets by a lot of people yet.

PARTICIPANT 4:

Has there been any theories? Has there been any kind of analysis done on that?

MODULATOR:

Oh yeah, there's a lot of stuff going on. There's the other thing aswell, research is kind of young with us. Vetinary research aswell is quite young with us. There's a lot of tha going on. Thre's actually in my office, he was doing work on horses & looking at horses in shows & that particlar. And looking at cruelty in horses. So I mean that's the other thing as well as that, the finding isn't there as in other countrie's like the UK & Sweden & Finland where their culture is a lot more towards the pet. There's a lot more money in things like the R.S.P.C.A. So there's a lot more money there for funding research than there is here.

PARTICIPANT 4:

But I just, that there would be genuine shock in Scandanavia & these countrie's, concering animal cruelty. There doesn't seem to be that much shock in Ireland. And, I begin to wonder, was it an underlaying cultural thing here. I suppose it's a pretty drastic thing to say but, I wondered if it had something to do with our religion, that animals were such inferior beings compared to Gods *Participant 4*ointed.

MODULATOR:

God, I don't know.

PARTICIPANT 4:

I just wondered you know if it could have something to do with that the way we are so indoctrinated about our own importance with the world compared to animals, where Scandanavian's aren't, they don't have that kind of belief.

PARTICIPANT 6:

Well you prosecute somebody for cruelty to an animal & it takes maybe a year or more to get them into a court & it's all to do with the law. That end of it. It's to get it in to the legal heads that cruelty is an offence as such.

PARTICIPANT 4:

Precisely. They don't sort of feel it. They don't seem to feel that they should be.

PARTICIPANT 6:

A couple of years back we had a situaton with sheep & thes guys were prosecuted & it took over two years to get them in to court. They were always missing, out of the counrty every time the court came up & then they fined a pityful amount you know. And like if you're the witness or something, you're the one peppering abou waitng to get to court. Not the.....

MODULATOR:

Not the person who's doing it?

PARTICIPANT 6:

The fines & the punishment isn't enough. And then take Co. Wexford for instance, you've one dog warden to enforce the law on dogs in the whole county. Now he's brilliant. The man what he's doing.

PARTICIPANT 4:

Exactly. He'd have to be.

PARTICIPANT 6:

But how can he enforce over the whole county?

MODULATOR:

It's difficult alright.

As much as I'd love to discuss it for a very long time, it's just a little bit off the point, kinda.

PARTICIPANT 2 LEAVES

PARTICIPANT 6:

If you want to get back to spaying & neutering. I've everything spayed & neutered because I always get them done before they go in to their first heat if it's a female. At least, it will prevent them having infection or uterine cancer down the line or mammery cancers or things like that so that would be my feeling on getting them spayed & neutered. The male neuterd prevents them procreating &getting in to fights & passing diseaese on.

MODULATOR:

Yeah.

PARTICIPANT 4:

Although you don't want to discuss it too much further, I just wondered if he vets in general could sort of, make more of an issue of it.

MODULATOR:

In what way now?

PARTICIPANT 4:

Emphasise the, I suppose or even mention the facy that there is alot of animal cuelty in Ieland. And that if you are an animal lover you are considered to be some kind of maniac of some kind here in Ireland. An animal lover has had a bad name here in Ireland.

MODULATOR:

Yeah, I mean....

PARTICIPANT 4:

The people who demonstrate at hunts here have a very bad name. People who are opposed to hare couring have a very bad name. Not the other way around. I find that astonishing. I find that astonishing. It would only be in Ireland.

MODULATOR:

Yeah, well I mean.... I suppose the problem is that there are two groups that get involved in that kind of thing. You have the activist activists, who cause problems, & the animal fanatic who goes out & causes trouble & I think, kind of like being tarnished with the one brush & that's why those people who are anmal lovers, but the people who are....

PARTICIPANT 4:

I do't see, I've never read, I have never seen anything that's drastic that they've done, the demonstrators you know.

MODULATOR:

I don't think we see that much demonstrating here though at these kind of things.

PARTICIPANT 6:

There were a few years.

PARTICIPANT 4:

I often thought about if I did turn Turk, I'd probably join those animal rights activists groups.

MODULATOR:

I suppose one of the biggest problems, as *Participant 6* says, there is very little support in the Government & there's a very good group at the moment. They're ANVIL. I don't know if you've heard of them. They are animal, basically I can't think of the full name, basically, it's anmial rights at this stage. They're involved in trying to get.....

PARTICIPANT 4:

What's the name again?

MODULATOR:

ANVIL as in ANVIL....

PARTICIPANT 6:

ANVIL. A....N...V....I....L...

PARTICIPANT 4:

Anvil as in anvil?

MODULATOR:

There's a good web site, I think it's anvil.ie or anvil.org. They're very good, like I've been talking to Miriam about re-homing issues.

PARTICIPANT 4:

I must look them up. *Minister for the Environment* was saying, oh no, it's only the stag hunting. There's only stag huntimg in Meath anyway that they were opposing & going to pass legislation on. Not hare coursing or this or that, why not? I mean, why not?

MODULATOR:

It's....yeah...

PARTICIPANT 4:

I find that appauling.

PARTICIPANT 6:

It's all to do with politics.

MODULATOR:

It is, I mean all to do with politics & it takes, you know, it's like trying to build a road, it takes so long for that knid of thing to happen.

PARTICIPANT 4:

But people can look on that kind of thing as sport!

MODULATOR:

Yeah, I know what you mean.

PARTICIPANT 4:

I mean it's, it's, it's......uncivilised!

PARTICIPANT 6:

One big mistake *Minister for the Environment* made was he moved animal welfare from environment to agriculture.

MODULATOR:

But the whole animal thing is.....

PARTICIPANT 6:

He should have left it in environment. Animal wlfare as such.

MODULATOR:

well I think the problem is that there's so many people involved in it. Coz you have you say, the environment. The Department of Environment are involved in the control of dogs, the Department of Agriculture involved in welfare & in disease spread & then you have the department of, what you call it, can't think of it now. It covers the Guards...

PARTICIPANT 6:

Justice.

MODULATOR:

The Department of Justice are involved in the legal issues & here's no Government body involved with actually prosecuting people for animal cruelty. That is done by the I.S.P.C.A. So, the I.S.P.C.A. are given this legal right.

PARTICIPANT 4:

I tink it's all wrong. I think a new enlightenment is.....

MODULATOR:

I mean, that's what the whole ANVIL thing is about. Trying to do that.

PARTICIPANT 4:

I think a new civilisation could begin in Ireland with animals.... the treatment of animals.

PARTICIPANT 6:

ANVIL is a sort of umberella group of all the others.

MODULATOR:

Check out the ANVIL thing & see

PARTICIPANT 6:

It's an umberella group. Of all the animal welfare groups they come out of ANVIL.

MODULATOR:

Every body kind of supports it..... they're quite good & they were big sellers in getting the recent legislation about puppy farms & stuff like that.

PARTICIPANT 4:

That's a start.

MODULATOR:

Yeah it's a start but, I think it's all about doing things slowly where we're concerned, unfortunately.

PARTICIPANT 4:

I actually think it would be a great thing for Ireland if it were emphasised & taken seriously I think it would enforce people to rethink about animals & I think in general that we would become a more civilised society.

MODULATOR:

Yeah, yeah, but I think it's going to take a long time you know.

PARTICIPANT 6:

There was a lady interviewed, I just heard a little bit of it this evening on the radio, when I was going from A to B & it was somebody from D.S.P.C.A. & I dont know what happened there was some situation in Dublin where a family pet had been cruelly treated. I don't know wheather a dog or a cat, I've a feeling it was a dog. Something dreadful happened about it. And the point that was made was the Gardai would have to get involved in this case & prosecute, because they maintained that young people that start out with cruelty to animals, usually end up in trouble later on. They are the ones that are attacking other people or murdering or whatever. They start with the cruelty to animals & they're going to esalate..... as she said it will be in the papers, it was only a dog, but as she said wait until the next is a human.

MODULATOR:

Yeah.

PARTICIPANT 6:

As at the dogs place, it should have been a prosecution. Should take place.

THE VET ENTERS

MODULATOR:

It's off the topic at the moment but, I mean it is always something that we're looking at. Welfare is very important.

But, I'm going to drag us back to the discusion. We can talk about it afterwards when we're finished. Ir's just that we'll be here all night if we keep going on that.

I suppose what I actually want to get from you is what influenced your reasons, like was there any TV programme or anything like that or what was your best influence in neutering your pet?

PARTICIPANT 4:

Concern for the pets. It wasn't anything more than that. Concern for them. To stop the tom from getting in to trouble with other peoples cats or females & bringing unwanted, with a female cat, unwanted kittens.

MODULATOR:

It's just you own experiences more so.

PARTICIPANT 6:

I remember going, even back in 70's, way back in the 70's. It was always there.... you did that, you didn't let them go in to heat. You made sure it was done & that went back to the dusty 70's, if not earlier. I

remember haveing them done way back. I remember Michael O Shea doing cats for me at one stage. It was sort of in the psychy you don't allow them to reproduce.

MODULATOR:

Yeah.

PARTICIPANT 6:

In later years now I've always had the dogs & cats done. Prevent disease & prevent other, you know....

PARTICIPANT 3:

I'm talking way back 40-45 years ago when you were a kid & you'd love to see aa cat with a litter of kittens or a dog with pups. As you say there was no tought to the animal welfare of them. They went out there & they were the bottom of the chain. There was cruelty that went on in families, never mind with animals. It was a great thing when neutering came in & education.

PARTICIPANT 6:

We're trying to get soebody in to the schools.

PARTICIPANT 4:

I can not remember anything in the media or on radio or television had influenced me on that issue anyway.

MODULATOR:

So where do you tink you got your information from? Was it just general knowledge or reading?

PARTICIPANT 4:

I think so, I don't think there was anything specific, I think it was just in general.

MODULATOR:

Alright so, I supposeyou want to take a break, get a cup of coffee, cup of tea, or do you want to keep on going?

PARTICIPANT 6:

I'm happy enough. Whatever you want to do.

TEA BREAK

WOLF CONVERSATION STARTS

PARTICIPANT 1:

Remember on the way to Dublin.

MODULATOR:

They were for sale yeah. I don't think they were real wolves though, I think they were a cross or hybrid.

PARTICIPANT 6:

Yeah, maybe a Shepard cross or something.

PARTICIPANT 4:

Are these real wolves you're talking about?

PARTICIPANT 1:

I didn't think there was any in Ireland.

PARTICIPANT 3:

Yeah, there are.

MODULATOR:

There are yeah.

PARTICIPANT 4:

A real wolf?

PARTICIPANT 6:

Yeah, they're real pets. They're only.....

MODULATOR:

They're just big dogs.

PARTICIPANT 1:

I suppose its a misconception sometimes the same with greyhounds. People think they need loads of exercise but they are actually big slobs. They love sitting around & you know they make really good pets.

PARTICIPANT 4:

You'd imagine a wolf would have such strong impulses, you know to hunt.

MODULATOR:

The same as another dog. Most dogs have......

PARTICIPANT 4:

They're domesticated more, aren't they?

PARTICIPANT 6:

Yeah but these sre domesticated now aswell.

PARTICIPANT 4:

Are they?

MODULATOR:

Yeah....

PARTICIPANT 1:

You'd imagine wolves as being vicious or something.

PARTICIPANT 6:

No no. They're very placid.

MODULATOR:

They are yeah. They are very similar to German Shepards.

PARTICIPANT 6:

Yeah very. They even look like them.

PARTICIPANT 4:

German Shepard is wolf like. People are always afraid, people you know are always dead afraid of them.

MODULATOR:

They can look like they'll take your head off.

I'm going to start us back again if thats alright. So we can go home at some stage.

What I'm gonna do now is pretend The vet isn't here & you can all say bad things about the vet if you want, thats's no problem.

THE VET:

You said there was no questions.

MODULATOR:

No there isn't, I'm only messing. Ah so what do you think of your vet? No, no. I'm only joking. What I want to talk about is pet diets. Wheather you use kind of a hoe made mix or wheather you feed scraps or wheather you use commercial foods & what you think about either home made diets or commercial

diets? Wheather your for or against different diets? Or if you think it's all a pile of hoo ha? Or wheather it's very important to feed proper diets? What's your views on that?

PARTICIPANT 1:

Well I use a mixture, like mostly the dry food, like the dry nuts dog food. Then we'd use a mixture, like of boiled chicken or mince for our dogs. Now for a treat, that wouldn't be every day, but for a treat. Something like mince or chicken that has protein in it. That & the dry food mostly. We don't use the wet dog food at all.

MODULATOR:

And why do you give your dogs the human food?

PARTICIPANT 1:

I suppose because it's a treat for the dogs & they love it. You can see by them they think it's a treat aswell. They really enjoy it. The same as if it was human getting a treat. Say something that you treat yourself to maybe once a week or once a fortnight. It's the same for the dog I think, they enjoy getting a treat & I suppose it's something different from their normal food routine aswell. Maybe to vary things a bit.

MODULATOR:

Anybody else got any views on that?

PARTICIPANT 6:

I use all dried nuts for the dogs & cats & water. Now the old, before she died needed medicines & stuff, so I used to boil chicken or turkey. She'd get rice & maybe a bit of weetabix for a bit of fibre. When I was giving her medicines.

MODULATOR:

Oh right. Yeah.

PARTICIPANT 6:

that was to help her get down whatever she needed. Other than that she always loved her nuts, her dog nuts.

MODULATOR:

So you never felt the need to feed her human food aswell?

PARTICIPANT 6:

No, we never. Other than when she was ill.

MODULATOR:

And what did you give her, kind of treats?

PARTICIPANT 6:

Yeah they all get.... it costs me a bomb, you know like the denti sticks. Especially when it comes to your getting them into the run. You get the bag & they fly in. Waiting. The two of them fly inthen waiting for the denti stick. And that's what they get. They get two or three of those. I always buy the small ones & then rather than the big ones & just give them the small ones. They get two or three of those a day. That's their treat for going into the run.

MODULATOR:

Right, ok, very good.

PARTICIPANT 3:

Well I have a bag of everything. I have dry food, but because maybe I would have one with a litter, I'd feed her raw mince. I also feed chicken coz if they were sick or anything like that. But, I have one that haas an allergy. My vet doesn't agree that it's high protein, but I do. So, when I feed her a lot of protein, she gets an itch, so I have to balance that out.

And then I had a problem with the teeth. I find with the dried food their teeth get very..... especially with Cavaliers, I have to do regular cleaning & I have to get them scaled. So if I could find an easy way of doing that now. My vet who I won't name, says I should be washing their teeth every night, but I said, I haven't time to wash my own. You know realistically I wouldn't be getting a toothbrush out.

MODULATOR:

Did you ask your vet, does she clean her own pets teeth?

PARTICIPANT 3:

No... You know if I could really find a lazy answer to cleaning teeth. Now, I know the Collie. She's great. She's fifteen or sixteen & her teeth are as good. Where the Cavalier I find their teeth get quite caked. If I could get an answer to that.

PARTICIPANT 6:

The denti sticks, did you try those?

PARTICIPANT 3:

I tell yoy what, I get those hide bones, but I really don't , because I find that when you have a few dogs, if you have them, I'd be afraid of them picking up infections on them. You know, if you're leaving things hanging around.

PARTICIPANT 6:

The denti sticks are not hard. They're quite soft, they eat them down like food.

PARTICIPANT 3:

Yeah.... & are they good though?

PARTICIPANT 6:

Well now when I lokk back at the old German Shepard,she was almost fourteen & all her teeth were perfect. Now she had a slight little bit of plaque on her two long ones before she died. That was about it. She ahd no plaque on the... I couldn't see any on the others. There was no brown on them. Just the two at the sides, you know.

PARTICIPANT 3:

But I wonder is that just with the big dog rather than small dogs. Because the Collie would kind of eat anything that was going over the years & got dry food. She was mainly fed on dry food & you know the way you'd have some meat to give her. I still give her now what I feed the Cavaliers. I give her raw mince & if I had a bit of chicken. Her teeth are as good today. But I have a problem with the little ones.

PARTICIPANT 1:

The Cavaliers, their teeth can go very discoloured sometimes, I notice that. You know at the side. Maybe not so much the front, but the sides go a bit discoloured I think.

PARTICIPANT 3:

Is that all Cavaliers then, do you know?

THE VET:

There are breeds that are definitely worse, like the Yorkies, & the Cavaliers would be, & as you say sometimes the smaller breeds. Not necessarily Terriers now, it...... Exactly, then as you say, you ca have an old sheep dog where you did nothing with them & their teeth are pearly white nearly.

PARTICIPANT 3:

Now a Jack Russell. My mother has a Jack Russell. He's a pet alright but he'd be given, he gets nearly, she'd give him her dinner some of time. And his teeth, sometimes I particlarly look at them, & I'd say to myself, is it only a cod. Are denti sticks & bones & stuff, are they any good?

Or even if you get down every night & washed your dogs teeth would it work?

MODULATOR:

It would. The gold standard

THE VET:

Yeah it would work just to wash their teeth but you use a dental toothpaste which is not foaming & obviously it's got a taste on it that the dog likes, so they actually kind of pretty much are up to having their teeth cleaned. Once they get in to the habit of it. But then if they're expecting you to do it, it's putting doggy pressureon!

MODULATOR:

I'll give you a handy tip later on. But I'm going to move on.

You say you give them some meat aswell. Is there any reason why?

PARTICIPANT 3: I give them meat because I actually read a programme one time, saying that a dogs natual diet is raw meat, vegetables & rice & I think their coats.... If I keep the protein down a dog actually will get back sturdier & her coat will be better with a little bit of mince or chicken. That's why I do it. Was there anything that you fed your dog?

PARTICIPANT 4:

I've two cats.

PARTICIPANT 3:

Yeah, but when you had the dog?

PARTICIPANT 4:

God, when I had the dog he ate everything. A German Shepard. I can't remember having ever to buy anything special for him to eat. He ate mostly what we ate to tell you the truth. And a bit of dry dog food. But dinner stuff, he'd eat what we'd eat.

MODULATOR:

And what about the cats?

PARTICIPANT 4:

Yeah the cats. One of them at the moment isn't eating too well so we're trying him with all kinds of things. But he is eating dry food & they always eat the dry food even if they go off certain sachet foods or chicken. I give them chicken, they like chicken under noraml circumstances, but they will go off certain types of sachet food, commercial sachet food. So I have to change them every so often, but the dry food they always eat.

MODULATOR:

Right.

THE VET:

I think it's a very personal thing isn't it. People & their dogs or their cats & you know what they feed them. For instance now when I bring my dog Bonnie in to see my father he immediately puts out a container on the floor & puts a big splash of milk into it for her. And it's a good job she comes in coz I'd

never give her a drop of milk. And this is what he gives out to for. Some people always have their particlar habits that they like. They might give them dry food but then always give them something else.

PARTICIPANT 3:

I always ask is it enough? Is a dry food enough? And I'd say everything is in the dried food.

THE VET:

Yes.

PARTICIPANT 3:

I give them sardines for calcium. Someone told me about that. They lose calcium, good for the bones.

PARTICIPANT 4:

Do they eat the sardines?

PARTICIPANT 3:

Oh they love it yeah. Love sardines.

MODULATOR:

Is that for when they're pregnant.

PARTICIPANT 3:

They lose calcium you see when they have their pups & I give them sardines, coz if you give them too much milk it makes them runny. You know I give milk with honey for a few days.

THE VET:

It's a good tip for cats too if they're not eating. Is to heat up the sardines, coz you know the way it fills the house with the smell of fish. You know if you ever cook mackeral you smell it nearly next door. It really intensifies the smell if you heat it up.

MODULATOR:

That's good. What's next?

So the next or the last part of it is going on from feeding & stuff, is about weight. The weight of your pet. What do you feel influences the weight of your pet? Is it the food or is it exercise & diet & combination of things? Any of you have any animals that are overweight? Do you think?

PARTICIPANT 1:

I'd say mine could shed a few pounds.

MODULATOR:

And why do you say he's like that *Participant 1*?

PARTICIPANT 1:

Well one of the Cavaliers, I suppose, it's their age really. Well no, the sister is not overweight. I suppose the dog I'm talking about,they were never particlarly energetic dog, compared to maybe the sister was a lot more energetic & maybe just lazy. They got the same diet as the other one, but she seems to be a bit on the overweight side & yet she doesn't want to go for long walks or anything. She'll come with you & then turn around. But, I think a healthy way to maintain it is a combination exercise & a good diet. I mean, they need to get enough exercise like humans & to be fed a balanced diet, a good diet. Definitely the exercise is important, I suppose for their mental health aswell. Like humans aswell. I suppose their environmentaswell, you know if they're badly treated you will see a dog thats not thriveing. If they're in a cruel environment thy're not going to maintain their weight. I think that's an important factor with maintaining a healthy weight aswell, they need to be treated well.

MODULATOR:

Well *Participant 6*?

PARTICIPANT 6:

I suppose a combination of diet & exercise. If they're content they're fine really. I notice cats now in the winter time some of them, not all of them, put on a lot of weight. Some of them are naturally skinny, but with fine shiney coats on them. But I notice now that some of them have put on a bit, I notice in the last few weeks, they've all lost it again. Well they're out all day & all night, coz they're on the hunt at the moment, so they've lost it again. I think it's maybe because they sleep so much in the winter time. I don't have any of them very overweight.

MODULATOR:

Do you have any underweight either?

PARTICIPANT 6:

No, I wouldn't think so. Well there's one little one, she looks very skinny but she's stunted in growth & she was a little feral thing, but she's been spayed now & she seems healthy even though she looks skinny & she's flying around.

MODULATOR:

How about the doggies?

PARTICIPANT 6:

Ahh, they're fine.

PARTICIPANT 3:

I think some of them are a bit like humans. Some of them you couldn't fatten them no matter what you gave them & there's others would just fatten if you looked at them. Simple as that. And, I've actually spoken to different people, even that have dogs & even cats & they said that they're fussy eaters & they always look as if they could do with a good feed, but they eat plenty & they're healthy. It's just the nature of......

MODULATOR:

The beast.

PARTICIPANT 3:

Some of them are overeaters & they'll hoover up anything that comes around. And they'd put on, you have to watch them, coz they'd eat everything going you know. What about your pets?

PARTICIPANT 4:

Cats exercise themselves, & one of them execises quite a bit. Now he isn't eating so well & he's lost a lot of weight. Not eating as much.

MODULATOR:

He's good exercising? Is it?

PARTICIPANT 4:

And he's exercising he's out. He's as interested in being out as he ever was, but he's just not eating as well as he was & he's lost a lot of weight. I think you're right that there is a difference in cats & dogs & animals, just as there is in humans as regarding food. Some of them will pile on the weight & others won't put on any weight at all. That's the impression I get.

PARTICIPANT 3:

Yeah coz I've had one & I tried different things & just couldn't put weight on it.

MODULATOR:

Yeah.

PARTICIPANT 3:

And she's healthy & fine & she's a pet & she's as fussy! You know she'd turn her nose up at anything unless she get's a tit bit in the hand.

PARTICIPANT 1:

Or the human food starts to look better sometimes, & they might turn their nose up at the dog food. I supposse, if they think the grass is greener on the other side, if they think the human food is nicer.

PARTICIPANT 6:

Oh yeah, yeah

PARTICIPANT 3:

Or they hear you scapping the plates.

PARTICIPANT 6:

I find sometimes the dog will get in for the cat nuts. They really wolf them.

PARTICIPANT 1:

Yeah it's a novelty for them.

MODULATOR:

That's true.

PARTICIPANT 6:

But they all come walking together. Well not all but most of them when you go with the dogs & you'll have two or three cats following you aswell. They all go for a trot.

MODULATOR:

And do you take her for reular exercise yourself *Participant 6*?

PARTICIPANT 6:

Yeah, well today I was working so I took them this morning. There's somebody at home now during the day & they'll take them out in the middle of the day & they get a run now again tonight. Well they have a big run that they have during the day. They're in a run as such. It's over 20 feet long so they're able to run around it literally.

MODULATOR:

An you walk them yourself? Take them out for a walk? Coz it's kind of leadin on to the next question.

It's kind of how important ,it is to both yourself & your pet. Do you feel your exercice is routine?

Kind of both mentally & phsyically wise? Or do you find anything about yourself even?

PARTICIPANT 6:

Oh yeah. Oh I benefit from walking anyway, & so do they. They love going for their walk. Even though I have to keep the big one, the Husky Shepard cross, on a lead all the time. You could never leave her free even going down the lane at the back or anything coz I'm afraid she..... If there's someone else with me & there's a few dogs they play around for a while down the fields & she'd probably come back, but I'm always warey. You see I don't know her history. You see I think she hardship & abuse as a guard dog for a while & things like that, so you have to watch her like a hawk & she has an awfl thing about men. She'll go for a man. Any strange person or a man that comes in until, I don't know what happened her.

PARTICIPANT 4:

Something must have.

PARTICIPANT 6:

So I'm just vey warey that she would get free, so I always keep her on the lead. But,she's free in the run & free at night when she comes into the house, but I wouldn't leave her loose. The little fella now.......

MODULATOR:

I'll give you a call so before I come visit!

PARTICIPANT 6:

She looks such a fine dog everyone goes over to the run & I'm always saying don't go too close. I'll take her out & then you can talk to her. I feel happier if she's out of it & on the lead.

MODULATOR:

Yeah yeah.

PARTICIPANT 6:

But it's just when you take on rescues like that, you don't know their history or what hardship they had so you have to be a bit guarded with them.

MODULATOR:

So you have a regular routine with them aswell?

PARTICIPANT 6:

Pretty well. Morning, middle of the day, an odd day that's missed & then evening again & then tats it more or less. We try to get them at least three. Somebody else might be doing........

Actually I took her in, is it three years ago, she was supposed to be re-homed from my place, but two weeks after she arrived I fell & broke my ankle.

MODULATOR:

Oh right.

PARTICIPANT 6:

So she never left! So she's micro-chipped now & spayed.

MODULATOR:

Good. She's part of the family now.

PARTICIPANT 6:

She was re-homed & brought back coz whoever had her couldn't manage her. Re-homed down to Waterford so she was brought back & the next stop was euthenasia. So, I said ok I'll try her & see. But that's over three years ago & she's stll there. But I am aware, like of, manageing her. I'd never let her go to a kennels or anything like that because I'd be afraid somebody mightn't be able to......

MODULATOR:

Manage or handle her as well as yourself.

PARTICIPANT 6:

I'm used to her you see. She'll do what I tell her.

MODULATOR:

Fair enough.

What about you *Participant 3*?

PARTICIPANT 3:

I just find routine with them. They make you get up a certain time & they know their sleeping time & you have to get back for them. They're no trouble when you have them in a routine but they need their exercise, yeah. They'll let you know anyway coz they won't stay quiet if you don't let them out & run. It kind of makes you get out & do it. It works both ways & I have an indoor dry run. Cavaliers don't like being wet. Yesterday morning I let them out in to the sheep field. You know the way they love picking up sheep droppings. I let her down for a minute & she was gone out into the wet dew & she was the very same as if you half washed her. That sort of thing. You have to keep them under control mainly because they will go to anyone & I'm always afraid they'll be taken on me. So I take them out on the lead. I have that extendable lead. I put one on a lead & the others will stay near so you can run them. You know a boy & a girl. Run up across & come back because they all stay together like a pack. That's what I do.

MODULATOR:

What about yourself? Do you spend any time exercising with the pussy cats?

PARTICIPANT 4:

I spent a lot of time exercising when I was a teenager coz I used to help out in a kennels & it was, I think, the most pleasurable job I had & I don't know why I didn't stick at it. Because it would have suited me more than what I eventually ended up doing sitting behind a desk. But with my own dog, I loved taking him for walks & he was great on the lead & I took him for miles & miles & miles with him. I just loved it. Walked & walked & walked.

MODULATOR:

What about the cat?

PARTICIPANT 4:

Brilliant exerise.

MODULATOR:

For yourself?

PARTICIPANT 4:

For myself. I just loved taking him out.

MODULATOR:

Do you spend anytime with the cat exercising him? Playing with him?

PARTICIPANT 4:

Ah I spend a lot of time petting him. Not so much playing with him. I tried that & they weren't interested & they were small & I bought toys they could play with & they weren't interested in that at all. None of it. But they like to be outside. I see them running around the garden ater imaginary things. Cats do that all the time I think.

MODULATOR:

U.F.O's

PARTICIPANT 4:

But if they see me in the garden sitting down, they're over & up on my knee immediately.

MODULATOR:

They like to be petted.

PARTICIPANT 4:

Oh yeah, oh yeah they love it.

THE VET:

They do give gerat satisfaction though even watching them hopping about.

PARTICIPANT 4:

They're so agile & alive & graceful. I love watching them moveing. I envy them.

PARTICIPANT 3:

Did you ever have to put a bell on them? Our one used to follow the birds & I used to put a bell on to let birds know.

PARTICIPANT 4:

I put a bell on...... for bird protection. I had to put one on with the male because he was going down to the neighbour & she had the bird food so low. It was a death trap!

MODULATOR:

Maybe she didn't like birds.

PARTICIPANT 4:

I wanted to go in, but I don't think she was the type of person that I cold have. I wanted to say oh please please can you hang the food up in the trees. And she had tress all around her. So yeah I had to put a bell on him & he went out & came back an hour later & the bell was gone.

THE VET:

That's a bit like Health & Safety going on......

PARTICIPANT 6:

It's amazing though how birds tolerate. I see what trees & with the old ruin of the house at the back of us. There's so many birds building in them. And they even try to build, the swallows are coming in just at the piece that goes out over the front door at the moment, & the cats are sitting on the front door step, & they're coming in to build. It's amazing how they all seem to tolerate. I have one guy,I took him in as an adult & I kept him inside for a while & then when I first brought him out, two blackbirds swooped down in front of us, at the old house. Right down, tipped him on the head, literally swooped over him. They were building, & he got such a shock he turned around & went back in. And I thought, now that's the best lesson you ever got & you know where homw is!

MODULATOR:

Brilliant.

PARTICIPANT 6:

And the same guy recently, he has done this twice on a neighbours car & once on my car. I was driving down, there's a small sanctuary near Clonmel & hey were taking kittens from me. Six kittens. And I was driving up to Ballyroebuck & just as I was going down the village tis car came up behind me & he was driving right up behind me & he was flashing the lights & I thought he was flashing at someone coming towards us. And he kept flashing & driving behind me. So I said I'd pull in & let him off be damned. The next thing, he pulls right up beside me & he says do you know you have a cat on your roof & just as he said it the ginger paw comes down. And it was my brave tom. This long haired ginger cat & I was well at the other side of the village & he'd held on. He did it another day & I was running like a lunatic coz I had visions when she'd swing out on to the road that he'd go the other way. Not a bit of fear of him.

MODULATOR:

Like something out of the movies.

PARTICIPANT 6:

Your man says to me, it was Miley Somers, he says to me you've got a stunt cat there doing that. I didn't know he was on the roof of the car at all.

MODULATOR:

Get a job for him.

PARTICIPANT 6:

But oh he's a fright. He goes into everybodys garden & I took him in as an adult. He was thrown out, sort of.

MODULATOR:

Right cat on the roof of cars.

PARTICIPANT 6:

And if he goes down the front towards the road, he won't go out on the road now, but we live well in off the road, anyone who's waliking up, he won't let them pass him. They have to carry him up. He'll trip them up. He'll keep tripping them up until they pick him up. Ah he's an old pet. He's not petted you know, he just....

MODULATOR:

Has a thing.

PARTICIPANT 6:

He's a long haired ginger fella & he just insists on being carried up from the front of the house.

PARTICIPANT 3:

Pets get so cute. So clever, & people who are not in to dogs or in to cats just don't understand what you're talking about.

PARTICIPANT 6:

When I saw that ginger paw coming down........(Laughing)

He didn't try to run away from the car. The paw just came down to come in the window. I just grabbed him, come in here quick!

MODULATOR:

Participant 1 what about you & exercising?

PARTICIPANT 1:

Ah yeah. I like walking myself anyway so I bring the dogs for longer walks than they probably want to go. Yeah I thinks it's a very important part for them to have a routine.. i think dogs get very excited about going for a walk because it's the main feature of their day. They're not going to work or whatever so their main thing for the day is going for a walk. So I think it's very important for them. If they don't get enough execise you can notice it maybe in their temprement. You'll notice they're not as contented as they shud be. You know it's important to have a routine for them & for them to be out & about. I would imagine they'd probably get depressed if they didn't get enough excercise. You'd notice it their personality.

PARTICIPANT 6:

It's stimulation for them, isn't it.

PARTICIPANT 1:

Like yeah, it's the main feature of their day. They don't have anything else to do.

PARTICIPANT 3:

My mother has a Jack Russell & he's that clever when you go in to see her, he jumps down from where he's perched on the seat beside her, goes out & grabs the lead & brings it over & gives it to you. Honset to God, even if it's only as far as the gate & back you have to bring him. That's why I say they're quite clever. And he's got in to the habit now so it's a bit of fun for him. He comes in & he makes you go for a walk.

PARTICIPANT 6:

But sure it's good for ourselves the walking you know.

PARTICIPANT 3:

I don't know, sometimes you could strangle him. He won't stay quiet until he gets the walk.

MODULATOR:

Very good.

You can continue talking about other things now. I'm finished with ye. Thanks for coming, it's been brilliant.

Location 3 Focus group 2

Modulator

The First mainpart is why do you own a pet? Why did you get a pet in the first place why did you get a dog or a cat in the first place?

Participant 1

Some of them are working animals some of them are pets, I like them they are great company, very affectionate, can sense if your in unwell or in a bad mode or whatever, just company.

Modulator

What about you *Participant 2*?

Participant 2

I don't, I suppose the same thing, well sure the same.

Modulator

Why did you get your dog *Participant 3*

Participant 3

Because he was in one of my favourite films when I was younger.

Modulator

So you just got her for company or what?

Participant 2

It was important that both dogs got on together, the other dog didn't like when he came in in the first place, he pinned him down, but there inseparable now.

Modulator

So you just have the 2? So why did you chose the springer?

Participant 2

Participant 3 chose that too, no particular reason, we got him at Kavanaghs, the first dog was a farm dog I suppose, there great company and all. tell you when anyone is there,

Participant 1

And the Smaller ones are the crosser ones. the wee terriers,

Modulator

Do any of you have any cats at home?

Participant 2

We do have cats, I don't really like them either, but they chase the ravens and crows away.

Modulator

So why did you chose the type of pet that you have?

Participant 1

When the children were younger, the first one we got was 22 years ago, we have her great grand daughter at this stage, we kept on the breed, and have little black and tan terriers, and when the market was I good I sold a couple of litters to the states actually a vet bought them on cuttingham island and they have faboulous homes, but there is no market for them at the moment, my daughter got a boxer and her daughter is at home, my daughter is in England now but the original boxer is at home but shell be the last one unfortunately, but em, and we have new zealand hunter, we got her for the sheep, but

again we never bred her, and she's 8 or 9 now, so we won't breed her either, the terriers I would breed if there was an outlet for them, the last littler I gave one away, and the last one went to england, through dunne deal and she got the dog, why she came to Ireland I don't know, but I was so pleased.

Modulator

Might have been cheaper maybe

Participant 1

I did make a few bob out of them, you know I wouldn't breed for the sake of breeding, I'd breed them once every 2 years, I wouldn't abuse them, but em, company, good watch dogs, I'd be lost without a dog, I go around talking to them the whole time, feal I'm loosing my marbles, (laughs)

Modulator

What I'm also interested is your fealings about neutering.

Participant 1

I don't agree with neutering, I think they get too fat and lazy and it's not hard to lock up a bitch for a month twice a year, I have dogs and bitches at home and I can cope with it so.. if you've a bitch in heat you just lock her up. I know dogs will ramble and and if they are not spayed maybe bitches too, but I don't agree with it, If I had to do it for medical reasons then I would

Modulator

What about you *Participant 2* what do you think

Participant 2

Well I'd be the same, their not neutered, the cats are neutered, we've 2 male dogs that are not neutered,

Modulator

Why do you neuter the cats and not the dogs?

Participant 2

I'm not allowed not neuter the cats, we'd be inundated with kittens, purely birth control.

Modulator

Are you particularly against neutering of dogs?

Participant 2

I've never neutered them, they get lazy, they haven't as much go in them,

Modulator

I suppose part of it is who influenced your decision to not neuter/ neuter the cats. Was there any particular influence on you?

Participant 1

No they just get fat and lazy,

Modulator

Have you experienced fat and lazy neutered dogs?

Participant 1

I've seen them, I've never had them before but I've seen them,

Modulator

What about you?

Participant 2

We've never had neutered dogs at all,

Modulator

Next thing seen as we're talking about fat and lazy, do you feed home made diets, or scraps from the table, or do you use commercial diets, what are your feelings about either or

Participant 1

Well I don't have any or enough scraps to feed 9 dogs, if there were scraps there I would use them, when the children were younger there was more left overs, they all got them, but I feeds nuts, and they're doing ok and are in good condition.

Modulator

Did you ever think about making your own?

Participant 1

No I've enough to do.

Modulator

So why do you use the comercial diet

Participant 1

Well I haven't got enough scraps and I don't like the canned food, the lactating and pregnant bitches get meat as well, but otherwise they get just the nuts, they're all in good conditions, with shiny coats, if there was any problems I would change the diet, I have no skin trouble

Modulator

What about you *Participant 2*?

Participant 2

We feed them greyhound 20, your not ment to give them chicken bones but charlie knocked them down and broke the plate and ate the chicken. and it didn't do him any harm to eat the chicken bones, I thought they were dangerous, but not in this case,

Modulator

I think some times you can be lucky, but often times not and it can be very serious (tells story). Do you use commercial diets or ... why did you chose greyhound 20?

Participant 2

Cheaper, charlie never looked as well, before when we used to give them canned stuff and bits of scraps and things, his health has improved.

Participant 3

When we got ours the breeder suggest we get the greyhound 25 for the dog

Modulator

The breeder suggested it?

Participant 2

The high protien stuff

Modulator

I suppose going on from that what do you think influences the weight of your pet?

Participant 1

I think it is the diet and the excercise, keep them well wormed, if they were getting skinny obviously I would be concerned, also if ther too fat, but we had a little bitch and she could just eat and eat and eat and eat, non stop eat, and she would just blow up and we had a job to keep the weight off of her, and her sisiter was skin and bones but em just like humans they all differ, some would eat more food than others some will life on sweet damn all. I keeped them wormed and if there getting skinny I'd worm them all again

Participant 2

Just once a year?

Participant 1

No no, maybe twice a, well twice a year anyway.

Participant 2

The thing on the back of the neck?

Participant 1

No I give them the Drontal + for the worms

Participant 2

How do you use that

Participant 1

In their mouth and its good

Participant 2

I just give them the stuff on the back of the neck, that does them

Participant 1

and that does worms as well?

Modulator

It does but it doesn't do tapeworms, ... so what about you *Participant 2* what do you think, do you think the same about the weight of your pets, are they alright or

Participant 2

Oh they're alright yeah, there not overweight, heavy enough.

Modulator

so what do you think *Participant 3*

Participant 3

em, I think they're alright,

Modulator

So what makes them alright, what are you doing right to make them alright

Participant 2

Plenty of water, bobby drinks heaps of water, he gets into pot holes and everything, he'd be teribly dirty,

Participant 1

The dirtier it is the better they like getting into it, oh stop, something dead or anything

Modulator

Do you have an excercise routine with them or anything

Participant 1

I let them out, not together would be rows, I'd let them out for 2 or 3 hours, depending what I'm doing, some of them are in prper kennels so they have the yard, I know thats not proper excercise you can see everything that is going on, but they're out running around the garden or up the yard, excercise Themselves.

Modulator

You don't take them out for walks or anything like that.

Participant 1

no I don't need to, the boxer plays football, she exercisses herself, no they just run arround, if they're out they hunt, any of them will go hunting, after birds rats, anything, rabbits whatever, and the'll run around the field, eat sheep shit and whatever

Modulator

And what about you do you take them out for walking or running or

Participant 2

We try to take them around the hills at the back, bobby though he lies around a lot he sleeps, snoaring like anything you know,

Participant 1

The dogs at home are not lazy, the only one who is is a bitch of 14 years and she's deaf so I don't mind her sleeping alot, but the others are very active.

Participant 2

Would that be a heart condition the snoaring?

Modulator

Not nessecarily, some dogs snoar... I suppose as well how benificial do you feel the time you spent excercising or watching them excercising is to you and your pet?

Participant 2

Oh it is for both alright

Participant 1

Man and beast

Participant 2

He likes going through the trees and all, he eats more, he runs and climbing up over stone ditches and things and briars as well, when you haven't got a park you know, there's a lot of briars,

Participant 1

Thats the thing, those little sticky balls from rubbing off the hedge or whatever it is

Modulator

And what about your own benefit, you said it was beneficial to both man and beast

Participant 2

Oh I think so yeah,

Participant 1

Keep the weight down, You couldn't walk on the road where I live because, jeases you'd be run over, I dont think, ah they don't need supervised excercise because when they're out they're running around, they're actually dangerous, caus they could hit you from behind, if you were mowing the lawn or anything, they'd be with you and run around and play so it suits me,

Modulator

Do you take yours for long walks or..

Participant 2

A couple of miles an then at night theyd get another 4 miles, and get a bit of excercise myself

Location 4

MODULATOR:

So basically what we want to know, the first question is to get you talking about your pets is, why you have a pet in the first place & probably generally people when they answer the first & second questions together. Why do you have a pet? & why did you choose the type of pet you have?

So does anyone want to to whip off? Here at the closest to my left hand side, Participant 1 ?

PARTICIPANT 1 :

Well there was always pets in our house growing up. So I love animals & to answer the second question. I have cats because I live in an apartment & I'm not allowed dogs.

MODULATOR:

You would have dogs then as well?

PARTICIPANT 1 :

Oh God yeah. I'd have everything. I'd have them all if I could.

MODULATOR:

Oh right, OK. Fair enough.

PARTICIPANT 1 :

We originally lived together. Its between us now.

MODULATOR:

Oh right.

PARTICIPANT 1 :

So we had two cats & a dog. She's in a house so she kept the dog. So she's.... I kept the cats.

MODULATOR:

OK.

PARTICIPANT 1 :

So that's pretty much it.

MODULATOR:

Very good. And so she answered your question then.

LAUGHING

PARTICIPANT 2:

Yeah, so it's the same thing yeah. It's my Mother..... she's cats, dogs, goldfish & everything living there & she's eight children as well so it's the whole thing of loads of activity & all the rest. So I've took the dog. The dog was here in the home so yeah. I love animals. They're cats as well but the dog doesn't take too well..... the cats that wee together she never touched them but so...... I'm stuck with just the dog now. And that's why I chose the dog, because I have a house. She took the cats & I took the dog.

MODULATOR:

Right.

PARTICIPANT 3:

I had four children. Three sons & a daughter. They're all animal lovers. I think dogs make a happy home. Small or big. I love bringing the dog for a walk. Seven walks a week. I like singing to the dog, I mean, I sing to the dog.....

MODULATOR:

So you get practise in then for your singing?

PARTICIPANT 3:

Yeah.... He has his party piece & so have I.

LAUGHING

PARTICIPANT 3:

He's thirteen & a half & he's been the best dog ever. I had five dogs & he'd go out in the garden & he wouldn't touch plants. He'd touch nothing indoors. I had a German Shepard & he would strip wallpaper when I'm out. Eat the skirting boards, ha ha, & he was brought for long walks too but he'd be bored very easily, the German Shepard, in the house, indoors. That's what I found.

MODULATOR:

So you have a smaller dog now. Is it?

PARTICIPANT 3:

I have an Australian Silky Yorkie;

MODULATOR:

Oh...

PARTICIPANT 3:

Eleven inches high & the vet said I certainly shrunk my preference from the German Shepard.

LAUGHING

PARTICIPANT 3:

He was rubbing the dog......

So I find a big difference with the different breeds.

MODULATOR:

Yeah...

PARTICIPANT 3:

You know I found the smaller dog is very clever. Where the smaller dog would get one over on the German Shepard, really I find, but... I love dogs. Every size, every colour & every breed & I'm not afraid of any size dog now. Boxers & all, I wouldn't be afraid of them. I'd bring them for a walk I would. You know, I would warm to the dog & the dog would get that. They'd get the vibes from me you know what I

mean. I wouldn't be nervous. I've seen people in the vet if a Boxer comes in & going like that you know & I wouldn't you know.

PARTICIPANT 4:

So em... I have a cat. I love animals. I wouldn't have initially thought of a cat, until a number of years ago a stray cat arrived in the garden. I would have preferred a dog I think. I like them all but I'd have preferred a dog. So I'm stuck with the cat & I now have a third. The third cat I own, he's ten years old. He's a Burman. He's an indoor cat. A complete indoor cat. The first time I've kept an indoor cat because I just used to have nightmares when the others would run out & run towards the road, so this fellow is totally indoors & seems to be thriving on it.

I have a pet because I love animals. I just love the bit of company. I think its great when you go in & there's a little furry creature to welcome you. I think they give a lot more than they get. I suppose the cat is ideal for me now that I've got a few health problems & I've got arthritis, so if I had a dog I wouldn't be great at walking him in the winter. So the cat turned out to be a perfect solution.

PARTICIPANT 5:

I suppose you're either an animal person or you aren't & my family have just always been animal people. Since we were kids we've always clamoured to have cats & dogs. At the moment I've two cats & my Mum she has a little dog of hers & she just has them because they're family & they're great entertainment & they're you know, some one to talk to & react back with you & they're just great fun to have around & energy & in particular for my Mother as well. I think they're great company for her when I'm not there. To have an excuse for her to get up in the mornings & feed them & go out & walk the dog &interact with the neighbours & stuff. I suppose it's just like extended family, but they're so unconditional as well like you know you can kind of talk to them about anything & you know they don't care like what you look like or whatever. You don't have to validate yourself in their eyes. They're just there constantly. Companions.

MODULATOR:

Does anybody have anything else to add or are ye happy enough with all that?

OK, so the next I suppose is kind of, I don't know you may all be neuterers. So I suppose it's what your kind of views on neutering dogs & cats? Whether they should be neutered? Or whether you think there are different decisions as to why to have your pet neutered or whether you think it's OK or not to have neutered. You know things like that. So it's to just kind of discuss neutering itself. What are the ideas?

Does anybody want to jump in on that?

PARTICIPANT 2:

Well I think they..... well.... yeah. Like we've always had all our pets neutered. You know she's after taking a kitten there, three weeks ago from the country. My Mother & Father have a house down there & like she does get the cats neutered as soon as possible. Like my Father has got four cats, strays down the country but he's going to get them neutered even though they're actually living in a field. Like you know what I mean. These cats have already had kittens & my Mum & Dad are that dedicated to the animals that they're actually going to get the stray cats from the field neutered.

Now..... I don't mean there's loads of them but like neutered is the way..... stray cats be neutered.... because it's not very fair the cats you know, especially the girl cats, I mean, she cats. They're practically attacked half the time with the Tom cats. You know what I mean.

Same as my girl Holly..... she's a....... she's a dog like & she's not neutered. The dogs tend to.....you know like.....just whatever. And it's just to stop all these stray dogs & cats & having babies & so I'm very highly..... I know some people say it's cruel but I don't think that it's...... it's worse to have animals put down coz they're strays, than to have them neutered, so I completely agree.... as soon as they possibly can, to have them neutered.

Because they don't..... what they never had they're not going to miss.

MODULATOR:

Oh yeah. Yeah.

PARTICIPANT 2:

You know what I'm saying. I know a lot of people have this attitude about a dog you know, oh God you have to let them....dogs have the........ females have a litter & all this, but, they never had it... & you know that sounds a bit like you know you're not giving them the choice but I honestly think that they're not going to miss what they never don't have.

MODULATOR:

Yeah....

PARTICIPANT 2:

Do you know what I mean like. Get them neutered from the beginning. They're not going to miss it that way. You know I....just like that kitten that what I have. That was one of the kittens down the country. They had that kitten & they abandoned it, because.... it was so young itself it wasn't able... They left the kitten at the door & my Mum & Dad saw it & let it in the house, you know, & then I went down & you know my Mum was.... oh the kitten's great!

NO IDEA WHATS BEING SAID HERE

ALL LAUGHING & TALKING OVER EACH OTHER

PARTICIPANT 2:

You know what I mean, even at that. My friends will leave those cats as they are. I'm telling you, they'll have a flock of cats there. They're down in Mullingar & I said to them catch them & I'll bring them with me & I'll get them neutered. At least they can do what they want but they'll still have the freedom. Let them outside & feed them & they'll be safe. Not having cats, like you know, more kittens like. They're like 100% now, Participant 2 totally.

PARTICIPANT 3:

Yeah I agree the same. The same. All the dogs they should be neutered, because there's an awful lot of strays & pups out there in the night time. You know, I don't think it's right. So if you get them neutered, there'd be less dogs roaming the streets & people putting them out. I know loads get a dog & have them for a few weeks & say they don't them & then that's bringing them to places like these. They just let them...... open the door & let them go. Put them on a train. I know people who put dogs on trains. Don't want them so there's..... em.... to minimise the amount of dogs I think it's good to have them neutered.

PARTICIPANT 4:

I absolutely agree. I had my cat neutered & I can see no point, unless you particularly want to breed from the animal for some reason. I think for the point of saving having unwanted puppies & kittens around the place & also the vets have pointed out many times, just to stop them going out & getting in to fights. I think particularly with cats, they get in to fights if they're not neutered so you end up with all sorts of injuries & infections & you're just putting them at risk too much. I wouldn't dream of having an UN-neutered pet. The first stray cat I had, I caught him & the first thing I did was brought him to the vet & I got him neutered & it's amazing how much easier he was to handle even after that. I think they reckoned he was about six years at the time.

You just don't... you hate to see.... with cats they get worse abuse I think than dogs & just see them being left under hedges & some of them are in terrible shape. So for health issues & to have the cat more comfortable for not to have anyone bother with it. I would agree to neuter & would agree with what she's saying in that so many people is saying, let it happen. One litter, but I know if you let them have one litter & you take away the kittens or the pups, that it's hard on them. I think it's unfair to do that because.......

PARTICIPANT 5:

For the sake of breeding them.

PARTICIPANT 4:

Yes. Exactly. So I think neuter all the way.

PARTICIPANT 5:

I'm absolutely adamant. Neutering is... if you take on the responsibility of having pets then i's you're obligation to neuter it & it makes me cross, people that don't because the amount of animals that are put down in Ireland because of......or find homes for are in their thousands & I think they're..... Even if you start feeding a stray cat I think it's you r responsibility to neuter, because otherwise they'll just breed & breed & it's not fair on the animal & there's such health consequences as well. You know they can pick up feline AIDS or do you know like with male Toms they're likely to stray & get in to accidents & get in to fights & stuff. So you just absolutely have to neuter them. You can't have a pet without doing it.

I don't know like, every one falls in love with puppies & kittens as well & you can see why they are drawn to them because they are very cute & even if you find homes for them you're taking away homes that would have gone to other animals. So I think absolutely you can't have a pet without neutering it & I think you should even have a license if you're going to bred animals just to make sure that you're responsible for your actions. That they don't get involved in the kind of pedigree puppy farms & all the rest. You know it should be brought in to the Law that all animals are chipped & neutered at their first opportunity, or else you get a license to breed them.

MODULATOR:

The second or third or sorry the fourth question there. So looking at kind of what influenced your decision in to having your pet neutered or not. So it's kind of looking at whether the media influenced you or you know the kind of original idea where you got the idea of about neutering your pet. Do you have any idea about what the influences were or was it just more common sense or......?

PARTICIPANT 1 :

Well I'd just grown up & that, since we kids & any animals we felt.... & that we said had animals for ever & they were always neutered at home, so it was automatic that we'd...... when I was going to take the cats & I said to Participant 2, we'll just get them neutered. Just something that was always taught at home. Because like with eight kids, there was dogs, cats, birds, fish & everything like so, do you know what I mean......

CAN'T HEAR WHATS BEING SAID

ALL TALKING OVER EACH OTHER

PARTICIPANT 1 :

No seriously since.... for every one of the animals that we brought to our house. Get it neutered. That's what parents...... in any way sort of..... that was parents who came from the country. So, I mean they had animals all their lives. You know like down....... they came from the...... the farm like, you know what I mean. They would have seen the animals & would have just thought, look we're not going to have hundreds of animals around the house. Get them neutered. So it's definitely a family influence.

PARTICIPANT 5:

Yeah coz the last cat my Mother took in was a stray like but she didn't know it was pregnant so she took it in. The cat & it had five kittens. Now my Mother has six cats now & it's in Dublin. The size of Catstown........ but she had all them in straight away. Now she couldn't actually throw away that cat & once she realised that cat & the kittens as well.... do you know that she's such an animal lover like she...... they're all indoor cats, but like she talks to the cat. She took that cat in for the kittens. Now there had been people who wouldn't have took the responsibility to neuter. Can you imagine the amount of kittens that would have come out of that so....

MODULATOR:

Yeah.... yeah.

PARTICIPANT 1 :

My parents said that they always had to neuter. Anyone..... I don't think I've ever heard of anyone that would disagree with neutering to be honest. If someone did you'd have a great argument with them. You know what I mean. I've never had someone disagree with me on neutering. It's always been

neutering. Anyone I've ever known has always said yeah go...... unless they're breeding. Which is another issue altogether.

MODULATOR:

Yeah.....

PARTICIPANT 3:

I notice in Dublin too mostly with dogs. Waiting until they get old because there's a vet bill & there's extra looking after too. You know what I mean. People I talk to, they say too much trouble, too much bother. You know that's what I find now. A lot of dogs when they're.....from nine upwards. I know people let their dogs out in the night time. Hoping they won't come back. I think that's wrong. Terrible.

MODULATOR:

Absolutely.

PARTICIPANT 3:

Yeah... the novelty wears off you see.

MODULATOR:

It does yeah...

PARTICIPANT 3:

The extra minding & the extra looking after & the extra coming to the vet to get them checked out & all. They don't want to bother. Well I say, what did you get one for in the first place? That all comes with it.

MODULATOR:

It does yeah definitely. And do you....

PARTICIPANT 3:

You look after them as long as you can.

MODULATOR:

And can you remember why you neutered your first pet?

PARTICIPANT 3:

Well all the dogs were queuing up outside the door.

LAUGHING

So & then...... I did hear that it quietens them down you know to get them down. To get them calmer. That's what I found with them. Where I had every dog in the neighbourhood you know. I couldn't leave the gate open.

MODULATOR:

Yes. Yes.

PARTICIPANT 4:

I really didn't want, with the first cat I really didn't want to see any more poor stray cats around the place & he was a male cat & I thought he's going to go back & do the business with some of the local cats so at least I can stop it from this side. That's something.....with the next two cats its a case of health issues & trying to keep them as well & out of fights. As you said avoid this strain that happens & the risk of Feline AIDS with them. They can pick up so much if they#re out & fighting. I think if they're not neutered they're wanting to be out more. Especially at night & that's putting them at risk from the traffic.

PARTICIPANT 5:

Yeah..... I suppose..... I mean it's always been a thing that's just keeping the numbers down. Like I'd have the house absolutely full of adopted stray cats & dogs but there's no point of bringing any more in you know that can't be homed or if your litters due, taking home's away from other animals. There's just an excuse in Ireland. There's more animals in Ireland than there are people to take care of them. So it's just not fair to them any more.

And then the health & safety issues are just a bonus like. If it was the opposite you know like if there were less......do you know.... if neutering didn't have the health & safety benefits but had a detrimental effect it would still be primarily there just to keep numbers down & making sure the animals they have are cared for.

MODULATOR:

Fair enough.

We'll move to a kind of totally different subject in a way. It's looking at pet diets, so kind of home made or commercial diets.

What do you feed your pets?

The next few questions are about your health & exercise & diet so......

We'll start this side Participant 5, this time. Keep you going while you're in the swing of it.

PARTICIPANT 5:

I guess they're on commercial diets. Veterinary recommended ones & it's just been important to us. The dog has got a liver dysfunction. So she's on a Patic diet. That's why I came up here tonight.

SOMEONE COMES IN

NO IDEA OF WHAT IS BEING SAID

PARTICIPANT 5:

Yeah & the cats on the veterinary medicated dry food as well & then occasionally we give them the wet food as a treat, but the dry food seems to be better for their health. So that's that.

MODULATOR:

Do they get any snacks or other treats & stuff?

PARTICIPANT 5:

Ah the snacks are just a way of getting the cats in & trying to stop them following me across the road. No that's all they get.

MODULATOR:

And you Participant 4?

PARTICIPANT 4:

I would be the same. The cats are on a dry diet. Again one that was recommended by the vet. He's a n indoor cat so I feel it's better for him to have an indoor diet to make sure that he doesn't get overweight & I would use the little pouches of wet food as well as treats. Again because I wanted to have something just for the sake of having something different. He's on heart tablets at the moment so he gets the tuna, the vet recommended that one & spring water. And he gets that with tablets just as a little treat. A reward for taking his tablets, he gets to eat a teaspoon of that. One in the morning & a teaspoon in the evening. The vets recommended the dry food right from the start saying it was better for their teeth & they know better than I do so. He seems perfectly happy on it.

MODULATOR:

Participant 3?

PARTICIPANT 3:

So my dog has arthritis & kidney problems & he's on a special diet. I do feel it's good for her. It's a low fat diet because if she doesn't get too many walks she'll get overweight. You know so the vet recommended the Hill's dry food. So I'm happy with that now. And now she's starting to walk faster than me on it......

LAUGHING

MODULATOR:

So yourself Participant 2?

PARTICIPANT 2:

So well that's a main problem..... the dog I have Holly...... now she's em...... she's very picky with her food & she's allergic to dairy. I think she's allergic to a lot more but it's just...... in the vets, not this time, but the last time...... because she was getting so skinny. So they tried putting her on dried food....but she.....if she doesn't like it, she won't eat it. She's not one of those dogs. But she won't. She just won't eat it. So I was like, oh God, I'll try putting her on some other kind of wet food. It's for like.....her stomach.....there's no.... meat.... there's no milk & all that type of thing. You know & whatever. She's very picky. Like what I do.... I tend to have a few problems wit her, food wise because even though I just feed her & that whatever, sometimes her stomach is still...... whatever...... that's another reason that she's here at the moment. She had X-rays & blood tests & all. She's fine anyway, but she can be a bit messy with her food but...... like whatever I'd give her..... the vet told me to give her boiled chicken as a treat, you know whatever, that type of thing, so she'd get that the odd time so. Go through this with her at times so....

MODULATOR:

Maybe Participant 1 ?

PARTICIPANT 1 :

My cats if I cooked would be overweight, but they're very skinny.....

LAUGHING HERE AGAIN

NO IDEA WHAT IS BEING SAID

PARTICIPANT 1 :

I think we got them out in Artane vets. You know when they used to put on....... And they gave us two kittens & that's grand. There's one just fluffy one & one just shored. But I had one of them, the shored fella & he ate all the food & the other fella got nothing so I tried them on the dry stuff.......having you know what I mean....... the other fella just got everything so...... well they're seven years of age now & it's gone to the stage now where the other fella, he's OK like.... the skinnier fella is getting his bit of food down but he's getting you know as much as the other cats & I don't know what to do with him like & I came to the vet & he said your cat is overweight & I said but what am I suppose to do like. Separate them, but I can't separate them because the skinny fella, he's not the skinny fella any more, he's not underweight any more, it's just like you'd pick him up in one hand........

LAUGHING

PARTICIPANT 1 :

No he's not like obese like where he can barely walk, but he's got like..... you know what I mean.... & he's just gone to the stage now I find it's the skinny cat loves milk. So I make sure he gets a lot of milk & he eats a lot of dry food, as the other fella he eats everything like you know. So it just got.... oh a disaster with them & like the kittens scoffs everything. Jesus anything that you'd leave out in the house. So I have to try to keep her away.... she's a she like.... her away from their food so. You know they have their moments when they're deciding on the wet food. So that's fine, I said I'd give them.... his two days dry food & when the wet food comes out again they're jumping..... they're cats..... they're unbelievable. Fussy.

TALKING OVER EACH OTHER

PARTICIPANT 1 :

Maybe that's just the gene in the cats. One fat one & one skinny one.

MODULATOR:

It's on a commercial diet is it?

PARTICIPANT 1 :

Yeah it's on..... & sometimes I would give them chicken. Now I wouldn't do them a full breast of chicken each like.... I just.... I'd make.... like if I'm having tuna & stuff I'd... you know, I'd have half a tin & I'd give them a bit then as a treat sort of & the I wouldn't..... if I gave that then I would also give them their food & I wouldn't overfeed them you know. I'd just keep them happy with that. I'd always make sure if I treat them to...... keep that as their evening meal or whatever. Well, no they're alright on their food but I tell you.... Jesus.....

LAUGHING

PARTICIPANT 1 :

Unbelievable. Seven years on they're healthy enough like so. You know.

MODULATOR:

That's good.

And you've brought us on lovely to the next point Participant 1 .

Which is what do you feel has influenced the weight of you pet?

PARTICIPANT 1 :

Well that's greediness.

MODULATOR:

Greediness?

PARTICIPANT 1 :

No honestly like when I'm picking them you know, like say if I'm away for a weekend. I might drop them at Participant 2 s house. I'd leave them with Participant 2 & I came to pick up the two yokes & one of them is like.....

LOTS OF LAUGHING

DON'T KNOW WHAT IS BEING SAID

PARTICIPANT 1 :

I was actually mortified last week end because they both had colds & I came out & I said Oh God.... where's that gone.... so I put them in the car but...... the cold you know the vet says it's not going to kill the cat, it's not.....he's not obese, but try & get the other fella a bit of food like..... you have to be there you know. It's unbelievable you know, the other fella like he's happy enough like, he's not....you know. He's happy with the milk & the bits of food you know. He's not starving by any means. It's just that the other fella tends to double eat what he eats. You know what I mean like but..... No he's greedy. The other fella is happy just to have a bit & the other fella he's just a pig. I know he's a cat but like he like ohhhh....

LAUGHING

PARTICIPANT 1 :

It's unbelievable like but, like I said animals they just...... they really are.....they've got their own personalities. Unbelievable & when you see these.......like one of them as I said......where as the skinny fella maybe tends to jump around & run around like, where the other fella says no I couldn't be bothered. You know, he's like yeah I'll sit on your lap all day. You can see the different personalities in them. But yeah a greedy cat.

MODULATOR:

What about yours Participant 2?

PARTICIPANT 2:

My dog is so fussy with her food. As Participant 1 was saying its about animals being complete individual.... it's like people.....she's just so..... I mean she was neglected & I took her in as a stray so would assume that she'd either, eaten anything..... now obviously with the milk.... I mean the dairy.... it cuts a lot of stuff. I can't give her..... as if like I could eat.... I try to feed her as I can home made, you know what I mean..... but I got a book & with all dog recipes. But everything in it involves some kind of dairy or something that I shouldn't be giving her so..... she, like the last time I had her here. She was here last week. She was sick, she was very underweight & I said God it's like two days since she got a bit better. So I mean, she's still underweight coz she's so fussy with her food so.... it's a bit of a nightmare now but... You see I could give her all of the crap that can put on weight on her but it's not good for her so.....

MODULATOR:

Yeah...

PARTICIPANT 2:

So I just so, you know what I mean, to just give her anything that she wants, I can't do it for her own kind of stomach sake, you know. All that, but she would be underweight coz she's so fussy & because of what I need to give her. So it's a bit of a nightmare.

MODULATOR:

So she'd attack the chicken curry without any problems?

PARTICIPANT 2:

She'd eat everything she can't eat. She's there looking at you & you feel so sorry for her. Looking at me. Everything she can't have & it's terrible because..... you know some people..... you know if she could eat what I'm eating like any other dog she'd be very healthy.... but she's healthy & great compared to..... today she's fine. A little underweight but what we thought was wrong with her spine but..... If I could hammer a bit of weight on her I could but.....so picky as it is & what she needs to eat she won't eat so..... it's a nightmare but you have to do it so......

MODULATOR:

What about you Participant 3, what influences your pets weight do you think?

PARTICIPANT 3:

I don't give D'Arcy a lot of treats, but if I have visitors they call him the hoover, because he's going around you know. He's not fussy what he eats now but a great dog to feed because no matter what you put down he would eat. But because of the liver & his health problems now I have him on a special diet which he loves.

Maybe once a month I'll give him jelly & ice cream. He'll only get it once a month. Where my husband wants it everyday, & sometimes if I'm in the sitting room & my husband is watching the telly upstairs, you know he'll say to me, how many visitors have we got? & I’ll say none why? Well you've been talking to the dog for two hours.

But I do think dogs, they know what you're thinking. Ahhh they absolutely do.

MODULATOR:

So is D'Arcy overweight or is he.....?

PARTICIPANT 3:

No. he's not overweight no. he's perfect.

MODULATOR:

Good.

PARTICIPANT 3:

Perfect weight, because of the arthritis I watch him you know, watch him, because he was saying he wouldn't be able to get up if you give him too many treats & I overfed him you know. He wouldn't be able........ Now my husband can't get up off the chair because he's over fed, but I wouldn't do it to the dog.

LAUGHING

PARTICIPANT 3:

No slim & trim the dog. Slim & trim. He's thirteen & a half you know. He has seven walks a week.

MODULATOR:

Good.

PARTICIPANT 3:

Yeah absolutely.

MODULATOR:

Keeps you on your toes then.

PARTICIPANT 4:

The cat, I think the diet is one of the most important factors in keeping the weight ideal for..... I'm told his weight is very..... he certainly looks..... because the long hair, he looks much heavier than what he really is. But he's the ideal weight. Because he's indoors all the time, I think that it's important that you play with him & give him a bit of exercise. He would tend to be lazy if he got a chance, but I think it's all the more important now hat he has the Cardio Myopathy, to watch the weight. Like all cats they love to guilt trip you. They see you at the fridge or anything so..... you know a little treat from the pouch is a teaspoon at a time. Oh but he would, he would guilt trip until you'd get something, then he'd walk away & say No, I just wanted to get it off your plate. So I mean the diet would probably be the most important thing to keep the weight as it is.

PARTICIPANT 5:

…... The dog has as I said a liver disorder so she gets a measured dose of a Hepatic dog food through the day & even if she gets treats, it's out of her ration, so she doesn't eat too much, but she's put on the proper medical weight since we got her because she was very skinny & as well I don't want her to get

overweight because she's arthritic so..... I suppose she gets her two little walks a day but she won't walk very far because she's quite stiff & sore.

And then the cats, they can graze from the dried food & then I give them just treats of wet food, but one of the cats was very underweight with a thyroid & she's thriving now, she's on medication twice a day & the other cat was, she's the runt of the litter when I got her so she was a tiny little thing you know. But she seems to just eat the right amount now & she's just healthy & happy & well so.

MODULATOR:

Right.

PARTICIPANT 5:

Yeah so just the right weight. Oh & I get them checked all the time as well. Bring them in for little visits, making sure their doing well. If they're medication has to be adjusted.

MODULATOR:

So I suppose the next two kind of questions are linked to this. So it's kind of how important do you think an exercise routine is? And how important to you?

So how important to your pet do you think the exercise routine is?

And how important to you, to spend that time with them exercising & playing with them, is to yourself?

PARTICIPANT 3:

The most important thing is definitely exercise. One of the most important things absolutely.

MODULATOR:

And do you think you get benefits yourself from it or.....?

PARTICIPANT 3:

Oh yes. Absolutely. Yeah. Yeah

PARTICIPANT 1 :

Well my.... my apartment.... I know it's an apartment but it's..... the balcony goes all the way around, so literally when I get home from work, the cats go out & run up & down & then obviously I'd spend a whole day.....(NOT SURE WHAT IS SAID HERE).... hey stay in the room with me as well & I know that I shouldn't coz... .. I always say no..... let you in tonight & then I'll stop it after today..... you know like if I'm going out for the night or I'm away for a weekend, I go, oh I feel bad. I'll let them in & then thing's like that you know.

Sometimes they would.... they'd chase each other as well & the little kitten now, she'd be chasing them too, the cats are all what's going on like, but now they're just like that. But the big cats, the little kitten tends to sneak up on them, but it's grand. They get the bit of fresh air outside on the balcony & the whole run around thing as well. Then I keep the doors open in the..... it's a three bedroom apartment so it has loads of room like. So they get enough exercise & the I try & spend time with them every evening, like they'd..... the three of them would be crowding around me basically. You know, one's one side & one's the other side you know what I mean. So I find now spending time with them is very important coz like you know they appreciate you..... & I love coming home coming along.......as you say, I love coming home & the cats are there. I know with a dog obviously they're more...... they're that more friendly & all but I just find that..... you know I walk in & the cats are just looking as if, yeah you're home, come on feed me now. They acknowledge you like & I know I mean.... people often say to me like you know, I can't believe you've got cats, like you know you're an old one. You know all this.... you know what I mean. And look I say it's better than coming home to an empty house. Simple as that like. You know at the end of the day, the cats they don't effect anybody else they're.....I'm happy with them. Anyone I meet, they're not happy with them, fine. Get over it you know.

But I find the company is brilliant. Cats are that bit easier then as well, that I don't have to stick a lead on to walk them. They're more than happy running around the balcony & I'd leave them out for a couple of hours, let them run out there & then at night time. Now they'll come in & they'll settle down on the couch & that's it. A few times I let them in the rooms as well now. Because they're indoors, I don't have to worry about them. Obviously you can't tie them up like so they're not getting..... they're not running through grass or anything like that, so I'm happy enough to let them in the room. You know like a lot of the times, you know they'll sleep like under the bed as well. Like you know. But look, at the end of the day it's my house. I don't care.

LAUGHING HERE

PARTICIPANT 1 :

I love them in, they're great company & I said I spend.... time playing with them is very important & they appreciate you as well. So that's me.

PARTICIPANT 3:

And it's surprising for the dog when you bring them for a walk everyday, they go their own route , right so, I back a horse in the bookies & the dog stands outside the bookies across the road, waiting for me to cross the road to go & back the horse right & if I don't go the dog seems a bit put out. Saying are you not going in to back the horse.

MODULATOR:

Right.

PARTICIPANT 3:

You know coz he wants to cross the over to go in, & they say to me in the bookies. We seen our dog Participant 3 & it wouldn't let you walk by. It wants you to cross over. They get so used to routine. Yeah yeah, absolutely now.

PARTICIPANT 4:

They are totally creatures of routine they are. You feel great if you spend time with them. So apart from needing to spend time with them for for your own piece of mind, I think it just helps you unwind when you come in. I think if you're playing with them, you well notice if there's any little thing wrong with them, you know you notice if they're out of sorts, because particularly with cats you'll probably find that they're so.... they're the sort of animal they don't show it as obvious as with a dog if it's ill it seems more obvious, but with the cat they can hide it. So if you're plying with them you'll notice that they're not as well as they were before. There's just a reluctance to play & again run them around the house. Keep the doors open. I don't know if yours are like that, I find that he has his times, he likes to play & he almost goes through a crazy hour in the evening. A certain...

PARTICIPANT 1 :

Yeah... I go straight to the door & he goes straight to the..... when I come in they'll run around like & then they'll go to the balcony doors, knowing, so you open the door....... so let's say they're in the house & I'm...... well at the end of the day......well I hope they're not going to jump off, Jesus they haven't so far but..... they run around & they just tend to run & they'll actually come in when they want to come in, I don't even have to get.... sometimes I do now, depends on if they're really hyper you know like they can be very hyper sometimes. But they come in like & say yeah I'm going to sit down now. And they'll sleep through the night. I know, that sounds like a baby but you know.

LAUGHING

PARTICIPANT 1 :

So when I go to bed, they go to bed & they won't wake up. I found years ago when they were younger they'd scratch at the door, you know like, hello, feed me like. This would be a t five o'clock in the morning, & I'm like no chance. You know like. Where now they just tend to , when I get up, grand OK I

accept I'm getting my food now. They're seven years of age so they just....... they have accepted how I am now. You know what I mean. They just go..... They're great, they're great now. Let them in & that's me. Easy for me.

PARTICIPANT 2:

…. Honey now she...... now she actually goes..... like you pick up the lead like..... we were away last week, well two weeks ago, we were away in Spain & my boyfriends parents took the dog & it's funny like, they're both deaf. Both his parents so like, so it was completely different for her. So she....the things that is mother was saying to me like after I brought her out. She says, she knows this & she knows that, & I'm saying yeah it's exactly the same.

You pick up the lead & she goes absolutely beserk like as if..... You put on your shoes & she knows she's going for a walk but she like......we make her walk, we make it really exciting for her so that it's not just a real bore walking, like there's a big park across from us so we walk her there like but.....spending time with her as well &.... before I....

It's funny how people that they tell you no I don't like cats & they tell you they don't like dogs. Like my boyfriend before he moved in, had a couple of animals in the house, nothing major like you know & he was like I don't mind a dog or... cats eeerrrr I don't like cats & I said well you're going to have to because she hadn't got her apartment yet. We're going to have to do the cats for a couple of weeks or months. And now, the cats, oh they're the best thing on the planet..... oh he absolutely.... or if we were heading over there. He'd take them or..... he gets excited about the cats.... he thinks the dog is the best thing ever. This is someone who had no interest in animals a year & a half ago & now he's ringing me three times a day. How's the dog? Is she all right? & I say yeah she's fine....

LAUGHING

PARTICIPANT 2:

And now his parents last week. When they took her for the week. His Mother was like heading away on holidays herself the day we came back. She was crying & wanted the dog back & she only had her for a week like. Obviously they're both retired & being deaf & all like.... she was such company to them. But they had that dog for one week & the Mother was crying. She was on a cruise & she was crying about the dog.

LAUGHING

PARTICIPANT 2:

…..texting the dog. I'm feeling very sad & we couldn't tell her she was sick & we couldn't say the dog's after being sick because she would have like.....Oh my God.....

MODULATOR:

She'd have wanted to come home.

LAUGHING

PARTICIPANT 2:

…...it just shows how someone can have an animal for literally a week end, ho much they change their opinion.....It's how much the animal gives you.... that like whatever.... you spend time it shows like......but the joy that she brought to his parents in one week & the way he feels about those animals now because of spending time with them

it's like having kids, obviously I know kids aren't animals, but if you don't have kids your animals are your kids, do you know what I mean. And like.... like he'd ring me about three times...... & I'd say she's OK she's OK relax, she's OK & it's funny how he's acting as if she's a child. You know what I mean so....

MODULATOR:

Yeah.

PARTICIPANT 2:

It's just like when you bring her for a walk, she just gets so excited & so happy &.... this is how I know I if she's sick & feeling & a bit down as you say you do know. She was very bad last week, but no matter what, when you bring her for a walk she's always, always, always happy no matter how much pain she was in. when she goes out for a walk she's happy. She's just absolutely delighted when she goes out for a walk so. Exercise is very very important, you know like some dogs won't....you know, some people say you know the dog won't go out the door. I don't understand that coz she's just mad to get out & then spending time, it's just how much...... like I say if you take the dog from someone or the cat, then you realise, I love having that animal with me. So spending time as well as the exercise. They're the two most..... they would be the two most things to be quite honest. Like fair enough the food is & all that kind of thing, but you can feed an animal, but you can just leave it. Like you can be someone who has animals, lets say you have a Chiwowa, one of those type dogs, but unless you walk them or you exercise them.....you exercise them & you spend time with them. The animal's not going to be happy & you can give the best food & give it whatever else but......the playing & the exercise personally they'd be the most important things, & especially to keep her.... when you see how old animals can get if they're that way, when people say, I had my dog for sixteen years. You know why because.....or there's loads of kids in the house or other animals there or....

A woman came in to me this morning with her dog to me, she was actually on the way out when I was coming back like & I was afraid the dog was all bleeding you know, like the way she was talking........?......

The kids are always giving out to me for the way I treat the dog. They're like your babies aren't they. Her dogs was her absolutely her entire everything. Obviously her kids had grown up & gone. It's great you know what I mean but it's all about the access you know. You'll hear her saying we'll go for a walk.....

CAN NOT MAKE OUT WHATS BEING SAID HERE

PARTICIPANT 2:

This woman, that dog was everything to her. Now it is great, especially when someone's older to watch how much company an animal is like you know. When especially her kids are gone &......like even for us how anyone ever says mean, I don't like cats or whatever. I'm telling you, get a cat for a week. Get a cat for a week & I tell you, you'll love it & you would. You'd just love the cat, don't get a kitten though coz they're a bit hyper but.......

A LOT OF LAUGHING HERE

CAN NOT HEAR WHAT IS BEING SAID

PARTICIPANT 2:

......I'm just thinking, was your cat like that..... the thing was climbing my leg. But like I said I'd never..... My mam would say will I take them back now. No. I said, he's cat.....I'll look after that cat now. No & I know.... you know I've got that cat for fifteen years give or take a bit. I don't care if any other..... & I've another seven years & I'll have another five or six years with them. You know what I mean like. I'd never influence the thing....... take a couple & see what happens like you know.

It's like I have them for life & that's...... & I know maybe fifteen years from now, I'll probably get or have more animals....... Well I might have ten kids in ten years time. I don't know, but I like know animals will always be in my life, without a doubt. There's no way I wouldn't. Absolutely love them.

PARTICIPANT 5:

As far as exercise goes, the two cats have access to the outdoors & they just kind of look after themselves like really. Like the older cat is very sedentary now she's nearly blind & you know kind of totters around the house & sleeps & doesn't really exercise, & the other one is quite bonkers. You know she chases the dog, she chases us, chases the woman across the road. Like it's really ridiculous. But yeah she's fit & healthy & you know she exercises herself. Yeah. And then the dog is a little bit doddery due to the arthritis, so she gets her two walks everyday, but she's able to walk less & less now & it's a real shame. You know it's part of my Mum's routine to take her out & everybody knows my Mum with the dog, & they'll stop & chat to both of them.

When I was out in Louth the dog used to come over to the church with my Mum & they'd sit there both of them & they'd have their little pray. The dog would sit & be really quiet in the church & then go off on their routine & they're...... ah yeah, she's walking less at the moment which is a real shame, but you know they'll keep it up..... She seems to keep her strong like. Keep her legs supple & the muscles strong so....

PARTICIPANT 3:

How old is she?

PARTICIPANT 5:

Em...oh gosh, she's between eight & ten. She's a rescue dog, so we're not quite sure what age she was when she came to us. But she has gotten a little bit more kind of sedentary I suppose, but then like she'll come back & she'll run around the house & she's happy so & she still seems interested in it.

Like we bring her out & she's like, oh great, can't wait to go. Like she's waiting to go, & then when she's there she'll just stop. She won't move & you've got to coax her along. It's really embarrassing now. All the neighbours are kind of used to me going Come On, Come On......

LAUGHING

PARTICIPANT 3:

And people are saying to me, oh you're slowing down Participant 3.

MODULATOR:

That's very good.

Anybody anything else they want to add?

Tell you the topics that we covered or are ye happy enough?

PARTICIPANT 4:

I suppose with the playing they make it quite clear to you that now is the time I want to play with you. I mean they come looking for play. With most animals I think. If you were busy they'll remind you, you know. When you have a bit of time they point the way.

MODULATOR:

That's true actually yeah. They get a bit hyperactive when it's time to do something.

That's fair enough. Thank you very very much for your time. Sorry to drag some of you in off the street. But thanks a million for coming in.

END.

Location 5

MODULATOR:

……why did you choose that kind of pet as well? Kind of you know for your own sakes. And so *Participant 1* seeing the way you're the closest one to me. I'm going to pick on you first if you don't mind.

PARTICIPANT 1:

That's OK. Well I didn't think about it very consciously I liked the mother very much. She belonged to friend & I said if ever you're having another litter I'd like one of the puppies but I didn't think about it seriously & I got a phone call one day to know did I want the puppy or not & we want to know because she had picked the puppy out that I was to have but somebody had taken it when she was away to bring it to their own house. So I said I would & I'd like to see it anyway & to make along story short that's how I ended up having it. It was a bit unconscious in that sense but I'd had pets as a child. Rabbit's, a budgie & gold fish & so on.

MODULATOR:

Does anyone want to jump in there on top of *Participant 1*?

A WOMAN MUMBLES SOMETHING.

PARTICIPANT 2:

……. when we were kids we had dogs & cats you know it was just normal.

PARTICIPANT 3:

We went to England with a dog, a little Jack Russell & we spent.....

PARTICIPANT 2:

There was no quarantine then 1960. yeah

PARTICIPANT 3:

The little Jack Russell & we spent, how many, 5 years in England. Coming back, we couldn't take him back because he had to go in to quarantine for 6 months. He didn't have to go in to quarantine going over but coming back he would have. My brother said he'd have him, keep him. My other brother didn't like dogs. Not this brother, & he was making his way back down to, back to our old house, the Jack Russell, so he has to be put down, & I was after taking him to London then couldn't take him back unless I put him to quarantine for six months & I said oh he'll never be the same after so long.

So then I got another dog a Collie, a Lassie. No oh a cocker.......

PARTICIPANT 2:

A Cocker, a Cocker Spaniel....

PARTICIPANT 3:

He was ever so wicked. A big black Cocker.

MODULATOR:

And why do you think you got a pet in the first place?

PARTICIPANT 3:

The company.

MODULATOR:

The company

PARTICIPANT 3:

The company, & the first Yorkie I got. I got her for for my birthday....

PARTICIPANT 2:

Yeah well we didn't normally get Yorkies, it was usually a Collie or something like that. Although we were living in..... but when we got the Yorkie we stayed with the Yorkies all the time for some reason.

PARTICIPANT 3:

This is my fifth dog is it?

PARTICIPANT 2:

Yeah.... no it's the fourth, it's the fourth one......

MODULATOR:

So you think the company mainly is.....?

PARTICIPANT 3:

The company now & no hairs.

MODULATOR:

No hairs.

PARTICIPANT 3:

No hairs with the Yorkies. Company & to be able to bring them for a walk. Just company.

PARTICIPANT 2:

You wouldn't have a Yorkie for security.... (ha ha ha ha ha)

You'd need a bigger dog for that like, you know. Although the Yorkie does bark....

PARTICIPANT 3:

She's very good she's very alert. But she hasn't been well there so she hasn't. She's had a lump operated on so.....

NO IDEA WHAT SHE SAYS HERE

PARTICIPANT 3:

She's alright now. She's still got the stitches out.

MODULATOR :

Fair enough.

PARTICIPANT 3:

At that side she's fine. Right anyway....

MODULATOR:

That's not unusual.

NOT SURE WHATS BEING SAID HERE

PARTICIPANT 3:

………. I don't know how she got to swallow it. A big lump of rubber. It wasn't soft , it was hard…….well I know that she picked it up but.....

MODULATOR:

They find a way.

PARTICIPANT 3:

So that's the story.

MODULATOR :

So *Participant 4*, what about yourself?

PARTICIPANT 4:

Well when I was younger I always had animals, as a young kid I used to collect snails & grass hoppers & keep them & tad poles & then we had chickens & ducks & this is a tiny little semi detached terraced house, a little semi detached terraced house & then we moved to a flat & we couldn't have animals at all

so I kept fishes, gnewts & then I went college & I got *Participant 5*ards. So I've always had something like that.

Living in London, Portsmouth & around the country like that. Met an Irish woman, we got married & the first thing we did was we got a dog.

MODULATOR:

Very good.

PARTICIPANT 4:

The house without one now seems odd. We just had one dog. Then we moved house & we got another dog & had children. I think that animals are good for children as well. So then we added guinea pigs & we had a second child & she wanted a cat, so we got a cat as well, & then we had mice & hamsters. Yeah we've had all that. Then we got a tortoise. We've had all manner of things. A real menagerie. We look after all animals. We've still got another guinea pig & a hamster being looked after at the moment, so we keep that separate from the cat. The cat we have at the moment, is a feral cat that we just have adopted & we fed it & it comes in to the window now in the evenings. You know sleeps in the bedroom. Hops up on the bed, butts her in the head! So it's a ell adapted feral cat now.

We have two dogs now. Generally we try to keep one old & one young & one will exercise the other.

PARTICIPANT 3:

And how old is the tortoise?

PARTICIPANT 4:

The tortoise must be about two years. I had a tortoise when I was young as well.

PARTICIPANT 3:

Do you know Mrs Bong, Sheila Bong? She lives in the first road. You live in the second.

PARTICIPANT 4:

I probably do but……

PARTICIPANT 3:

She lives in the top & she has a tortoise.

PARTICIPANT 4:

Does she live next to Eda Dern?

PARTICIPANT 3:

She lives beside Mr Kenny.

PARTICIPANT 4:

I probably do…..

PARTICIPANT 3:

She has a tortoise for twelve or fourteen years now.

PARTICIPANT 4:

This is just a tiny thing. My daughter wanted a tortoise.

PARTICIPANT 3:

Ah now this is not to big now either. It's in the house in the winter time.

PARTICIPANT 4:

Well this one has to kept..... (I think there's somebody else coming now)

PARTICIPANT 5 ENTERS LATE

PARTICIPANT 3:

……. the children had a green dot tortoise as well.

PARTICIPANT 4:

Did they.

Well this one can't be kept out until he's about two or three years old I think.

PARTICIPANT 3:

Yes. Theirs was in the house alright all the time I think.

PARTICIPANT 4:

It's interesting you know.

I think our pets do well as well. Our guinea pig lived until seven or seven & a half. Except for one of the dogs that unfortunately died early.

PARTICIPANT 5:

So you could charge admission fees & guided tours....

PARTICIPANT 4:

Guide tours yeah.... we don't keep them as guard dogs. The noise. Just having two dogs is enough.

PARTICIPANT 3:

One is enough.

PARTICIPANT 5:

It's a deterrent.

PARTICIPANT 4:

They're pets you know. One's a Terrier, one's a Biddow Terrier. There's 57 varieties, but it's was found in a canal, in a box with its tail chopped off.

PARTICIPANT 3:

Oh that's wicked.... that's cruel, cruel....

PARTICIPANT 2:

Did you get him in the dog pound?

PARTICIPANT 4:

No no. the animal rescue woman comes round & says you know do you want this, as if you are gonna say no. When you hear what's happened to him you know.....

MODULATOR:

So how about you *Participant 5*?

PARTICIPANT 5:

Well we got a dog for our children. Our eldest daughter was just dog mad, absolutely dog mad & if one more child in the school came in to the school with a puppy her heart would be broken, because she knew everything. Had researched dogs whatever & we'd done the goldfish & the hamster route & even a robot dog. And I found myself attached to the robot dog.

When she was ten, I met somebody going around the park with a Bichon & I knew they had to give back their previous dog. So I said how come..... because their child had asthma....so I was asking how come they got a new dog. So she said they got nan allergic, maintenance free, well not quite maintenance free but.... so I got the name of the breeder from her. Because both my husband & daughter would be hay fever, allergy prone. So that's how we ended up with the dog & we've had him now for seven & a half years.

MODULATOR:

Basically what we are discussing at the moment is why do you have a pet? And why did you choose that kind of a pet? Be it cat, dog, hamsters, guinea pigs, tortoise. So just kind of basically why do you have a pet? Why a dog or a cat?

PARTICIPANT 6:

Well the reason is I always love all sorts of animals. I have two dogs & two cats. I always ad them when I was a child. My mama, well we used to have a small terrier & she was never spayed, & she had puppies pretty much every time she could basically so we tried to look for homes for all of them. We were lucky that she never had more than two so we were lucky. But seeing that as a child I like animals.

I got a dog that I brought from Spain. Basically his mum had died as they were puppies & my father had found himself & a sister. So my father was feeding them for a while & then I convinced my mum to take both. But my mum couldn't really mind the two of them so I ended up keeping the male & my mum kept the female. So I brought him here to Ireland & then my partner went to a vet clinic where he used to work & there was this German Shepard that they were going to put down because he was a stray & he had a broken leg & so again I convinced him to keep the German Shepard. We did the surgery in the leg & we kept her. So that's the two dogs & then again I convince my partner to get two cats. So I went to a pound & I rang them quite a lot of times & they never answered the phone so after a while I talked to friend of mine who told me about a farm in Carlow where they had kittens & that hey wee looking for a home so we drove there & picked up the two worst of them! So that's what we did.

MODULATOR:

Right.

And does anybody have anything that has cropped up that they want to... you know kind of jump in every now & then if you have anything else you want to say.

PARTICIPANT 1:

Well the reason why I have a second dog is that I found the second dog across the road from my house. A little Jack Russell. She was all scrunched up, missing hair, skin flaking. She was all distorted in her shape. She was a terrible mess & I knew she needed a vet rather than a kennels, & my dog was going to kennels that night as I was going down the country the next day. So I left her down, it was seven o'clock in evening, I left her down to Dick Lavels place, before half seven, coz that's when it closes, & I collected her on the Saturday morning & I decided to keep her. I just found her very attractive & she gradually improved & she's wonderful now. Five years & a bit later. So that's why I have a second one.

PARTICIPANT 2:

As well in a family like, you can find that some people in the family love animals. A dog we'll say for instance, & a brother we'll say mightn't like animals at all. Which is..... you know they wouldn't give them the time of day like. We all had a dog you know when we were young....

PARTICIPANT 3:

…… I have a brother now, he'd pass a dog now but he'd have no love for animals at all. He wouldn't pet it now or anything like that..... & I have another brother that just loves them & his wife & his children.

PARTICIPANT 6:

Do you think the partner had anything to do with that? In that case his wife?

PARTICIPANT 3:

Growing up he never, in the family when we were growing up he would never have loved..... Brendan had, Brendan still has, the other brother still has..... once we had a little Jack Russell & he took the little Jack Russell off with friends, & he came back, the dog was being carried, the little Jack Russell was killed with him you know. I think it was that, that ever put him off having a dog & my other brother was it was his dog & he beat the daylights out of him really. Even his family doesn't like dogs at all. This other brother & his family does. The boys love dogs & when Candy, that dog that I now have, they were ringing me up. The nephews were ringing me up. It's only a dog but they loved it. They love animals compared to the other children you know. Of course that's the way of life in a family. Some like them & some don't.

PARTICIPANT 4:

My brother in law & his wife, they wouldn't..... well they do come in to the house but when we have the round for dinner or something else, but when they saw the dog we had, coz he's quite a big fella, I was introducing them to him. You know this is Max, he looked back & he wouldn't even..... he walked back out in the hallway. The children are kind of the same. They tolerate the animals, they don't..... I find that very strange. You know our family is... the dog, the cat anything is part & parcel of the family.

PARTICIPANT 3:

(*PARTICIPANT 3* SPEAKS BUT I HAVE NO IDEA WHAT SHE SAYS)

PARTICIPANT 4:

I think it's good for them. Because you can't escape cats & dogs in the street. You know you take them out & occasionally there's somebody.....they'll shy away.....

PARTICIPANT 5:

Like I said before we got our dog I wasn't that pushed about, now I grew up with dogs & there have been cats around. But since we have the dog I'm looking at all other dogs saying aahhhh look..... and I can't get over how I've changed, because they seem so intelligent now, they'd nearly talk to you.

PARTICIPANT 3 :

They're always there to greet you when you come back in.

PARTICIPANT 5:

It has changed my perception & out look on animals. To dogs.

MODULATOR:

Right.

Well what we might do is go on to the next one. So it's kind of like what your views are on neutering dogs & cats? Whether you're kind of pro or against or why you have if you do neuter your pets. Why you did neuter them. Or if you haven't why you feel like you didn't need to.

Just as well to bear in mind the idea of this is that we get everybody's ideas & everybody's views, & there's no such thing as wrong view. It's your view so we want to try & get everybody's view so....

Does anybody want to....? *Participant 5*?

PARTICIPANT 5:

Well when we got our dog, & she's a small Bichion, & the breeder only gave her to me on conditions. She said please don't breed her because she's too small.

MODULATOR:

OK.

PARTICIPANT 5:

And I was quite happy with that. So she was sorted. And I really wasn't prepared to go down the puppy route myself & put the boat out at that stage for me. Because I felt that the whole thing would curtail my life some what, having a pet because you know you go away on holidays, or if I'm away working, you have to make arrangements or you have to consider the dog because she is part of the family. So that's why I was quite happy to.

PARTICIPANT 4:

I think it's essential to be honest especially with cats, you know because you can't keep a cat in. I don't like cats that are kept in all the time you know & inevitably if you don't neuter a cat then you're going to have kittens somewhere. You know if you're lucky you'll be able to distribute them. Or you're off somewhere else & you get this population of feral cats which are problems for your own cats. We've had cats & you know you keep them in for as long as you can but you have to let them out & on two occasions, a cat we've has gone out & it has been bitten & it was a lot of work to get it back. It's been on a drip & God knows whatever treatment for it for ages.

With dogs, the first we had was a Collie & it was hyper. I used to take it out with me. When it was small, when I was working I used to tuck it into my overalls, & if I was working in a big area I could let it go & let it run around, but then it got bigger & I used to leave it outside, outside the house & it wasn't until a girl had knocked at the door & said you're going to have to put your dog in, no one can get down the street. Your dog just keeps them all off, & it really was. It was a friends dog that was enticing, she was in heat. In the end we had it neutered, tranquil*Participant 5*ers & female hormones & it was still chasing after it.

We lived next to the Garda Station in Irishtown at the time. I think the final straw was that it set off a Guard on a bike & the Guard said you're going to have to something with this. So, I know I think it's essential because, it stops them straying, it does quieten them down a bit & of course you don't want your dog, if its a male, if it's a female you know sort of Harry Potter all the time. If it's a male your going to run in to trouble at some stage, if it starts Rogering the door or it's some body else's.

I just think it's easier, & certainly my experience with the dog we have now tells me that some of the breeders, you know when I got the pup he said maybe I would use it in the future for breeding. I knew

that we wouldn't because at that stage we had got it neutered anyway. But given that the problems we had with him I can see why this fellow would be.... as far as I'm concerned there in breeding. It's in bred. It has problems, you know. So, that's another reason that I like to get them neutered.

PARTICIPANT 2:

We never had any problems really. Maybe it's because we had a smaller dog like. A Terrier or a Yorkshire or something like that.

PARTICIPANT 3:

I think when you get them neutered they are inclined to put on a lot of weight, & they lose their shape. Only when you get them neutered that's when.... & ah

PARTICIPANT 2:

Is it better for health wise to have him neutered or......

PARTICIPANT 3:

Is it better for them to get them neutered or.....

MODULATOR:

It's opinions really. It's part of the discussion. If anybody feels they can inter ject? I'm kind of sitting back on this one.

PARTICIPANT 3:

Ah I see yeah.

PARTICIPANT 2:

I think with smaller dogs in the house maybe, you know if you have a house dog. I suppose if you have a bigger dog that strays it may be a bit more, maybe, I'm not sure like maybe sure you can let them out for a while.....

PARTICIPANT 5:

The female is more at risk if you let her out.

PARTICIPANT 3:

Oh yeah, yeah. Well a small dog at any time really unless you're with them & you just keep them in when they're in heat. And I'll take them out walking. That you wouldn't have three or four dogs back at the house battling with you. Just keep them in really...... & I don't like cats.......

(HAVE NO IDEA WHAT SHE SAYS HERE)……

PARTICIPANT 3:

…… I've had none of my dogs neutered.

PARTICIPANT 2:

No we never gave it much thought really.

PARTICIPANT 5:

What's the span? Like how many years can they have puppies?

MODULATOR:

From once they start, until they die you know

PARTICIPANT 5:

They have no menopause then?

MODULATOR:

Generally speaking. No, no. it's not noticeable. I mean they're cycle might reduce just a little bit as they get older but in theory they could keep having them until the end. Yeah. Or it could be the end of them either.

Well *Participant 1* what do you.......?

PARTICIPANT 1:

Well when I got the puppy , I kind of wasn't that keen on getting her neutered & I probably had a vague idea that she was even though she was a nice puppy there'd be nice bred from her. She was mixed anyway. My decision anyway ended up having to be taken from me because, I remember I was walking on two occasions, being pursued by dogs & a taxi driver rescued me one time because he was experienced with dogs, & had a dog himself & this dog was jumping up. I was holding her up & he was jumping up on top of me as well. It was big dog & it was just up the road from where I live & the taxi driver brought me home. But the real decision was that she then got a false pregnancy & the vet said to me this can be a pre cursor of cancer type of thing & really I'd b better off doing it. So I did it etc.

So the advice was that medically I should do it, so I did it & I didn't really think about the rights & wrongs of it at all really. On the whole I suppose yes, it is more responsible & there are too many puppies around & too many dogs not getting done so in that sense, yes. I mean I'm sure I could have all sorts of ideas about the morality side of it, but they're not people, they're animals & I think on the whole I would say yes they should be.

PARTICIPANT 6:

……..As I said before, my mum….. we had puppies all our lives. The dog wasn't neutered. My mother wasn't really…. I mean my father wouldn't have been treating the animals as em…. they wouldn't have thought much about it. They just thought, oops goes again. At the same time they wee really…… they didn't want to put them down so we always had to look for homes & we always did, but the way I feel about neutering is I feel there are pros & cons & I don't think there's a right answer as you said. It depends on your case. You can't afford……. you can't afford to have puppies every time, but at the same time there are a very many options I think, & that's the way I see it. I think to if I were working in practise, I would like to talk to the person & say well, what's your lifestyle? What do you do? How many pets do you keep? Or, you know what I mean. I think there are a broad range of…. for example you have a male & a female. The male is vasectomised & the female is not neutered. Basically I don't mind them doing their business in the garden. And I think it's actually….. I prefer they did. I feel better if they behave a bit more like animals as they are & I think that's part of the being an animal. No thinking. They don't have any consequences. Now neither of the two would go out on the road so I don't have issues with the female getting pregnant or the male getting some other female pregnant because they are in the garden & they are taken out again so……

PARTICIPANT 3:

Yeah yeah....

PARTICIPANT 6:

I think there are pros & cons. Like the risk of cancer or whatever, so I have to balance & I still think that they can still have a much better life style, in just in my case, by just what I did, than with any of the other options, but that was only in my case.

MODULATOR:

I guess the other question is & a lot has already been answered. It's kind of what influenced you to have your pet neutered? I'm kind of looking for whether you have any media that had anything to do with it or your vet or anything like that or whether it was family history or kind of background interest as to why you decided to neuter or not to neuter or if you have anything to add to that kind of area, do you think?

PARTICIPANT 4:

I think that the first dog that we had, we were told that we wee going to have to get him neutered, he was so active. It calmed him down, put it that way, so it was the first dog I had, had other bitch's but that was the first dog. Since then we have had every dog neutered. There was one we didn't but generally speaking we would tend to neuter or spay.

PARTICIPANT 1:

I think I like the idea of them not being neutered, I think it's more kind of natural & respecting the animal & so on but I think I'd be very happy to be pragmatic about it, well I'd swing a bit each way on it. In my case it wasn't indecision, but with the second one we don't know if she was neutered or not. She's got medical things to do with the reproductive system. The vets don't know if she was neutered or not or if the reproductive system didn't develop or not so the decision doesn't arise. He didn't want to open

her p to inspect her because he said it would be very hard on her & might not necessarily find & all of this kind of thing, but she's had more problems recently & I think maybe he might be slightly thinking the other way. It's an on going thing.

PARTICIPANT 5:

And I suppose, like you said, you have a garden that the animals can.....

PARTICIPANT 6:

Exactly.

PARTICIPANT 5:

So it depends on your social circumstances.

PARTICIPANT 1:

You're able to go for a walk.....

PARTICIPANT 5:

In comfort.....

PARTICIPANT 6:

Our dogs they come inside....... Most of the time they are inside with us so they are..... obviously there is issues as well so if she's in heat I don't mind so.... I our case she's freaking, there are other people who wouldn't like the fact that she's in heat but I like the fact that she's in heat & that she's inside the house. I think it depends on what you really.

PARTICIPANT 1:

I bet your carpets love it....

PARTICIPANT 5:

Coz that was a bit of a shock to me. Before we had a dog I didn't know & after the first time I thought oooohhh OK....

PARTICIPANT 2:

I think the vet has a lot of influence as whether to, you know far more like you to have animal neutered. Coz I think when we go up to the vet he mentioned it so it seems to come up fairly regularly like you know. So I'd say the vets would..... it'd be business for the vets I suppose.

PARTICIPANT 6:

I personally wouldn't like that. I would be more like asking what's your lifestyle? I wouldn't like it having puppies that nobody wants & you have to put them down, but I mean if like you said, you keep your dogs inside....

(CAN NOT UNDERSTAND WHAT IS BEING SAID HERE)

PARTICIPANT 3:

….Yeah keep the dogs inside.....

PARTICIPANT 6:

……. it is something you have to do I think. There are pros & cons as I say.....

PARTICIPANT 3:

…..well it's only two to three weeks really, that you have to keep them in really. The only one’s that hyper really is Pierce. The Bird's dog Pierce?

PARTICIPANT 4:

No idea.

PARTICIPANT 3:

You know him?

PARTICIPANT 4:

I that a little black...?

PARTICIPANT 3:

Yeah, with a long tail.

PARTICIPANT 4:

Yeah I do remember, I do.

PARTICIPANT 3:

Yeah, that's Pierce & if he gets the scent at all he's away, & he's crying & winging & moaning. He's crying to get out & what not.

PARTICIPANT 2:

He's not able to run as fast as he used to so.....

PARTICIPANT 3:

No ,no. he's twelve or thirteen now so....

PARTICIPANT 1:

Thoughts on the question of you might like to have a puppy from the dog that you liked. The way mine is I can't. You know I can't continue with it as t were. So that aspect has to be taken in to account too, & whether it matters to you or not.

PARTICIPANT 4:

The dog we had that I said. We had the father & the father died. But I knew, you know, I knew he'd ad pups & I'd been talking to the owner & he said that when he was younger he said he'd vaulted three six or kennels in Sweden & he'd get through a six inch gap........ so maybe the walls in your place are.........

(NOT SURE WHAT'S SAID HERE)

PARTICIPANT 4:

……you know if you have a big male dog & they are active. You can't always take them out on the lead, you have to let them run. Like if they start running & they do get the scent they're gone.

TEA BREAK

MODULATOR:

……. I mean you can say if you think your dog is skinny or obese or anything like that or your cat. But it's more kind of what you yourself feel influences the weight of your pet? You know, diet & exercise & all those other factors that you think are involved so does any one want to jump in? *Participant 5*.

PARTICIPANT 5:

Well I have a non walking dog. She hates walking. She hides if we mention the word walk. She slips out of the room & I have to search for her, so if I'm busy she will not get a walk for a week you know

because she has to be encouraged out & stuff & once she knows she's on the homeward run she walks behind me on the way out & once we turn around to go home she's ahead & she has got heavy & I think, she's an epileptic as well. So there is a lot of tooing & froing with her medication with we've now got stable. So I'm not sure if that had anything... oh & she went on steroids at one stage so she ballooned.... &

NOT SURE ABOUT THIS BIT

(then she had bred? & she literally became like a brick?)

PARTICIPANT 5:

....so I've had a hard time getting her back on to the straight & narrow. So I feed her pet food, not tinned you know the nuts so I've had to get back down to one a day, but she'd nearly savage me for food during all that time, so she's a heavy dog but she's not fat as such. That's my story.....

PARTICIPANT 3:

I feed mine on chicken & Bakers nuts. Boiled chicken & mash the Bakers nuts. Break up the nuts & mash them all together. I heat the chicken. You see I boil legs of chicken & mash it all up together. She eats that. That's what I feed mine on. And I don't give her any sweet things because.....my husband might give her a bit now & then so other than that, that's what I feed mine on. I don't have anything to add other than that. I tried her on Ceaser & she didn't like it, she wasn't keen on it. Just to get her back on her appetite but......

PARTICIPANT 2:

I think everybody likes to keep the dog reasonably on the healthy side, you know you go out like & walk the dog & you might say are they looking after the dog, you know is he or she on the light side you know. I suppose you just.... when we have meals, we might give her something you know....

PARTICIPANT 3:

Tit bits.

PARTICIPANT 2:

Tit bits, you know. Especially when they're looking at out from under the table you know. It's just a thing you do I think you know.

PARTICIPANT 3:

She's in the house 24 hours a day you see. I don't like having a dog & having her outside all the time & not coming in.

PARTICIPANT 2:

You find some dogs, you know Terrier wise that some of them are easier to train than others. Even let them be the same breed, but some of them don't catch on as quick. You know some dogs would scratch the door to get out & you then others wouldn't like you know for some reason I don't know why. I suppose they're all different in their own way too.

MODULATOR:

That's true.

PARTICIPANT 3:

Mine goes all night sleeping on my bed, she goes all night & first thing in the morning, she runs down & out on the grass. The only place she'll go is on the grass she won't go anywhere else only in the grass.

PARTICIPANT 2:

She really likes to walk in the grass.

PARTICIPANT 3:

When we're out walking now & there's grass she'll go to the toilet.

PARTICIPANT 5:

And would she think the carpet was grass?

(ALL TALKING OVER EACH OTHER HERE)

PARTICIPANT 3:

…..she's not allowed to wander around the house on her own. She's not allowed up stairs.

PARTICIPANT 2:

She doesn't drink that much water any way. A couple of scoops & that's it like you know. We had another Yorkshire & she had diabetes & you know we didn't realise that drinking so much water you know & that's why like because she had diabetes & she had to be injected all the time & you know we had her at home you know. So I suppose if you see a dog drinking a lot of water, not necessarily, but it could be a sign that, on coming diabetes like you know. Maybe giving them too many sweet things & you know it brings it out in them you know. That's what we learned any way from that bit of it.

MODULATOR:

So *Participant 1*, what about yourself?

PARTICIPANT 1:

Well I feed them on, you know the sort of dried food, & the decision I made right from the start was that I didn't want to be worrying that I was feeding them properly or not & I just decided to go for the dried food & I always fed on the basis of just leaving food out. I didn't measure it or I didn't anything & she never really put on weight. I wouldn't say she was ever really skinny. She wasn't like skinny, skinny skinny or anything. She never really put on weight until she was about fifteen I think & then she put on about a kilo, but she'd been the same weight for years I think, in so far as I weighed her, but then she got an awful lot of walking. I'd say more than a lot of dogs get, which I think probably helped & lately I've had, this is the older dog I'm talking about, she's on special food because she had a crisis. You were the dog vet when we brought her into the emergency & she was on drips & everything for days afterwards. She's on this low protein food, I'm not sure if she really likes it. You have to add bits into it, cheat a bit.

But basically my view would be that the commercial food is the simplest thing, coz you could end up, well I felt that I would end up being a prisoner of the dog & cooking for the dog & everything. I didn't ever feed her from the table or anything like that. I never found the food end of it a problem & that the Jack Russell one would tend to gorge if she if she got a chance & I feed her a mixture, I got the weight food for the other dog for a while. What I was feeding her t& his weight maintenance. I just had to harden my heart a bit & I just don't give it to her. Her weight varies a bit but she does get quite a bit of walking.

So the question there, what factors to we feel effects the weight of your pet? I mean I'm not an expert. I just didn't have that problem with weight, but they do get a hell of a lot of walking & I suspect that makes a difference. I always wanted them to have at least the minimum of an hour a day, but they always end up getting more because I'd always have a least two walks in the day. Even the one now, she's sixteen, she's a month to go. We would get out for that length of time. We're near the park & it's very handy. I think all of that effects their weight I suspect, but that's just my experience I don't actually know.

I could be unlucky I suppose & have another dog that would be just impossible but would just like put on weight like people do.

PARTICIPANT 5:

Certain dogs do tend to put on weight do they? The Labrador?

PARTICIPANT 4:

The Labrador yeah.

PARTICIPANT 5:

Is that just a fallacy or....?

MODULATOR:

I t really depends on their activities.

PARTICIPANT 1:

I would notice the amount that they eat, now I never kept a study on this, but I always noticed that the amount that they ate seemed to relate to the amount they were walking because if ever I had a day that we didn't walk as much or the weather was a bit different, I would just notice they didn't eat as much, so it seemed to me to be self regulating almost. It was my impression.

PARTICIPANT 6:

I have a dog that will prove you wrong anyway.

PARTICIPANT 1:

Really?

PARTICIPANT 6:

You can feed her anything & she will keep eating, she loves..... she's a German Shepard, she loves eating & she gets quite a lot of exercise. We take them in the evening. We have a couple of horses as well so we take them with us & let them run for an hour or two hours. So they are running. Quite a lot of exercise. They love hunting rabbits, they never get any, but they love chasing rabbits anyway & things like that so they do get a lot of exercise. But she could eat for Ireland basically, & she eats everything....

PARTICIPANT 1:

And does she put on weight?

PARTICIPANT 6:

Well we are very careful. This is the dog we got from the clinic & we had surgery on her. She is 90% better but not fully. She's not perfect, we never got it perfect. So we have to be careful to keep her light. We cant afford her to put on weight basically. So we are very careful with her so I'd say she's a little bit than she should be. But I prefer her....

PARTICIPANT 1:

It's probably better for her.

PARTICIPANT 6:

She's not lame at all, but when she puts on weight you can start to see a bit of lameness, but there's not much we can do at this stage though. So we prefer to keep her from limping. When we send her to my partners parents for them to mind her for a while, when we go on holidays or whatever, she comes back fat.

MODULATOR:

Doting grandparents...

PARTICIPANT 6:

….there's stories in the family where one Christmas she went to Galway to my partners family & they were cooking a leg of lamb. Obviously she went in to the kitchen, got the leg of lamb, once it was cooked! And headed outside so she could eat it....

(LAUGING HERE)

PARTICIPANT 1:

……. for the sake of the weight type food. The Jack Russell now, she's not bad at eating it but she'll eat anything else before the weight food, but she will eat the weight control food so what I do is, I'm a bit calculating about it, I just make sure that there is some of it there & if she really is hungry she'll eat & if she isn't she'll just leave it. Then at least I say that she has had enough. So that's what I do. It's a bit cruel in a way because she wants the other dogs food, but I have to put her in a separate room. The other dogs food is more tasty. You just have to be a bit ruthless I feel. You feel you want to do what they want…….

HAVE NO IDEA WHAT IS BEING SAID FOR THE NEXT COUPLE OF LINES

PARTICIPANT 4:

I've got one plumpish dog, the small one& she's on a low fat diet, which is supplemented with a little bit of cat food or chicken. Everything is chicken, even the act loves chicken. The other dog has renal problems so he's on a renal diet so he can't have much more than that, but before that the Labrador would eat anything & he would put on weight no matter how much we exercised him, & we'd try & exercise him properly. The dog that, the Pointer that we had before this one, like your dog, we bring him out & he'd just run & run & run. Your dog runs with the horse & I always thought would run about twenty miles. Take him out & he'd go chasing after birds & he'd go all over the place & you'd lose him for an hour so you know he was really skinny when we got him, we built him up, but I never felt there was any problem as long as you know if you do give them plenty of exercise. The smaller dog just follows behind me, you know if I do three miles the dog just follows behind me. I think if I ever gave him the option he'd go back home....

CAN'T UNDERSTAND WHAT IS SAID HERE

PARTICIPANT 4:

….. my wife & I like walking & we go on walking holidays. So we go out & we walk & the dogs either chase up ahead or they follow behind. They're getting that bit of exercise anyway. But usually it's dry food supplemented with... usually a bit of cat food. We don't buy much dog food any more because we always have cat food & then it would be chicken, a little bit of liver from time to time & sardines, mashed in together & a bit of oil for their coat you know.

MODULATOR:

That was one of the other points I was just kind of wanted to discuss. Kind of home made or commercial diets. What your views are on that?Whether you think....you know why you pick either & some of you have just kind of commercial diets, some people kind of mix it up a little. Do any of you use just home made diets? Without using any commercial food?

PARTICIPANT 2:

Well we really just feed... we boil chicken you know which is what we eat anyway so there wouldn't be tins or anything like that. We buy the nuts as well like. You know the Bakers or.....

PARTICIPANT 3:

We buy the legs of chicken, & boil the legs. Nearly two a day & mix it in with the nuts then.

PARTICIPANT 2:

The dogs just don't like the commercial stuff like you know all that stuff you get in tins. She won't eat it sure she won't.

PARTICIPANT 3:

No. When she was up with the vet now she wouldn't.

PARTICIPANT 2:

And she makes her own decision on that.

PARTICIPANT 3:

Richard was trying to feed her with it but she wouldn't touch it.

PARTICIPANT 5:

The lads now like tuna in to the nuts. Now initially because she could go two days, three days & not touch it, & then once I had put the bit of tuna in she'd eat it no problem, but now she just, that phase when she was really hungry she could eat the leg of the table! I don't have to put anything on the food now she just goes straight for the nuts & sometimes I put frozen peas or something just to bulk it up for her. Like I said she's back on the food, & that seems to do that & she seems to enjoy raw carrot & red peppers. You know the raw vegetables. I give her the raw vegetables as a treat. She has been known to demolish a half an Easter egg. That's how we ended up in here! So anything chocolate can not be left at her level or you know within reach because she will just go in & seek that out. You know toss the lunch aside & nothing can be left on the floor like that.

PARTICIPANT 1:

The reason we went for dry food as well was, it just reminded there what you were saying, that you don't have this question of if it's a tin you know how long do you keep it open & in the fridge & all the rest of it. I just felt from that point of view it was easier, but recently because they are both on loads of pills, I started buying those little small things, the little Ceaser things they're called to help them eat but the trouble is the Jack Russell she can somehow manage to eat it. You could be there supervising her & the pill will drop out of her mouth while she's eating a bit, & the solution..... I also use some of the Ceaser stuff to go with the renal food to try & sort of spice up.... I also have, I try to break up those Teedy biscuits, there some sort of..... they're food for..... I think they're for..... food for teeth or something, & I crush them up & put them in, this is for the dog who is supposed to be a low protein diet. But, the solution I found for the pills is pate. Tiny little bits of pate because it holds them very well & in fact when they take the pill in to their mouth quite often a bit of pate is left behind. I'm sure the pate is bad for them! But they are old & if they don't get the pill, well they're not going to survive & I just find that now works & they both take the pills & there's no problem. And I have to keep buying, I feel like a fool in the shop, I told them like 30 cents of pate. You know it's very difficult for them to serve out that much. It keeps going past the sell by date. I think with dogs you can go a day or so past the sell by date but at the same time you just don't know.

PARTICIPANT 5:

I have one capsule & one ordinary tablet & I put them both in to her food you know. Camouflage it.

PARTICIPANT 1:

I wouldn't risk that...

PARTICIPANT 5:

But she will always take the tablet & leave the capsule behind. Always. An odd time if it's buried she eats it unknowingly but it's the one that she needs so I have to put it in a bit of cheese or a tiny bit of butter or something & it's down before she knows it.

PARTICIPANT 1:

What I do is, you know the card board things the packets of rice, that kind of thing. I cut a piece, a small piece & I fold it over....... & I put a little bit of pate on to the pill on it so they get taste that's going & they can see you doing it & they are there looking & they just grab it & then you just dump the piece of paper.......

(LAUGHING)

PARTICIPANT 1:

…….you don't have to be supervising them then after that. The Jack Russell I make sure she's out on the floor so that so that if it did fall out of her mouth then I'd see it. Coz you don't want to find the pill in the bed ages later & then think which one is hers.

PARTICIPANT 5:

Or it gets stuck in the fur. Sometimes I mite find it...........

PARTICIPANT 1:

You really have to be watching.

PARTICIPANT 6:

We use a commercial food, a dry food as well but they recycle any food that is left basically. So if there are any left overs, I just give it to them & they eat everything. They eat potatoes, tomatoes, salad. They love a salad & I just pour the oil over it. I put the lettuce & tomato in on top of their food. Anything.

PARTICIPANT 1:

you just ad this to the dry food do you?

PARTICIPANT 6:

Yeah. They get their dry food. That's everyday but then if after I have my dinner we have dinner & there's a bit of salad, because they eat after us. I will give the left over. There isn't much in fairness. It's not that they get a meal out of it. They just.....

PARTICIPANT 1:

They just get flavour.

PARTICIPANT 6:

And then depending on how much I have left over then I might reduce their food....

PARTICIPANT 2:

Well it's expensive feeding you know like processed food all the time. Especially if it's a big dog.

PARTICIPANT 6:

You meaning processed, you meaning commercial?

PARTICIPANT 2:

Yeah, yeah. Commercial.

PARTICIPANT 6:

Honestly. To be honest sometimes when I go to the super market & I see the prices of the dry food, I think about cooking myself but I don't think I could make it much cheaper. Could you?

PARTICIPANT 2:

No, no.

MATLIDA:

I don't really know. Maybe I should try. That for me, dry or commercial food is handy. I don't have to cook & in theory it's balanced. In theory but in the end you don't really know either........

NOT SURE WHAT IS SAID FOR COUPLE OF LINES

PARTICIPANT 1:

I suppose when you see your dog is healthy or not that's how you judge it.

PARTICIPANT 5:

And I think the poo's are smaller with the dried food. Less messy.

PARTICIPANT 6:

They are always big when you have a German Shepard!

MODULATOR:

Go walking with a shovel.

PARTICIPANT 5:

You see I'm the shoveller around the garden!

PARTICIPANT 6:

I took them out. I like telling the story.

MODULATOR:

Yeah, that's all right.

PARTICIPANT 6:

Sometimes I take them out at the green area behind our house. So Lumas has just went for a poo & I said I'd pick it up & rather than carry the bag all the way back to the house, I was passing by the back of my garden, so I decided to throw it in, but somebody caught me doing it & they were looking at me like, What!!!!.........

NO IDEA WHAT IS SAID HERE.

MODULATOR:

OK. Well we'll move on to the next one. It's kind of. The next, last two questions kind of filling together. So it kind of looking at how important, so both to you, yourself as a person as a pet owner, & to your pet that you feel that your exercise routine is, & how important that time is that you spend with them. Do you think they benefit & do you think you benefit from it? Things like that.

PARTICIPANT 4:

I love getting out. I love walking. Now having said that, the dog that, the one that, he's got something called LUG BOOM?...he just can't walk. He's always limping all the time. So he hadn't been exercised for a couple of weeks. But before that I mean.... I do , I like to get out with the dogs. I like walking myself & I just love to see them. I love to see them getting out & running around, you know. One of them, the small one, doesn't do much, he just follows me, but the other one, the Pointer, he one we had before, they just..... it's just wonderful to see them. So I love that side of it. We keep them in doors & yeah we spend time, not just exercising but actually just having the dogs there, I feel happy with them.

PARTICIPANT 3:

I think it's just great having a dog if you have children. It makes children more responsible if you have a dog.

PARTICIPANT 4:

I think so yeah.

PARTICIPANT 3:

Because I have a little grand nephew now & he's just mad about her when he comes home form England & you know, he just loves the dog.

PARTICIPANT 2:

DON'T KNOW WHAT HE MUMBLES HERE

……….

PARTICIPANT 3:

…… to make them responsible. Children, you know.

PARTICIPANT 5:

It's such a pleasure, the welcome you get from them when you go out. *Location 5* beach or strand is her favourite. She will go any where in the car. She will go for a drive. She will nearly head butt the door to get in. When I say will we go in the car the head goes. We can go to *Location 5* beach & she does like that. So it's lovely to walk up & see other dogs & owners & not on the lead.

PARTICIPANT 4:

There's a shelter side as well......

PARTICIPANT 3:

And you get to know people as well.....

PARTICIPANT 2:

Did you train your own dog like, the basics?

PARTICIPANT 4:

Well not.... I could.... well they are good on the lead I would say.

PARTICIPANT 2:

Do they pull.....?

PARTICIPANT 4:

Nothing too much. I don't think I could afford to take them somewhere to be trained to be honest. So, I mean all the dogs we've had they are reasonably trained. Not perfect. They'll come back you know when they are called & that's, as far as I'm concerned, that's good with dogs.

They love to run if they wanted to come back, they'll come back, & to me that's a trained dog. It doesn't perform...... you know I'm not looking for it to trot behind me & do this & do that you know. I'm happy enough to walk the dog safely. Know that he's safe or she's safe. They they don't go after somebody else & doesn't bolt out in to the middle of the road, that sort of thing & comes back when called. That's trained to me.

PARTICIPANT 3:

Oh yes, they have to obey, yes.

PARTICIPANT 6:

I love taking them out, but sometimes they are a bit of a pain. For example, just going back again about neutering or not. Obviously if she's in heat or whatever they male might be a bit more aggressive, so I have to keep him on the lead all the time & I don't let him out. So I suppose again one of the cons of not

having them …… so I suffer that, but I'm fine. But I love taking them & when we go to the horses, because there are no dogs there & they won't disappear. They just chase us around & they come to the car. They are just too afraid of being left behind. So they come back to the car.

I think they enjoy it. Obviously they enjoy it. I do enjoy it as well having them there.

MODULATOR:

Participant 1?

PARTICIPANT 1:

Well walking is very important. I think from the dogs point of view, I really feel they need to be exercised & they love it. Well with both dogs they are enthusiastic walkers. It doesn't matter what the weather, they want to come out walking. Which is great & one of the things I noticed when I had the first one, is I didn't have experience of walking dogs so I noticed I'd be walking along the road & there would be a stern, stern person coming towards me & they would relax. They would look at the dog & you'd see them relaxing & even smiling. They might not look at me necessarily, but other times then they would smile at me as well. The dog seemed to have an effect on other people I noticed.

I also noticed that I see things differently with the dogs, & I'm particularly conscious of this now because the older one is so slow now that at this stage when we go in to the park we stay near the gate because I'm afraid she might have a heart attack & die, she might collapse & not be able to come back, & literally she weighs about twelve kilos & it would be an awful nuisance. But I just feel it's good to get to the park, because on the grass she's sniffing the whole time & I really noticed, well I noticed it before but I really notice it even more now because I'm not getting the exercise with them. The way they are sniffing they are totally concentrated on the sniffing & they are totally, they are what they are, they're doing what they're doing. There's no past, there's no future, there's no worries, there's no thinking about it, they're just doing it. And you have to then just get in to their routine & whatever & I just see the park looks different when I'm with the dogs as well. I just find it fantastic, & then of course you meet all sorts of people & you get to know them at a particular level of meeting people in the park & talking about dogs or whatever you talk about, & don't usually get to know the people much better. Other people you sometimes do, but the people are very friendly or the people are generally very nice & it's all a very nice thing really.

And then of course walking on the roads when I used to do that, you see people or you'll notice the extension on the house or whatever & now when I occasionally go for a walk with the Jack Russell on

her own or I might be walking somewhere on my own, along a road I'm used to walking on, I can say “oh God I didn't notice that because it's so long since I've been there. I think it's a whole, you see everything differently with the dogs I think. I think it's great. And I love the way, you know they never seem to get tired, and they seem to relax more when they've exercised, but if I get up to go to the shop, they're up to out again. I'm just really impressed with them. It's wonderful.

MODULATOR:

That's true.

PARTICIPANT 2:

There was programme there just on the telly. It was just a thing about, well it asked if dogs were colour blind, and are they colour blind? Or is it a do we know, or do we not know?

MODULATOR:

I suppose they see things different to what we see, to a certain extent. Their eye position is a little bit different.

PARTICIPANT 1:

If you get down low towards them they seem to see you better, from what I notice.

MODULATOR:

They've a different positioning, so they don't see the same 3D that we do. Yeah, yeah. Their periphery is better than ours, because of where there are situated in their head, more so than anything else.

PARTICIPANT 5:

Well I'm, we over look Dublin, you know just up in Mount Merrion & the dog goes out every evening & she just sits on the step, she settles down. You'd think she was looking at the city lights. So I've gone out & just sat down & I said “what is she looking at?” sure she can't see beyond! I really don't know what, it's very calming & she's looking there & she'll scratch to get out, & she'd stay there for an hour just.... especially with the twinkling lights. She can't see!

PARTICIPANT 6:

I have come to the conclusion that my dogs can't see very well, but they can count. We have two cats right, so if they see one of the neighbours cats, which is very similar to our cat, they don't like cats but they live with two cats & they are part of the family now so they completely accept them & they are fine, but they don't like the neighbouring cats. If they see one that is similar to our cat, they can't differentiate if it is ours or not, but then if they see there are two cats! Then they know. So they obviously can count! It's mad isn't it.

PARTICIPANT 4:

I had two cats until recently & we lost a cat. It just saw it one day at the top of the stairs. So I went out in the garden maybe twenty minutes later & dead. It was ginger Tom & the cat that we'd adopted was a similar ginger Tom you know, but it would never come in to the house, where as the other one was always around the place. Obviously they could tell that the one that never came in was the one that could b tolerated until the second dog. Now I don't know if it's a Pointer thing, birds, straight for them, cats, doesn't seem to mind them at all. So the feral cat, if the smaller Spaniel is in the living room say & the bigger dog is in the kitchen, that the cat will now come in & the big dog has no problem at all, but if he hears the Spaniel, he's out like a shot. I don't know if the Spaniel knows that the other cat has died or what. He will not tolerate this other cat. It's a peculiar thing.

When we had the first cat it was part of the family. I would just you know wander around purring. He was no problem. The two were very similar.

MODULATOR:

They know well enough all right don't they?

PARTICIPANT 2:

Is it good to have two dogs? Is it company for a dog?

PARTICIPANT 4:

Well I think so. We always kept an older one & a younger one. That's the pattern we did adopt. You know we had an elderly dog died, & his replacement wasn't the youngest dog. It was the first Pointer that we got, as a rescue dog, but he only lived for about four months. You know he got that spinal legion. I think he always had the spinal legion. He all manner of problems to be honest. And then comes on his puppy & he's about nine or ten months now & how long he has to live I don't know. Matter of months I would imagine. But I think it's good, even a big dog or a small little one. To my mind a small dog, they don't particularly.....some may a little bit different. Maybe there's a little bit of the hunter in them., but some of the smaller dogs they don't always relish exercise. But the bigger dog.......

NOT SURE WHAT IS BEING SAID HERE

PARTICIPANT 3:

…….there's some days they may not feel like an old walk.

PARTICIPANT 4:

The thing is with mine they are always playing. The small ones the grumpy dog to be honest. The bigger one will........

the two of them will play & we tossed it over the sand. You know rolling over. It gives them some exercise.

MODULATOR:

Very good.

Has anyone anything else they want to add? or are they happy enough with all that they've said & done at this stage?

That's great.

Well thank you very, very much for coming along. It's been brilliant.

THE END.

Location 6

Modulator

What we're interested in finding out is, why you have a pet, in the first place and so if any body wants to volunteer?

Participant 7

I was brought up with cats all me life, and there was cats in the house from the farm, therefore I like animals and thats more or less why we have a cat, they were part of the Family

Participant 1

Same here, when I was growing up we always had cats, even when my father put all the kitens out in the farm in the country. They're great companions. I think it is very sad when somebody doesn't have a pet, when their children are growing up without pets

Participant 2

I was exactly the same, I always thought it slightly odd when people don't have pets, but its always the personal choice em in my case it was always cats and we always had them one after the other. and they all died naturally in then we used to get adopted ones, we became a cat house in the neighbourhood, cats came through and there'd be a stray and it would adopt us and thats how we'd end up with the next cat. to me when I first moved out of home, I could wait to get some where of my own so I could get a cat of my own.

Participant 8

I don't think the house is a home till you have a pet, it just completes the family to have pets and I think it is very important for children to grow up around pets too, because if they don't they tend to turn into people who are not nesecerily kind to animals. and if they are not kind to animals I suspect they're not kind to children either, I think it is all bound up together, I think it is very important to have children grow up interacting with animals and understand how to look after them properly, and treat them properly and they are much more likely to turn into responsible adults who will also be good with children and good at raising families. And they are great company too, couldn't live with out them.

Participant 3

I grew up with animals as well, and I suppose thats why I went onto have them myself, I've never actually been without an animal as Participant 2

ie said it's very important I think in childrens developement to grow up in a house with animals because they learn the responsibility of looking after the animals even though the mother of the house is looking after them but they get to see what is involved, there is the walking, there is even the expense of the vet, you know they get to see the whole wider picture so there not likely when they leave home to go into a job to go into an appartment to get a dog we'll say for christmas and then discover they can't look after it. You know, they know the nuts and bolts of the whole thing, My own children have left home now, would love animals and but like that they they'll just have to wait till they're around more actually my son has a bearded dragon, but I mean you can go off to work and leave him be. but they know the responsibility involved and I think that is hugely important. Animals do make a home deffinitely.

Participant 4

Dogs, I've always loved dogs, always, loved dogs. em my son loves them but doesn't always look after them though... We have 2 dogs, and he actually has a snake, I won't go near the snake... But dogs yeah I do love dogs. there kind animals and they do protect you, some of them. The great dane is a chicken..

Participant 9

em the same as everyone here, I had/ grew up with always a dog in the family but 2 years ago em the one dog that I would have classed as my own first dog that I was old enough to really form a bond with am past away like and since then the 2 dogs that I have now I would class as mine I picked those dogs, I got them because I wanted them and they're not just wht my family had, for me to enjoy as well so, I think now after expieriencing the love of a dog or of a pet really entused me and made me realise how much I really love having a pet there or a dog there, that was my reason.

Participant 5

I would have a pet and this is going to sound really bad but ah we just got our dog at christmas time and we got him as a gift, I was brought up with a dog and have always wanted to have a dog, there has always been the discussion you know, about working and leaving the dog on its own and that, so at the moment the reason why I have a dog is because it was given to me as a gift but it is the best gift I've ever had, am but it is a lot to do with growing up because we had a dog then, you know and ass they say there part of a family and it is nice to have that companionship with them as well

Participant 6

I'll have fallen into the same category, there was always a dog at home, growing up, so when I got married and had children it seemed the prety obvious thing to do, get a dog, and we've always had a dog. we'd have had one or 2 that have died of natural causes and old age but sometimes we've said wel l we won't get another thing, but someone will come along and offer you anew one or whatever and you go down the road and you start again, so em, yeah we've always had a dog so theyre part of the family you know. Now not all of the kids take to dogs, or rather not take to any given dog that we've had some have liked and dissliked we've never had everybody like the same dog thats the thing with children, so I don't think that you can take it that children are , or all the children are just going to like a given dog but em yeah thats where we would come from

Modulator

I'm going to stick with you seen as we finished with you.. why did you choose that type of pet, so with your self why did you choose a dog?

Participant 6

em, well I don't like cats, sorry for that, (laughing etc, some aggree some dont)

Participant 7

It's not that people don't like cats it's that theyre afraid of them, thats what it is, I had a neighbour that said I don't like cats keep them away from me, but it wasn't that she doesn't like them it that she was afraid of cats, because when she was a child someone threw a cat at her.

Participant 4

But I can't stand cats, I cant have them around I'm just terrified of them, cause something like that happened to me about 12 year ago, where 2 of them tore my face, well I'm just afraid of them, I like them though but I can't be near or around them, my mother has one and I won't go into the house, when they're around unless she puts them off somewhere,

Participant 6

We've had other bits, fish you know the usual, gold fish that don't last, canaries that last a few years all this type of thing, but we've always had a dog, dogs are part of the house you know

Participant 1

yeah just always had cats, but I don't know I think they just came in I don't think my mother or father really wanted them there but I liked them, and ah always had a cat so never had a dog, I think I'd love a dog but I can't get one know with the cats so I think theyre too old so am I understand that cats are very independant and they'll only do what they want to do so I suppose it takes am, well we can train them but you can't order them arround they'll just do theyre own thing, but I like that, I don't mind, of course they'll go for you, they'll jump up or, at the end of the day.

Participant 4

These were stray that I was attacked by

Participant 1

They can be very frightening

Participant 4

That time I think the mother had been killed and I just went out with the food, it was dark and they were frightened

Participant 1¶

yeah yeah they were wild animals like yeah

Participant 4

That was the end of me and the cats

Participant 2

for us it was the cats, for years we had 2 siameese and a burmeese for many years and they'd talk to you they are so intelligent, there wounderfull creatures, and very affectionate, what I love about cats is that they can be free, I hate to see things caged I can't bear to see things caged, I could never have a pet that would need to be in a cage, I just don't like thatI feel everything has to be free, so I could only have a pet that I knew could be free, so the cat is ideal for me because it can have it's cat flap, luckily I live in an area where it is safe to let the cat in the garden, they have plenty of space to run, so now I have a rescued tabby when the 2 old cats passed away i said I wanted to get a dog for the first time, I hadn't got one before cause I feel it wasn't fair on the older cats in the house as they would have been too tormented and I didn't think the circumstances were right cause I wouldn't have an animal if I had to leave it all day closed up or I felt I couldn't provide for it sufficiently but I wanted to have a dog because I wanted the expierience of having a dog and I wanted a little security and I wanted something that would make me walk every day I'm near Killiney beach and I should be on it every day but without the dog occasionally I might go, but with the dog I'd never question it. It's 14 months old and we go to the beach 5 days out of 7, she's a water baby she's wounderful now, it was a very steep learning curve

for a first time puppy mum it was hard work brining up a cocker spanial on my own but not knowing what I was doing but she's turned into a lovely dog and she's great protection and great company and she gets me out walking so she has the lower floor of the house to herself, and the tabby has the upstairs part in peace, she wants to be freinds, but she is too big a boystrous for him now, so I think it'll be a while before she calms down enough for him, could take years for him to be friends again. Theyre such good company, I did swear for a little while that I would have no more pets when the last generation of cats died, because I'm now on my own now that my parents are gone, and I thought well I want to be free to do travel and all of that, and I won't have the responsibility of pets and after my fathers burmeese passed away at 191/2 years it took 10 days in the house with it empty when I was on the internet looking for a rescue cat I had to have another cat I couldn't deal with it. I'm back to getting a housesitter when I go away because I can't bear to kenel them, I wouldn't do it, I'm real soft for them.

Participant 8

I've always had cats, I'm perfectly happy been a cat person, I would firmly put myself in the box as a cat person, my lifestyle wouldn't suit a dog at the mooment and you identified that a dog is a new learning curve, theyre a different type of animal and thats another reason I don't want to go that route, so I'm perfectly happy being a cat person, but I've always had cats because they were always the animals I had when we were young and also when I was a kid i seemed to be fasenated by all things cat and I had a picture of a tom cat on my wall and i had other pictures on the counter and I had the aristo-cat off by heart when I was a kid, cats and me always seemed to be. There is something about cats thats facinating, now alot of the cats I've had, they seem to have an undeserved repitation for intelligence, because my present moggie is a bimbo I had cats when I was young and they were very smart, they could open doors and that kind of thing, but em ,they are a very interesting animal and you do get the sence that your priParticipant 8

idged to live with what is still, well you can see the wild instinct in them, there just very interesting animals, they do tend to bend around you which is rather nice and em I suppose it's a bit like living with a very young child or a very old person, you have to get them into their routine and they get very insulted if you take them out of it and that kind of suit if your that kind of person, and thats the kind I am, so it's never been a choice for me its always just been cats-

Participant 2

I think cats keep pet humans, humans keep pet dogs but cats keep pet humans

Participant 8

Dorothy Perkins "your nothing untill you get ignored by your cat"

Participant 9

I'm a very dog person, I put myself into that box...I've never had a cat, I've no desire to ever have a cat, em dog for me, em having a dog is who I am I can't imagine myself without one. and thats really why I would choose to have a dog. I just couldn't imagine myself without one, to be honest so I'm completely oppisite, and I don't like, I like the fact that a dog is very dependant on me and very dependant for attention, and for love and for affection, for everything that goes with it, I'd be offended if my pet ignored me if I came in and they just decided that the weren't bothered to come home, I would be very offended thats why I like to have something that really wants/needs my love and I just enjoy giving it to them, knowing that the need it so, thats my choice.

Participant 5

I'd be of totally of the same opinion, I like the way the dog totally depends on my and I'd always be a dog person, and I think the reason why I choose it is because it is s odependant, you can set a routine and you can also teach it things sometimes it will obey you an dsometime it won't but thats OK. I just like that and I just like that you can look after the pet, and he always seems to give some sort of love back, I just see him as my dog and he always seem to just respond and that would be the reason why I go for a dog

Participant 4

I love dogs, I remember having a dog when I was younParticipant 1¶

, I was about 7, and he actually saved my life, because the ball went out on the road and we were living on the main street and ran to get the ball and there was a truck coming down and he pulled me back in but what ever way he pulled, he went out and he didn't make it. I always loved them and my son always wanted a dogand we were living in a msonette so we couldn't have one, sas we had no garden so he got a rabbit, so the rabbbit became the dog even had a lead he'd walk up the road with the rabbit and around the area, so I thought when we get the house we'll allow him get the dog then. so that happened when we got the house with the garden and then the girlfriend came alongwith a bigParticipant 1¶

dog so know we have 2 dogs and a snake, but I won't go near the snake, thats out of bounds,

Modulator

Thats in the same categoty as cats...?

Participant 4

cats is only cause I'm afraid of them with the snake there's no way I'd even go near his room, but dogs, no I worship dogs, I love dogs, It's just as well because I'm left looking after the 2 of them.

Participant 3

I have 2 cats and a dog and up until recently I had 2 dogs as well ah and we bred budgies for years as well, but ayhow we're down to 2 cats and a dog and am I'm both a cat and a dog person, but I do like the way the dogs depend on you the way he expects the walk and you know there are certain things the dog expects, and is bulit into your daily routine, and I also found that, well it's always terriers I've had, and their great fun they really are game for a laugh all the time you know, so they really do put you in good humour and I just find that is fantastic , I suppose I'm really founf of the cats as well the dogs are the fun in my life really.

Participant 7

I have 3 cats and 7 dogs thats what we got they weren't ment to happen. My son was going down to wexford one morning at 7 o'clock on the motorway, he was going one way there was a van in the fast lane going in the oppisite dirrection. the threw the dog from that lane into this one the dog is known as dodParticipant 1¶

he dodged the traffic then we have another 2 when me mam and dad died near 11 year we got there dogs. and a friend of me husbands he had a little dog and he wasn't able to take care of him so we got that dog as well. then joseph the son he was on the motorway passed loughlinstown and he came accross 2 dogs on the motorway a shtzu and a little terrier a little black one, tom and jerry. he took them to the barracks but they had no place for them he said he'd get the animal rescrew croud out but he didn't in the end. then 3 days later he took tom to the vet becaouse his coat was very damp the whole time, i knew there was something wrong with him and he died in me arms. Teddy then he escaped out of the pound in rathfarnham and, I took him for myself, he's my dog. Linda would take him up to the horses and while she was feeding them teddy was off the lead, and someone stuck a steak through his side. but thats all the dogs.

Modulator

you need to tell your son to stay off the motor way

Participant 7

He's an unusaully one, his mammy is a black lab and his daddy is s scot' terrier. Lizas husband had them and I took one for me son because I said what will he turn out like. He's georgeous, he has a big head on him, some people say to me is he a big scottie?

Participant 3

My sister has a mongrel sheep dog he has a big body with tiny legs, he looks like he has been stuck on to small legs

Participant 7

I love the cats, I like the dogs but the cats are my favourite, and I love teddy (my dog) teddy is my baby, but he has diabetes and even when the kids were brought up if they got an animal they were allowed as long as they paid there veterinary expense. I didn't mind feeding them but as one of my sons said I used to go some place to cut grass and by the time I had it saved it had to go to the vet again.

Modulator

We'll move onto the next topic, interested in what your views are on neutering dogs and cats....

Participant 7

Well all my cats are neutered and the females in the dogs are, only one male is neutered

Participant 1¶

And why would you neuter the females and not the males

Participant 7

Because we have mixtures and we don't want pups and we have them in the same garden and house and that and we didn't want pups thats why, and its saver with cats cats get diseases and stuff when they're out and around whereas the dogs are more home birds

Participant 9

would it have not have been less expensive to get the dogs done than the females?

MarParticipant 1¶

et You see when your out with the female trouble could happen then at least with the male your kind off saver where they are

Participant 1¶

there easier to manage I suppose

Participant 2

I would always neuter all my pets both male and female, I just think there are so many onwanted living creatures in the world that it is just I just couldn't bear risking that more would come to the world I just don't need to do that so I would alwas have them neutered so I know that I'm not goign to cause any more unwanted lives anywhere

Participant 4

I have both my dogs done as well my son was heart broken when I got the great dane done and i told him that when he is a sleep I'll bring him down and have him done as well, but I actually found that it did calm him down as well though, because he was all ove rthe place he was like a donkey like, there a very nervous breed the great dane are, and the vet was actually worried when he seen the great dane because you know Participant 4

if he just taps a little one he could just crush him (other dog) to death but the little lad is the boss. and he's 13 now so the young fella wasn't impressed wouldn't talk to me for about 3 weeks and every time his friends came in and said hi, he said don't talk to her see what she done he didn't pay the bill obviously...

Participant 9

my views on neutering changed only recently in the last 2 years em I was quite against neutering my dogs previously they weren't neutered am me and my mam just believed as they weren't going to be out wondering on the road they wreren't going to be in any accidents, they weren't going to fall pregnant, so we just thought why not let them be just natural and they are, and we had always done

that and then unfortunatly on my last dog kess we didn't get her neutered or had no intention to but she got to 8 she developed a lump under her arm or 6 months and I was struggling with another vets, too and from ah every 3 weeks bringing her back and I was insisting that i thought she had a tumor mammary galand, I was sure that was what she had and she was leaking blood and all the rest and the vet was insiting that it was am hormones because she hadn't been neutered, so i went along with the whole process as she was getting sicker and sicker they insisted that I get her neutere as it would change the whole hormone balance and this was probably right em got the dog neutered, and a week later she died I know that the dog had cancer I could feel the lumps around her, you know you know. but she had been sick for 7 months and it was never an option for me to get her a biopsy or anything like that and I got very negative towards the vet and about the situation, long story short, she passed away and since then I never want to have the contradiction again of going into a vet and saying she's not neutered it could be hormonal so the 2 dogs I have now, pepper got neutered at 6 months and dougle is booked in to get neutered, I just never want to encounter that again, thats my view now on neutering

Participant 5

My dog is neutered and the reason for it is he's a cocker spanail as well and they have a reputation for being quite hyper and they say that neutering will calm them down, and also as well we live in a terraced house and he can't get out but I would just be affraid, I don't really know what happens when dogs go into heat but I do know apparently that the boys go mad so I just thought it would be safer, as we try and walk him with out the lead on Kiliney hill and I'd be petrovoid if he just ran off he left me I wouldn't know what to do, so I would agree with neutering and I guess it is more I suppose for my personal situation just I would be afraid that the dog would loose the run of himself. I suppose he's my first dog I don't know any different but thats what I would think of anyway.

Participant 8

The animals I had were always neutered or spayed, we kind of didn' teven think of it we just did that and when we got our current moggy from the DSPCA I think on policy they automatically neuter them that kind of is the reason, and I thought that was good, I the thought of having to watch them giving birth and having to get rid of the kittens would just be awful, so for me it is just a practical thing and also one of the cats I had when I was a child was a tom cat that adopted us, he was discovered as starving and we took him in, I think it took us nearly 2 years to get him in and all the rest of it and you could see a deffinite calming in his behaviuor, he was still an aggressive little bugParticipant 1¶

to be honest, but the, he used to fight alot and the things you hear about dogs in heat this tom cat, talking about sowing his wild oats he definitely lived a very active life untill it was done and it calmed him down and his behaviour changed, I suppose some people would say it was a bad thing, but tippy lived untill he was 14, and it seemed to have a very positive effect and to me it just made sence because if the cat has kittens your going to have to find a home for them, I've nefver actually had a cat that

hasn't been neutered so thankfully I've never had to go throught that, but you know they can have several litters and persumably that can't be good physically as well

Participant 3

I think neutering is alot about responsibility

Participant 8

So do I,

Participant 3

I've always had the cats and the dogs, female dogs neutered, but I really do think responsibility is the key word there, because you just cant place kittens and you can't place pups either and thats not just since the recession it's going back years or else people who took them they weren't responsible people, you would have known if they'd still have them in 6 months. I think it is just an easier way of just handling the whole situation.

Participant 1¶

I think the animal is happier as well, never having a dog, a firiend of mine had a female dog my god what that poor dog went through, it was a bit like a sheep dog phantum pregnancies and all these things, and I couldn't get over that they never got her spayed, you know she was going through hell, I mean up untill she was 8, 9 ,10 maybe 10 years old 12 before she died she still went through all the hormonal stuff that men an dwomen go through and I thought it was cruel, absolutely but I would always neuter as well, because of the constant kittens and all that but I mostly had male cats for the last few years, and there much happier, sure they'd be out fighting an d there can be an aroma, to say the least, for themselves and for everbody else as well I suppose, you know they'd fight and they'd also be out and there much happier I think, and their hormones, I think inside they must be much happier. you know.

Participant 9

I don't know if they are you really

Participant 1¶

Do you not, they still have a bit of desire and they still go out and they won't do any damage

Participant 9

I think if I didn't have that expierience. and just from having dogs that haven't been neutered I have never found them to have any hormonal problems or anything like that if I hadn't have had that difficulty/dissagreement in the passed I think I wouldn't have got my dog neutered, I can completely understand male dogs because they can be quite aggressive sometimes if they're not and if other females are in heat so I'd be more sideing towards male dogs but I have mixed views with neutering, just because ... it is nice to leave them and not play around with them too much just leave them as their natural self and especieally I can understand with cats cause they're out wandering and stuff, but with a dog and you know where they are all the time

Participant 3

But if they get out the once you know itsa all it takes, and it can be a very very small bitch and a big dog that can be very complicated.

Participant 1¶

And if they are in they are very anxious to mate, you know it depends I suppose on the personality of the dog, but its in them its inate to mate and to have that feeling and probably phantom pregnancies aswell the drive is there for them

Participant 3

I think with cats as well the important part of having a pet is you want the companionship you want them there and a neutered cat stays around the house they don't wander and I would be like you I don't let them out at nght they're called in sort of thing and the are indoor cats that are occasionally go out thats kind of the best way to describe them, when the are nuetered and spayed they do have there own little routine and thats it you don't actually have to worry about them wandering so it's like a self forfilling prophesy

(laughing and cross talking)

Participant 6

The dog we have at the moment is not neutered, but I don't actually have strong feelings one way or the other about it, I think it depends on the dog and the circumstances under which it lives, whether you have a garden whether other people are home during the day whether the dog is taken out on the lead only or what ever, so I think it depends sort of on the character of the dog and we've had some that we've had neutered and others we haven't. adso I don't have a paticular y strong view on it, you know, so that would be where I'd be coming from yeah.

Participant 1¶

I work in an area in rathmines and ranelagh and its Dublin city council sheltered housing and they're over run with cats now they're all georgeous an dthey're all b;ack or black and white, there is only maybe one or 2 tabbies and theyare all just intermating and the odd one will be caught I've neutered one hterre a few months ago because she was about to go into heat again and she had 5 kittens and they were just running wild, and some man had brought them in she'd actually had her kittens in his house and he had kept them in over the cold winter and of course they were 5 months by the time I got to them which was about march and they wrere wild and doing their toilet every where and he just couldn't cope anymore so at least she got neutered and the kittens went off to stocking laane to be rehomed but you know they're all you know their eyes, they're not well looked after

Participant 3

Its all about the early stages, you know it that first 6 months I think they have a predisposition to certain conditions

Participant 1¶

And they die off and they're just dieing you know you just cant save them all you just can't. I can't financially neuter them all or even get them, they go beserk some of them are quite wild but they do die they're beautiful, black you know it would be better if they could be caught and neutered and that sort of thing.

Modulator

Lets move on, so what influenced your decision, media vet.......

Participant 3

Common sence

Participant 1¶

Past expierience of unwanted pets

Participant 3

Your the one that has to life with the animal, so you know you can't be looking after new sets of kittens every few months and new pups and trying to re home them so comen sence I suppose I mean what ever it is you pay it the once your done your done don't even think of it afer that. couldn't be easier from that point of view

Participant 5

I think as a first time dog owner, my own dog I think a lot of it has to do with what people say to you as well am the vet would recomend it and people who have expierience will let you know what their opinions are as well I suppose I've come accross more people that are pro neutering that would neuter their dog so I think that would have a lot to do with it I don't know if it would be called old wifes tails or myths aswell but you do hear certain things and you kind of go oh right I better do that then your dog is going to go sort of crazy or he's going to get cancer or you know you hear certain things like that that would have influenced me

Modulator

Any one else ...

Participant 2

I think every time you hear any more figures, how many pets animals dogs and cats that have to be put down every year, because they can't be kept, the shelters are all overrun with them its just the tought of it going on is just horrible I can't imagine not neutering thats why I would do it, unless you specifically want the animal t breed from its got to be neutered

Participant 7

Country people doesn't believe in neutering they say it's going against nature, but so is drowning the kittens going against nature.

Participant 2

So you become a murderer instead

Participant 7

They think it's going against nature getting the cats neutered but still they'd drown the kittens and there is nothing at all about it

Participant 1¶

And they laugh at you "city girl" you going to the vet and spending a lot of money on it

Participant 3

I think a lot of it comes down to the money but they won't use the words money

Participant 1¶

yeah maybe, probably.

Participant 7

I don't know because there was a case happened down in Ballycarney a couple of months back and there was this box on the road and there was a couple of air holes in it, and there was a litter of kittens in it and they had been run over by a car, the mother of them kittens wouldn't she have been far better off if she had of been neutered

Modulator

(Tea break)

Modulator

Just your attitudes towards pet diets......???

Participant 7

Well our dogs get anything thats going, I usually give my fella the red mills, chicken and fish, the little munchies, then we often get normal stuff for the rest of them and they get whats left over after the dinner. and if you get rabbits or anything you boil the rabbits and give them the meat off of the rabbits and the stew and that they get a mix I buy the cats food, mostly give them the dry food for the cats but often people say that the dry food isn't good for them, well they get the royal canin thats the one they get now.

Participant 1¶

Yeah I think it must the supermarket one (that their talking about) it's full of salt and stuff and processed I believe in these ones here (Food in waiting room)

Participant 8

I always buy the comercial one, my mam used to by whiskers when I was young and they'd get a whiting once a week, which is the patern I have replicated, but when I got this cat I was asking in the shelter about food and they said the stuff you buy at a vet practice is better so I immediatly todled off to the vet and its royal canin I'm on and I have to say they seem to be thriving but the still get their whiting fillet once a week as well. You do what your mammy told you to do, they we she delth with the cats is the very much the way that I do, and I cook it which causes histerics amoungst my friends but I always get them comercial stuff but they do seem to be fine with it

Participant 3

I give the westie redmills chicken and fish agian because it's a favourite and the cats then I give them whiskas and t/d because I have an 18 year old cat and to keep her teeth an dher gums as good as possible because thogh she's had her teeth done some years ago on several occaasions she's gone too

old to be putting her under for that, so they both love the t/d and the whiskas and the milk and that and nothing in between for the dog, I think with the dog it is very easy to slip him a treat here slip him a treat there and you hear people say oh I had a chocolate biscuit but I didn't finish it so I gave the rest to the dog and I had a neighbour who had a sweet shop and she had a westie and he was a little bit of a spoilt westie because she use to say he never left them leave the house without them giving him a bar of chocolate, (talkign laughing)

Participant 7

Well you talk about a bar of chocolate my teddy has been diagnosed with diabetes that was in januarary and in march there was a big box of roses with 25% extra in it and teddy ate all of them but 3 sweets left in the box,

Participant 4

I get the food here for the great dane because if he got the other stuff it was just running out of him, it was just too much cleaning up, the vet said to me this one would help, because he was like a horse I used to hate getting up, so he can only have the one type, now the chances are he will try to get to the smaller lads but I always know because it'll just run out of him like, 80% of that food would just run out of him, so he has to get the special one from here. The small one now he used to go up to my da,s so he was spoiled , grilled rashers, I used to say I'll come up and get patch now and he'd say oh yeah Participant 4

he perfers the boiled sausages this week and chicken. and I remember coming down with his girlfriend one day and the vet said right Participant 4

look at all the weight he has put on and I said it was nothing to do with me, it's me dad they used to give him everything, steak the whole lot, and also he was spoiled and he was getting fatter, but the great dane now I would have to say he could poo for Ireland. he was just beyond a joke, and I'd say to my son he's your dog and he'd walk out and he'd go no mam I can't its discustin and he'd actually get sick, so when the vet came up with this food it was a big difference because he kept it all in., but if he went near any other food 80% would just run out of him.

Participant 9

Growing up we always had dogs and my mam used to spoil all our dogs and all I remember from growing up is all my dogs being extremely fat, we had a jack russel when I was young up to my teens, and he was fed 2 lamb hearts every day cooked every day, and sausage for the breakfast and and proper nicer meals than I would have and he was round and fat but lived till 12 years of age he had the best life

and my mam is a firm believer of spoiling them while you have them so her way of spoiling them is by feeding them and she always says they didn't have food when i was growing up, so now I can afford to so why not do you know, then we got kess and I was a bit more strict I didn't want ot be walking 2 fat dogs so i started to tell her not to feed them then we were told by the vet that there was problems I've been feeding them the science plan but my mam is like when the dog isn't eating the food, he doesn't like that food and then gives them all the bits from the dinner, so its very hard for me to try and get them in cause when I'm at work all day I try and get them in to feed them and I know when my ma has given them food cause they won't eat theirs, so I feel I'm fighting a loosing battle with my mam loves to spoil them. I'm trying.

Participant 4

It is it's very hard when somebody else is involved and is still actually giving them stuff even after you tell them no that the vet said not to and even see when my friend comes down and i see her buttering bred and i said to her I'll make you a cup of tea to go with that, she's like no its for the dog, and then she's there I hate to see a dog scrounging for foodat the table when your eating so now I actually have to put him out when we're eating. and it's her fault cause she keeps going under the table with food.

Participant 9

He is only 4 months and he is at the table every minute of the day and I know it is because my brother, my ma and my dad are insisting on giving the bit of chicken or the bit of skin " ah look he loves it" They believe in the dry food and they know its great for them whatever but they also think they love the nice stuff too much so why not give it to them you know.

Participant 4

They hate when I put the dogs out their like ah george, I say no because the great dane would actually put his head on the table you'd have your knife and fork there and his head would be right on them. Its very hard when you trying to do it and someone else is spoiling them

Participant 5

We use the dry food, it was recomended not to give the dog the tinned food, wet food I don't think it's ment to be great for them so we always use the dry food, but I would be terrible, we'd go for our walk in the morning and when we get back he gets his slice of toast, Then I give him some treats when he goes outside and then when I come hiome he getshis dinner and he gets some more treats I'm

a big believer in treats to keeop him happy. but there all the dry kind of treats and he gets those rawhide type treats as well and different things to chew on and things like that, but he does stick maily with the dry food.

Participant 6

Well we'd feed ours a mixture of the dried and tinned and she does get some tit bits from the table, she deffinatly has a sweet tooth, you know a biscuit or something and she really lick her lips at that, she'll eat her ordinary food but she would love some other stuff. We were told to keep her down to 2 solid meals a day and she gets an odd bit in between she's happy at that and it doesn't cause a problem, she wouldn't be hounding you for food, either, you know what i mean. she seems to be happy enough with that, she's not putting on any sort of weight or any thing like that, so as long as she's sort of healthy with that we'll leave her be

Participant 1¶

I have a young cat and an older cat of 11, so he doesn't eat very much but he is huge he is like 9 kilos, but its his make up too, he's got big paws and everything, They tried here putting him on a diet and everything but he has a urinary problem and he has to be on special food for that, he suffers from cystitis so he is on the urinary food, so the urinary food is high in protien or something so it's give a litte bit of it, I did try for about 4 months but then the bladder went haywire even with the the extra tabblets and he was hungry and that was awful, no he needs to go back on the urinary stuff. I think he has lost weight now he's back on the urinary, I don't give him as much now so maybe he's lost a little bit of weight but heis a big cat but he gets nothin gelse cause he has a sensitive tummy, he'd love milk he'd love ice cream, he'd love any ham , cheese, and dairy sort of.

Participant 3

My cats love cheese, especially the 18 yo he loves it, i think its great to keep the weight up he's such an old cat and there is plenty of fat in the cheese and he loves it, and he has a specially meow he gives when he wants it, he gives this meow as much to say go get it NOW,

Participant 1¶

so he's on that, but if he has anything else it just runs through him, really , so he's really just on boring same old stuff the whole time. it is very good for their teeth I think anyway. The other one he came to me he was up in the tree or something and he immediatly went on the dry food as well. but I do, because he won't listen to his mother and he won't come in and he's a boyo so the only way I can

get him in is by tuna, I only give him a little bit, maybe a tablespoon ful, he gets that quite often, tinned tuna, normal that I would eat, he doesn't get anything else no milk or anything like that, it's not bad for them is it. (laughing......) I need to get him in cause he goes out the front and his days will be numbered, thats just the personality of him

Participant 2

Well my mother used to feed the cat on cod, which she used to cook for them and fresh chicken and there was a little bit of whiskas or IAMS or something in the background, but she liked the fresh food and I swore I was not going to go down that route, being the cheif for the cat all the time so the tabby lives on whiskas, he gets it dry and some cParticipant 2

ed and his very happy with a little bit of fresh chicken his coat is very healthy he's fine, the dog had a lot of skin sensitivity when she was a baby and got tummy upsets and things and seemed to be very sensitive, so it took a while to get her diet sorted out and finally by 6 months and is on Janes well beloved organic food, mostly dry, but to get her to be tempted to want to eat her dinner you have to add maybe a desert spoonful of the wet fod as well. occasionally when she knows I'm going to feed the cat chicken, she'll only be kept quite if she gets a few flakes of chicken as well. and instantly she eats her dinner. she thinks she's getting what he's getting so she's happy. but I try not to give other food the only time I might slip is she does get a little square of toast in the morning, when I'm having mine she sits beside me waiting for her bit to be given to her, I try not to give her any but that is her one regular treat that she gets, but otherwise it would be just her dog biscuits that she would get, and when I'm going out and leaving her behind it's standard practice, she has to get a boneo to mind the house she knows I'm going without her and she doesn't atempt to go with me or get excited and she hasn't turn the house up yet so it must be working . she tends to be a little bit chubby, but I think the cockers do tend to be chubby, just trying to be good and keep it down, but she gets a lot of exercise plent of swimming and running on the beach every da I hope thats a healthy life for her

Modulator

so Kind of runs on, so what factors influences the weight of your pet?

Participant 1¶

I would have been inclined to jusst to put out the food and just give a big dish of dried food and thatt'll do them all day just top it up. Cats are different they'll graze and they'll only eat what they want to eat, so maybe I've learned a little bit over the last year not to put a whole dish down just to put a certain amount out and just keep an eye on it. I would have been just leaving out too much of that dry

food for him. The other fellow is different he's skinny and he's a grazer so I just leave a whole dish out for him and he's happy with that.

Modulator

How do you keep them from eating each others food?

Participant 1¶

no they're seperated, the older fellow is in the kitchen and the back and the other fellow is goes out as well but he is in the rest of the house, they would fight. He does get at the younParticipant 1¶

cats food every now and then but not enough, very rarely it would be my fault that he is over weight but he's improving.

Participant 6

But I think in the past we've certainly over fed the dog an d Ithink dogs and from experience if you feed them they will just eat it so twice a day is enough and a little treat hear and there if you put down 3 or 4 bowls they will eat it, I think they may eat it untill they get sick. sometimes you make a mistake someone hasn't told you that you've fed them. and you feed them again and they will still eat it. so probably in a household with one person just doing it is probably better, otherwise there is a tendancy for them to be fed or overfed you know, we have seen results on one dog in paticular that ended up carrying quite a bit of weight and as she was getting older it was impossible to loose it so am sort of learned that lesson not to over feed, your not doing them any favours.

Participant 7

Teddy he's a diabetis and then dodParticipant 1¶

he's the pup, they're the only 2 that get 2 feeds a day, the others only get fed in the night and then they get a boneo in the night when I've the other 2 after feeding , teddy has to get fed twice a day on account of getting insulin as he's diabetic, and then the pup had to get 2 feeds a day. any he was quit thin when we got him, (over talk) I only look after teddy there all in the garrage and they have the run of the garden and they sleep in boxes in the garage they all have their own, teddy has his moments like people with the diabetes he can be a bit grumpy , he has his grumpy moments them 2 are the only 2 of ours that get fed twice a day, the rest get fed once a day the weight is great cause they walk, me husband could go out for hours with them,he take 2 or 3 out at a time I usually take I take teddy out first in the morning and then when I come back and then I go off with dodge and then my son might take them of

that evening but my husband would go out twice a day with them up kiliney hill and around he's a great walker, and my grandaughter now she's 18 and shes from wexford, she was up a fourtnight ago and Joe asked her was she ghoing out for a walk with him and she said of course grandad and the had 10 with her joe had another 2 of the dogs they headed off anf Joe would be talking to her and she stopped it was so embarising imagine I'm not able to keep up with me grandad.

Participant 3

I feed the cats twice a day morning and evening and then the westy will just tend to eat out of a bowl of the redmills, which is just there for the day if he ws a greedy dog I wouldn't do it that way but he's not he just takes what he wneeds and thats it, his weight is standard, the cats, the 18 year old is a slim jim, and always was a slim jim, and every so often she'll drop a bit and we have to concentrate on bringing it back up again, that why i think the cheese is great. and of coursefull fat milk or ice cream or what ever is going. the other cat is about 6 and she was neutered and since she was neutered she just went out really. It's very difficult for us to seperate there food, our garage is like a utility room and thats where they live because theres a cat flap in that it's the only way, place we can have a cat flap, am so it is very difficult for us to seperate their food, and we can't do it in the house caues we have the westie in the house, the westie used to chase them, now the westie is not bothered now that its 6, but they still really think lets keep our distance you know, but there isn't a problem at the moment with their weight but certainly we would watch them and its just standard food, as such, well the td is for the elderly cat she takes the td as well, which is no harm for her either , and then her teeth aren't great either cause she was a stray that came into us at a year, she had been on the road for quite a while, and the enamel didn't form properly on some of her teeth, so the td is probably quite good for her,

Modulator

I would do you think your cat is skinny at times?

Participant 3

well because he is 18 and I suppose he has the Participant 1¶

iatric profile in the sence that people when they get into the really senior years start to loos e weight and at timess his weight has dropped so we're just concious of feeding him paticularly carefully to keep it up at a respectifull level, but i mean too much, he's a slim jim at the best of times,

Participant 4

I feed them twice a day but patch only eats about once a day, patch is like a little gentle man, you'd love to give him a knife a fork, but if you feed him outside the birds will come down and they'll eat chinos food, they daren't go near the small dogs because he'll kill them he chases them, the great dane wouldn't do anything they'd even sit on him while there eating the food thats how really stupid he is my son would say to me I think there is something wrong with him. last week there were 3 birds sitting on his back eating his food and he wasn't moving and then as soon as patch went out he tore after the birds, but it doesn't bother the gread dane they can do whatever they want. twice a day they get fed . (and there weight is OK Modulator

) well patch wouldn't have been with my dad lord have mercy on him, and his girlfriend, em chino goes kind of up and down he lost 3 Kg the last time he's still like 8 and half stone, I tell hime not to worry though I'm still bigParticipant 1¶

, he knows if I give him the look he's after doing something wrong.

Participant 1¶

Would that be the normal weight for a great dane, I know there big but is that normally the weight

Participant 4

My sons friend has a great dane and he actually feeds his one 2 chickens a day (o god, gosh by others) like he's big and the head is huge, my lad is like a gentle man jim walking down the road beside him.but his weight would go up and down I suppose if you were to give them as your man 2 chickens a day you wouldn't be able to handle them at all like cause he's a house dog like he doesn't sleep outside or anything, they sleep inside and if patch goes on his little holidays up to my fathers girlfriends house then he leaves the great dane on his own so the little one I have fostered says ah look he looks very lonely she takes him up to bed so shes on one side of the bed and he's on the otherside and then he'll pull the duvet over himself and go asleep. he's better looked afterer than I am no he's a grand dog but he's huge likehe scares people they just look at him and go "hhhuuuuuhhh" and he sits on the sofa like you or I watching tv, i think because of his size he can't sit like a normal dog so he sits like this (demonstrates) on the sofa it's hilarious lookingat him like. the small dog though is still the boss he's 13 bhut I think the great danes life span is very short I think its only 6 years , my fella he is 3 and a half. some say 6 to 7 is the life span of the great dane, now, but if anything happens by the time he reaches that age the other dog will be really old I'd say both will go at the same time and I told my son not as much as a goldfish. no I'm finished now. (talking over each other) I tell my sun he says its his dog when people say how lovely it is but if I go what did the vet say today "I don't know"

Participant 5

("How are you getting on with your fella", Modulator

)yeah he is grand, the proper weight I think, he's not fat he'd get fed twice a day and them he'd get extras, but twice a day is his main meals and he does exactly what you were saying, he would eat anything at all, he'll eat it, the only thing he doesn't eat is grapes, and oranges. He's tried everything. he is a healthy eater but he will get sick quite easilly if he eats too much food, he'll get sick, but then he'll also try and eat that, am he's fine his weight is good.

Participant 9

The schnauzer is extremely fussy eater I have to make sure that she'll put on weight, she's been under weight since we got her just purely down to the fact that she is extremely fussy and nost dogs will take anything out of your hand that you offer them, pepper wouldn't eat a sausage, wouldn't eat steak wouldn't eat meat, chicken is her favourite and the one and only thing that she'll eat over and over again, we tried her on various things she just turns her nose up at them she just doesn't want it, there is nothing wrong with her she just doesn't want it, so she loves chicken and she eats the wet food and mixed with dry but its hard enough to even get it in to her she needs to be starving to get it into her, this other guy is the oppisite and we need to be carefull that we don't over feed him, because he would literally eat anything. with pepper we're not too concerned about her gaining a bit of weight because she could do with it she's up to a weight now that we are happy with but I could immagine her just not eating if she was in a bad mode she just wouldn't eat her food and ah I think exercising is the biggest key to keeping the weight down, I think you could feed your dog till the cows go home, aslong as you bring them out for their regular exercise it definitly makes a big influence on their weight you know

Participant 8

My sister used to have an enourmously fat cat that looked like a torpeedo and I was always paronoid that of having fat cats and it was the vet here that actually told me that you should be able to feal the cats ribs, but you certainly can't with my 2. they're not overweight overweight, but they are slightly pudgy cats I'd say. I feed them 2 and half time a day, and that seems to be fine they both have that round cat shape when they are sitting but I, I suppose with a pet because they're not having to hunt or anything like that they're naturally going to be a bit more prone to being carrying weight cause they're not hunting for their food anymore am a cat because its smaller if you have stairs in the house that is pretty much their exercise there runing up and down the stairs, you can kind of see it, you know what i mean, they're only little there not like a dog or anything, you can see it, if your kind of keeping them, cats I've had have always been like that, they'd be trying to follow you around the house, and kind off and you'd be upstairs and the cat would kind of majically apear around the door to you, so my cats are are sedentary to be honest, but they potter aroung the house, messing, running up and down the

stairs, but as I said they occasionally go outside and watch the birds and come back in am but they certainly wouldn't be skinny now.

Participant 2

My tabby is a 2 year old, he's a big strong muscular cat he's certainly not overweight he was here recently getting checked he's just right but he takes a lot of excercise, he's prowling a lot, and I feed him twice a day but there is usually some of the dry food in the dish, and he'll come and have a nibble now and then but he doesn't over eat he just naturally seems to know he doesn't take what he doesn't want. the dog honey, well she's not that keen on her own real meals, they can sit in her bowl for a little while, when I've prepared her dinner and put it down, she's lying watching me do it she doesn't get up to come and get the food, she's not fussed at all for a while she'll pay no attention, and then shed go and eat it, but she will eat anything and I don't mean food, she literally eat anything, I mean she'll eat her favourite is plastic dishes. I allow her to have a new plastice dish that I just emptied the vegetables out of or something while I'm in the kitchen and once it starts to break up I remove it. in case she'd swallow a piece of it because she would, she's had very strange things go through her in her puppyhood; a pop sock, it took 2 days to reapear, managed to stop her eating the tights, she did need to be helped with the pop sock, but luckily I was there and able to assist, she survived that one and she eats all sorts of rubish, she ate all my flowers in the back garden I've no flowers in the back garden. only have hanging baskets ths year, I didn't even bother to buy others this year, I'll wait till she's older hopefully she'll have calmed down I think she is calming because she's ignored some of the flowers that came out this year that I didn't plant myself they just came out. maybe she's improving. she'd grab anything off the table if she could reach it be it food or otherwise and half the time she doesn't want it. but she wouldn't eat till the cows come home, she'd only steal, thats what she likes to do. she's a born thief. if I'm cutting up vegitables she has to have the end off a piece of carrot or something, she likes a lot of ifferent vegetables, except celary and onions, she gets fed twice a day .

Modulator

I want to get feedback on your pets exercise routine. ....

Participant 8

I suppose playing the cat gets skitish and they'll charge around after a screwed up piece of paper or a ping pong ball and that kind of thing, even though my cats are elderly they still do that which is kind of nice, I would see that as a good sign that ther healthy and as I said they're up and down the stairs but we wouldn't have an exercise routine per say with your cat and I think a lot of the time if a cat is over weight is to do alot with the size of the portions you obviously try and catch them and stop them eating

and leaving the food out with them, but I'm not sure can you have an excercise routine per say with a cat.

Participant 2

I suppose you could try and play with them, my father used to play with the cats before bed and they had various toys, and just before bed they new it was time for their games and he'd come out with the toys and throw them around an theyd interact with them so I think you can do, it can happen (an excercise routine) I don't know if it's specifically for excercise more entertainmant of the pet human I think, the dog one of the reasons I got her was to make me excercise, I knew that she would need the excercise so if she's getting it I would get it too. and thats worked except she hasn't got me in swimming yet, its too cold. I envey her going in every day, goes straight into the sea and swims. I do try to see that she gets a good hours excercise every day and if not 2 shorter sessions I try to see that she gets plenty of exercises, if she meets other dogy pals, thats the best, cause she runs with them and gets 10 times as much excercise as her walk. I hope she will still be like that when she gets older, I think as they get older they tend to play less, when that happens I'll cross that bridge when I come to it , but I try and keep her excercising and keep me going too.

Participant 1¶

I don't really have a routine with them, the younParticipant 1¶

moggy is flying around, but with the older fella with the weight problem, I have loads of balls and these things, the long furry thing on a stick, so I do a little bit but not every day or every night. I have to brush him every night because he's like angora, but thats about the height of it. But we take mice out and balls and thinkgs in the kitchen and so he does play with things himself in his own time, and he'll run up and down the garden, some times, other times he'll just walk so, I don't really have a routine of exercise. or a programme of exercise, it's up to him.

Participant 6

I don't have an actuall routine, I would be the only one that would take the dog out, for a walk per say, there wouldn't be anyone else who would have the time for it. But we do have a large garden, so she deffinatly prefers to be out rather than in, and am she's a bit of a silly dog, she'll chase birds anf pigeons, and but once anyone is in the garden she'll be out and she'll be marching up and down and she'll go around the whole territory, but she really likes is if you take her up to the park where you can actually let her off on a lead, and hopefully another dog will appear and just run around and thats really what takes the energy from her and you don't feel walking her on the lead is enough, for the sort of energy she has so the freedom of the park and for her to just run, and it also means you don't have to

walk as far. She is a bit strong on the lead and she has never really copped on to that, we never properly trained her so sh edoes pul a bit, especially if she sees other dogs soit's not the most comfortable thing, and she doesn't get that much excercise from a walk. ideally it is to take her up to the park and let her loose. I wouldn't have that situation where I take her every day or every second day it is just purely dependant on me, so thats sort of an advantage, I can come and go and she's not looking to come with me every time I go out the door, she does know once I've taken out the lead, I'm going out she then gets a bit excited. I think overall because she likes to be out so much she gets sufficient excercise.

Participant 7

Did you ever try a harness, I find it helps them from pulling, because I used to have a lead on my fella, and he used to get out of the collar, no matter how tight I had the collar on him, and he's not the best of character to have loose, he runs after jogParticipant 1¶

s, you'd be affraid that he'd tear them, he'd be jumping up at them, they might say that he bit them. He likes to be with some dogs especially one, but after that he barks at them, he barks at anyone anyway, but thats another reason why I don't take the other fellow with him and me, cause I don't want him to get into the same habbit as the other lad. because he's too old now and I don't want them to be mixed and 5 or 6 years I'll be watching another fella the same. and account of teddy not being well I woould have him off with an ordinary dog, but he's not getting out of the lead, sometimes there is a couple of dogs that he likes and if he gets with them he is happy but the pup now is great, he's a real friendly he goes over to everyone, and he wag the tail and sit down beside them and real gentle you'd wunder how any one could have done what they done to him, he's a real soft yoke a real little face on him, cross between and alsatian and something, he's about 6 months old, but the others are all small anyway, and they're all mixtures of terriers, you don't have that much work with them anyway, you pet the lot of them you feed the lot of them , you give them their boeo in the night and they each have to get their own attention. The cats then look after themselves, you leave the dry food out all day and they eat that, they go in and out through the windows as much as they want to but they're old, I think it is 16, 12, and one of thems 6, when you'd be in watching television the'd get up on your lap, thats what one of loves getting up on your lap. they old one, she suffers from stress, she used to be in the kitchen on a shel, she'd sleep on the top and you'd feed her on the bottum, shes happy now,

Participant 3

I have a westy and she insists shes going for a walk, when I'm working I walk her in the evening, and when I'm off it has to be after breakfast, she'd be happy even if I walked her in my dressing gown, she's a bundle of energy and thats of course like all dogs once they're walked , they're much calmer for the rest of the day. We would also have dogs behind us, and when the dogs would bark, he would go off like an ape, through the dog flap, like a rocket, and flies round the garden, she knows she can't get them but she runs around in circles, several times a day, so she gets plenty of excercise, so the cat only gets

out of the basket, to go to the food tray or out to the garden, the other cat is gone must of the day, I know she goes to my mothers wherelse she goes I don't know. Everyone says how does the cat know it's your mothers, I have to say I don't know.

Participant 4

My 2 dogs would be running around the back garden all day, while we're out. and then my friend, she'd come down and she'd have them out for a walk, if she had her way, she'd have them out for about 7 hours, she actually loves bringing them out. Great dane you have to watch because if they tumble they can break their back and thats the end of it.in mmy garden there is a wall, all my plants are gone, because he jumps and he just keeps killing the plants, i need to watch that (his back) for when he's jumping over the wall. The y go up to Duneen and they just run arround but they stay beside each other and the small lad is like a little rabbit the way he jumps the way his legs go he's like a little rabbit, they would get a good bit. The great dane if he's after been up in the bed with leara, and she toddles off to school, if I was to leave him he'd stay there till 6 o'clock and woouldn't move, wouldn't wet, wouldn't do anything. he would stay in that bed with the duvet and wouldn't move. I'd shout Cino and he wouldn't move. I'd say to the young lad, will you get the dog and he'd say chino come on come on, but no, I'd have to go up and give him a look, and he knows when he gets the look he's going to get in to trouble. So fuuny one day I told him he was grounded, I actually forgot it was the dog I was talking to, My son was laughing at me, I think you've lost it. But he would stay in the bed all day if you left him. he is so lazy it isn't funny. but then She'd be out for 3 or 4 hours with them and they'd be flying around.

Participant 5

We have a routine, first thing in the morning we'd go out for a walk before I go to work, and then when I get back from work we go down to killiney and we go for a swim as well. and sometimes my husbands parents will take him out for a walk. But the routine is twice a day and he has to have it himself, he'd go mental otherwise, he knows, I think since we've always done it since he was a pup he knows the times and is ready to go when ever your ready to go. He's good like that, he enjoys it,

Modulator

The last point, How important to you and your pet is the time you spend with them

Participant 5

I find it's important when your excercising them and stuff, they learn to listen to you, to see that your the leader of the pack in a way as well. when they are learning to come back to you, I don't know

my dog seems to take me more serious cause he knows if you feed them and walk them he knows that your the person to go to, I think it is very important, the excercise and especially talking to them. I suppose it is like a little baby. I would think it would be very important.

Participant 2

I think it is very important to have lots of contact with them they're part of the household, part of the family. the more you talk to them the more responsive they get. And that is really applies to most cats too, my present tabby I do miss the closness I had with the siamese & burmese, they're very affectionate and they love to be picked up and held, and cuddled and everything, the tabby he was rehomed for the second time to me so I think his life had a fair amount of trauma in it and he doesn't feel very comfortable being picked up and cuddled. He'll tolerate it but he doesn't really want it, so he is at his best if I get into bed at night and he comes into the room and pops up on the bed, thenm he'll come for a bit of affection, enjoy being stroked, but it usually turns into a game killing my feet through the blankets. he is still very kitteny, but I think he is getting more affectionate as time goes on, I think it is good for both of us, certainly for the dog, it was vital from the word go, that she got plebty of cuddles and contact and a lot of conversation, because she's certainly turned into a very loyal companion, and I'm sure thats partly because she's being treated like a person, if you like, and it helpsif you take them down to the beeech and take them out walking, they're much more inclined to come back to you and be with you . my one hates to think you'd be going off with out her she'll run around with her friends, but she'll sudenly realise that " oh where is mummy" and she'll look round and if she can't see me she panics. she needs to know that she can get back to me. Which is no harm as she has no tendency to wander off. She's very good about going home and getting back into the car, so it's good for both of us.

Participant 8

I think like that you get animals for companionship so obviously I think it is very important that you spend time with them, as I say I don't really excercise them, but I would have a lot of contact with them and my second cat I got through sad cercumstances, there was a bereifment in the family and I ended up getting the cat. and the cat was already , a bit like your great dane, we always suspected he was a bit soft in the head. She was a damaged cat that my sister had, the cat was always a bit neurotic and I kind of, because she was my sisters cat as well, there was a lot of emotion and trauma around getting her, but she's coming on, she's starting to relax a lot and I think its very important because thats the point of them, they're ment to be your companion and their part of your routine and your household, I would have conversations with them, if I'm trying to get something straight, I would talk to the cats, so to me they're a psycological prop as well, I think it is the most important thing of haveing an animal, whats the point of having it if it's not your companion.

Participant 3

You do feel a bit mental when your talking to an animal, They first time my sisters cat decided to do a runner on us, and didn't come back my unfortunate father had to stay at an open window untill 3.30 am and she still didn't come back untill 20 to 9 the next morning and swParticipant 2

ed in with the where's my breakfast face and i practically picked her up by the whiskers and I said don't do that again. You forget, that. They just become part of your house I syuppose. So a lot of touching and talking .

Participant 1¶

I would be the same I would talk to them and that, but I would be out all day, they know my routine and they have their routine, I never go to bed..., one fella is in the kitchen so he gets brushed and chatted to every night and then the other fella he will sleep up in the bed. But he's running around all night. yeah but we talk and I give out to them as wel if their; out the front, I coacs them in and then give out when I get them in "what the hell are you doing out the front" because he's a bit wired that young cat. I suppose I'm quite enough in the house but they know me by now.

Participant 6

Ours would be very much part of the house, if she's not there the odd time she's had to be kept overnight at the vets, you feel this sort of void in the house, its hard to put your finParticipant 1¶

on it but you do miss having them in the house. Even if your there on your own and you go to bed or whatever it is good, cause you are aware that there is somebody else in the house. As regards sort of recognition, I just bring her out and let them run around and I whistle and the minute I wistle the ears pop up and depending on how far away, I think her sight isn't that great but its your sound that she picks you up and she comes at a gallop, and I just put the lead back on her if I'm taking her home, but we have a busy house, we have grandchildren and people coming and going so sometimes babies are too young, babies might be a bit put off by her, so you might have to put her out and so she would sort of get to know whats sort of acceptable and whats not. some the other older grandchildren love her and she'll let them do anything to her and she's very good natured and placid kind off animal. will get a little bit excited around children and thats why we have to be carefull, she would unintentionally knock them over or something like that. the grand children all look forward to coming to see her she is very much part of the house, but the children will always have to come first in terms of you don't want to take any risks.

Participant 7

Well each one of the dogs has their own personality each one is different in their own way. We have so many of them that you can't spend time with them but they have a home and they're all loved in their own way. and thats the most important thing as well. We have 3 cats, and if another one turned up, I couldn't say know, there will always be a space there I think. I love all the cats I love all the dogs, but as I said my first love was always cats but the dogs now I always love them, I would misstreat no animal, even a rat, because I think every animal has a right in this world, people do illtreat cats , because we had cat and she went out and when she came back her belly and legs were shaved. I said to Mr. Rafter (old vet) he couldn't believe it, she wouldn't have let me shave her, he said it was very unusual, didn't know what happened her. (Laughter etc) Mosses he got poisoned, we only had him home 3 days after saving him and he got poisoned again, and he couldn't be saved his brother got poisoned again and he was in the vets for 3 days, someone had put slugg tox down its troath, a cat would not eat slugg tox, a dog will, so the only way was if someone shoved it down his troath,

(General chat about the cat that was put in the wheelie bin, mainly wishing her bad luck....)

Participant 3

I think it is just hugely important to both to excercise and to play with the animals, I think physical contact is very very important at a very early age with thte animals because otherwise how can you bring the animal into the vet and expect the animal to just sit there and be handled by the vet, you know, and certainly with the dogs there is lots of playing, ad mad zany westie is the best of fun, an absolute tonic, we have a play session, usually before we go for a walk, once he sees the lead he goes mad, and he runs around me all this caper goes on, it's like there are 10 people in the house. and then when we get back it is more or less the same, I find in the evening around 8 oclock when he is absolutely exhausted and I sit down to watch telly, he'll just climb up on my knee and thats it, he likes to get on the knee, it's just at the end of the day and I think maybe he just likes that physical contact, but I have to say I like that time of day too, I think it is a really nice thing between the 2 of us, I was thinking if someone was to ask me what were the happiest moments I've had over the last few years I would say the golden moments I've had over the last few years have beenthe times I've been out walking the dog, because I think when your out there, phnes can't get at you, the door bell can't get at you nobody can get at you, your just at one out there with the dog, just the 2 of you. there is just this sort of mental bonding just this oneness with the 2 of you, I see you nodding your head Participant 2

, it's an amazing feeling it really, There's something primal or whatever about it one man or one woman and her dog. For me it is a really happy time when I'm out walking with the dog, the rest of the world is sort of outsid ethat. And you certainly get back what you give out to an animal, a 100 fold. I think thats the majic.

Participant 8

Some times as well when you have a rescue animal you almost feel like you have a double responsibility, that you owe them because they had a rotten start

Participant 3

But then you get 200% back, Peole with rescues and rehomes will always say to you you get 200% back

Participant 2

I think I can understand that, some people say that dogswill care for you the way cats won't, but cats do care for you, and the rescue cats do. Last winter there was one day I couldn't get out of bed I was so sick, and the tabby who is very kitteny who is usually gone off for the day or attacks my feet through the sheets, he came up and never moved off of my bed. The only time he got up was when I crawled out of bed at 5 oclock to go to feed the puppy. and then he was back on my bed and stayed there the rest of the day. I thought thats an amazing carer, from a cat, usuually people say cats are aloof.

Participant 3

They're the majic in our lifes

Participant 4

There is loads of communication with the dogs in my house, and especially with the 2 of the kids, because theyr all into their music, and when the music starts in the evening time, they'd probably have the great dane up on his back legs dancing with one of them. there is always someone with them or talking to them, and then the young one she'll lie down beside them and hug them before she goes to bed, and when everyone is in sitting in watching a football match or a movie the dogs actually sit right beside you, they'll see who is in the best form and sit beside them.
